# Cuticle Integrity and Biogenic Amine Synthesis in *Caenorhabditis elegans* Require the Cofactor Tetrahydrobiopterin (BH4)

**DOI:** 10.1534/genetics.114.174110

**Published:** 2015-03-24

**Authors:** Curtis M. Loer, Ana C. Calvo, Katrin Watschinger, Gabriele Werner-Felmayer, Delia O’Rourke, Dave Stroud, Amy Tong, Jennifer R. Gotenstein, Andrew D. Chisholm, Jonathan Hodgkin, Ernst R. Werner, Aurora Martinez

**Affiliations:** * Department of Biology, University of San Diego, San Diego, California, 92110; ** Division of Biological Sciences, University of California, San Diego, California 92093; † Department of Biomedicine, University of Bergen, 5009 Bergen, Norway; ‡ Division of Biological Chemistry, Biocenter, Innsbruck Medical University, A-6020 Innsbruck, Austria; § Department of Biochemistry, University of Oxford, Oxford OX1 3QU, United Kingdom

**Keywords:** biopterin, epidermis, serotonin, dopamine, GTPCH, alkylglycerol monooxygenase, AGMO

## Abstract

Tetrahydrobiopterin (BH4) is the natural cofactor of several enzymes widely distributed among eukaryotes, including aromatic amino acid hydroxylases (AAAHs), nitric oxide synthases (NOSs), and alkylglycerol monooxygenase (AGMO). We show here that the nematode *Caenorhabditis elegans*, which has three AAAH genes and one AGMO gene, contains BH4 and has genes that function in BH4 synthesis and regeneration. Knockout mutants for putative BH4 synthetic enzyme genes lack the predicted enzymatic activities, synthesize no BH4, and have indistinguishable behavioral and neurotransmitter phenotypes, including serotonin and dopamine deficiency. The BH4 regeneration enzymes are not required for steady-state levels of biogenic amines, but become rate limiting in conditions of reduced BH4 synthesis. BH4-deficient mutants also have a fragile cuticle and are generally hypersensitive to exogenous agents, a phenotype that is not due to AAAH deficiency, but rather to dysfunction in the lipid metabolic enzyme AGMO, which is expressed in the epidermis. Loss of AGMO or BH4 synthesis also specifically alters the sensitivity of *C. elegans* to bacterial pathogens, revealing a cuticular function for AGMO-dependent lipid metabolism in host–pathogen interactions.

TETRAHYDROBIOPTERIN (BH4; 6R-5,6,7,8-tetrahydrobiopterin) is the natural cofactor of three distinct classes of enzymes including the aromatic amino acid hydroxylases (AAAHs), nitric oxide synthases (NOSs), and alkylglycerol monooxygenase (AGMO) (Werner *et al.* 2011). BH4 is therefore critical for a variety of cellular processes, being essential for the conversion of L-Phe to L-Tyr, for alkyl ether lipid metabolism, for synthesis of nitric oxide (NO), and synthesis of the neurotransmitters serotonin (5-hydroxytryptamine, 5HT) and dopamine (DA) and their derivatives. BH4 is present in many eukaryotes (Werner-Felmayer *et al.* 2002), including the nematode *Caenorhabditis elegans* (Calvo *et al.* 2008), in which the functions of the AAAHs phenylalanine hydroxylase (PAH, gene *pah-1*), tyrosine hydroxylase (TH, gene *cat-2*), and tryptophan hydroxylase (TPH, gene *tph-1*) are well established (Lints and Emmons 1999; Loer *et al.* 1999; Sze *et al.* 2000; Calvo *et al.* 2008). *C. elegans* lacks an endogenous NOS (Gusarov *et al.* 2013); as shown below, *C. elegans* encodes a single ortholog of the recently characterized AGMO (Watschinger *et al.* 2010).

In mammals, BH4 is synthesized *de novo* in four steps from GTP by at least three enzymes: GTP cyclohydrolase I (GTPCH1, human gene *GCH1*), 6-pyruvoyl tetrahydrobiopterin synthetase (PTPS, human gene *PTS*), and either sepiapterin reductase (SR), carbonyl reductase, and/or aldose reductase (Figure 1, Table 1; Werner *et al.* 2011). BH4 synthesis is regulated through the action of the GTPCH1 feedback regulatory protein (GFRP), known to mediate the activation or inhibition of mammalian GTPCH1 by L-Phe or BH4, respectively. In humans, mutations in the *GCH1* gene can be recessive or cause a dominant Dopa-responsive dystonia, with or without hyperphenylalaninemia (HPA) (Ichinose *et al.* 1999). Mutations in the *PTS* gene lead to BH4-deficient HPA (also called atypical HPA or malignant phenylketonuria) (Thöny and Blau 1997). In BH4-deficient HPA patients, the neurological symptoms vary in severity depending on the degree of reduction in biogenic amine and nitric oxide levels. These conditions are manageable by carefully monitored biopterin supplementation and other treatments (Blau *et al.* 2001; Longo 2009). *Pts* knockout mice die within 48 hr if untreated with BH4 and neurotransmitter precursors (Sumi-Ichinose *et al.* 2001; Elzaouk *et al.* 2003).

**Figure 1:**
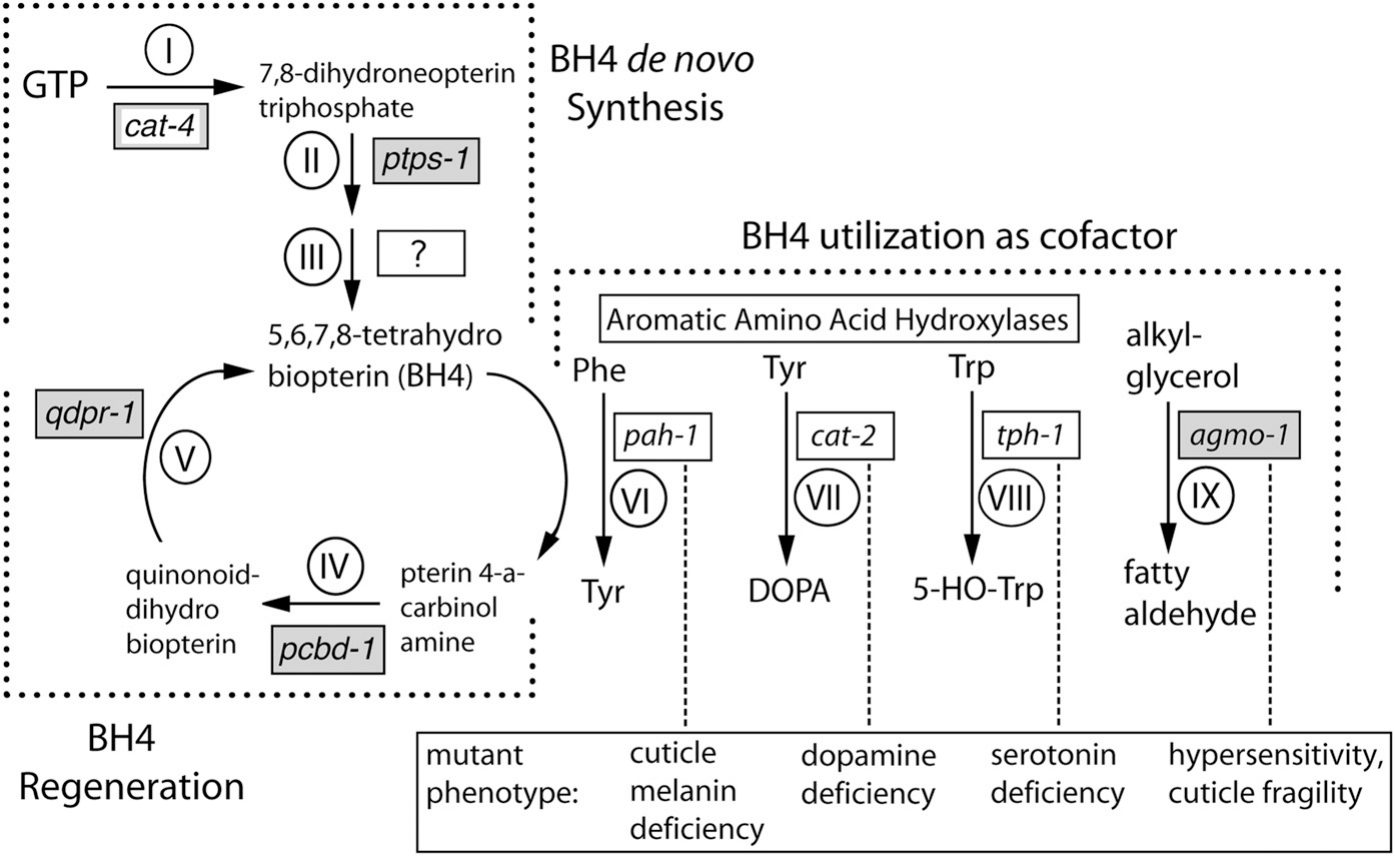
Biosynthesis, regeneration and utilization of tetrahydrobiopterin (BH4), and functions of BH4-dependent enzymes in *C. elegans*. Enzymes catalyzing pathway reactions are indicated by Roman numerals (Werner *et al.* 2011). (I) GTP cyclohydrolase I (E.C. 3.5.4.16); (II) 6-pyruvoyl tetrahydropterin synthase (E.C. 4.2.3.12); (III) sepiapterin reductase (E.C. 1.1.1.153), (IV), pterin-4a-carbinolamine dehydratase (E.C. 4.2.1.96); (V) [quinoid] dihydropteridine reductase (E.C. 1.6.99.7); (VI) phenylalanine hydroxylase (E.C. 1.14.16.1); (VII) tyrosine hydroxylase (E.C. 1.14.16.2); (VIII) tryptophan hydroxylase (E.C. 1.14.16.4); and (IX) alkylglycerol monooxygenase (E.C. 1.14.16.5). Names of *C. elegans* genes encoding these enzymes are shown in boxes adjacent to Roman numerals; gray boxes indicate genes for which knockout mutants are described for the first time in this work. Although *cat-4* mutant phenotypes have been described previously, we demonstrate here that the gene encodes GTPCH1. Top left: Pathway for *de novo* BH4 synthesis. The gene encoding the enzyme catalyzing the final step(s) in BH4 synthesis is unknown. Bottom left: Pathway for BH4 regeneration. Right: Four enzymes that use BH4. Mutants in *cat-4* and *ptps-1* genes (BH4-deficient) have all phenotypes listed in the box at bottom. Mutants in individual BH4-dependent enzyme genes have the indicated subset of BH4-deficiency phenotype (dashed line).

**Table 1:** Pterin synthesis, regeneration, and related genes in *C. elegans*

Protein	*C. elegans* gene name	Genetic location / Genomic position (coding)	BLAST2 Expected-value *vs.* human and fly proteins*	Mutant allele(s)
GTP cyclohydrolase I (GTPCH1)	*cat-4* / F32G8.6	V: 2.59 V: 10,564,851-10,567,502 bp	*Hs*: 7 × 10^−84^ *Dm*: 6 × 10^−81^	*e1141*, *e3015*, *e3030*, *ok342*, *tm773*
				
Pyruvoyl tetrahydropteridine synthase (PTPS)	*ptps-1* / B0041.6	I: -1.03 I: 4,652,907-4,652,187 bp	*Hs*: 9 × 10^−39^ *Dm*: 8 × 10^−44^	*e3042*, *tm1984*
				
Sepiapterin reductase (SR)	No ortholog	NA	**Best matches in *Ce* by BLASTP *Hs* SR: 6 × 10^−9^ *Dm* SR: 9 × 10^−12^	NA
				
Carbonyl reductase (CR)	No ortholog	NA	**Best match in *Ce* by BLASTP *Hs* CBR1: 2 × 10^−12^	NA
				
Aldose reductase (AR)	Y39G8B.1	II: 21.67 II: 13,970,583-13,972,946 bp	*Hs* AKR1B1: 2 × 10^−97^ *Hs* AKR1C3: 1 × 10^−87^	*ok1682*
				
Pterin carbinolamine dehydratase (PCBD)	*pcbd-1* / T10B11.1	I: 1.57 I: 6,951,134-6,951,946 bp	*Hs*: 9 × 10^−39^ *Dm*: 2 × 10^−37^	*tm5924*
				
Quinoid dihydropteridine reductase (QDPR)	*qdpr-1* / T03F6.1	III: 21.21 III: 13,393,783-13,394,837 bp	*Hs*: 3 × 10^−70^ *Dm*: 3 × 10^−77^	*tm2337*, *tm2373*
				
Dihydrofolate reductase (DHFR)	*dhfr-1* / C36B1.7	I: 3.04 I: 8,736,060-8,737,028 bp	*Hs*: 3 × 10^−34^ *Dm*: 3 × 10^−36^	None
				
GTP cyclohydrolase I feedback regulatory protein (GFRP)	*gfrp-1* / Y38C1AA.13	IV: -26.81	*Hs*: 5 × 10^−28^ *Dm*: no GFRP	None
		IV: 203,882-207,984 bp		

*BLAST2: For multiple isoforms, the score shown is the best match between a *Ce* predicted protein and *Hs* (human) or *Dm* (fruit fly) protein. **Best match of sepiapterin reductase (SR) or carbonyl reductase (CR) via BLASTP to *Ce* proteins (nr database). Both CR and aldose reductase are possible partial substitutes for SR. NA, not applicable.

As well as being required for neurotransmitter synthesis, BH4 and its derivatives are important for the synthesis of pigments and quinones involved in cross-linking external cuticle layers in invertebrates (Iino *et al.* 2000; Kato *et al.* 2006). The molecular genetics of biopterin synthesis and biogenic amine metabolism have been extensively characterized in *Drosophila melanogaster* (Wright 1987; O’Donnell *et al.* 1989). In *Drosophila*, where dopa and dopamine are required for sclerotization and tanning of the cuticle (Neckameyer and White 1993), knockout *Punch* (GTPCH1) and *purple* (PTPS) mutants die as embryos due to severe cuticle abnormalities and/or a requirement for serotonin in germband extension (Mackay and O’Donnell 1983; Colas *et al.* 1999). Interestingly, GFRP is not found in *Drosophila* (Funderburk *et al.* 2006).

When BH4 is used by the AAAHs and AGMO in their respective hydroxylation reactions, it is oxidized to pterin 4-a-carbinolamine (Figure 1). This oxidized biopterin can be regenerated in mammals to BH4 by two reduction steps catalyzed by pterin carbinolamine dehydratase (PCBD) and quinoid dihydropteridine reductase (QDPR) (Werner *et al.* 2011). In humans, mutations in *QDPR* have severe effects like those in *GCH1* and *PTS* genes, whereas mutation in the human *PCDB1* gene yields a milder clinical picture (Opladen *et al.* 2012). BH4 can also be oxidized nonenzymatically and subsequently regenerated to BH4 by dihydrofolate reductase (DHFR) (Werner *et al.* 2011; Xu *et al.* 2014).

Analysis of the complete genomic sequence of *C. elegans* reveals orthologs of genes encoding biopterin synthesis, regulation, and regeneration enzymes known from other animals (Figure 1 and Table 1; see also Hobert 2013). *C. elegans* has clear orthologs encoding the first two BH4 synthetic enzymes, GTP cyclohydrolase I (GTPCH1, gene *cat-4*) and 6-pyruvoyl-tetrahydropterin synthase (PTPS, gene *ptps-1*). *C. elegans* appears to lack an ortholog of sepiapterin reductase (Kallberg *et al.* 2002), but does encode an aldose reductase (Table 1), which can partially substitute for SR in mammals (Park *et al.* 1991; Iino *et al.* 2003). The *C. elegans* genome also contains genes encoding biopterin regeneration enzymes PCBD, QDPR, and DHFR (genes *pcbd-1*, *qdpr-1*, and *dhfr-1*) and a clear ortholog of GFRP (*gfrp-1*).

Although *cat-4* mutants were isolated in 1975 (Sulston *et al.* 1975), the biochemical genetics of BH4 in *C. elegans* has not been previously examined. *cat-4* mutants were found based on their lack of the catecholamine DA (Sulston *et al.* 1975) and were subsequently found to be 5HT deficient (Desai *et al.* 1988). These neurotransmitter deficiencies in *cat-4* mutants cause a variety of subtle behavioral abnormalities, including defective locomotory rate regulation and male mating (Loer and Kenyon 1993; Sawin *et al.* 2000). We and others have also found that *cat-4* mutants are hypersensitive to a variety of agents, suggesting they might have a generally “leaky” cuticle (Loer 1995; Weinshenker *et al.* 1995; Cronin *et al.* 2005; Baker *et al.* 2012). A mechanistic explanation for the cuticle defects in *cat-4* mutants, however, has been lacking.

Here we characterize the *C. elegans* pathway for BH4 biosynthesis, regulation, and regeneration. We find that *cat-4* and *ptps-1* mutants are biopterin-, 5HT- and DA-deficient and lack GTPCH1 and PTPS activities, as predicted. BH4-deficient animals have a fragile cuticle that is more permeable to small molecules, resulting in hypersensitivity to multiple chemicals. As deletion mutants in the AAAH genes do not display cuticle fragility or chemical hypersensitivity, we inferred that these phenotypes might reflect impaired function in another BH4-dependent enzyme. We show here that loss of function in the biopterin-dependent lipid metabolic enzyme AGMO (Watschinger *et al.* 2010) results in chemical hypersensitivity and cuticle fragility like that observed in BH4-deficient mutants. We find that *agmo-1* is expressed in the epidermis, consistent with its requirement for a BH4 cofactor and its role in cuticle integrity. Furthermore, *agmo-1* and the BH4-deficient mutants share a common phenotype of sensitivity to bacterial infection by *Leucobacter* Verde1. Our studies provide the first *in vivo* evidence for a role for AGMO in epidermal lipid metabolism and in pathogen defense, with implications for the function of this enzyme in other animals and in humans.

## Materials and Methods

### *C. elegans* culture, strains, and transgenes

Routine culturing of *C. elegans* was performed as described (Brenner 1974); strains were grown at 20° for all experiments, although acute analyses were at room temperature (RT) (21°–23°). Nomenclature used for *C. elegans* genetics conforms to conventions (Horvitz *et al.* 1979). Worm strains used are listed in Supporting Information. Deletion mutant strains of *cat-4*, *ptps-1*, *qdpr-1*, *pcbd-1*, and *agmo-1* were outcrossed three to five times before most analyses. Homozygosity of deletion strains was confirmed by PCR.

We generated transgenes for *cat-4*, *ptps-1*, *pcbd-1*, *qdpr-1*, *gfrp-1*, *pah-1*, and *agmo-1* by the duplex PCR method (Hobert 2002); see Table S1 on transgenes and transgenics. Amplified genomic DNAs were fused to amplified GFP; duplex products were co-injected with P*ttx-3*::RFP marker plasmid into wild type to generate transgenics. We also examined reporter transgenics previously described by others (Table S2).

### The *cat-4* mutant allele sequencing and bioinformatics

The *cat-4(e1141)* mutation was identified by PCR-amplifying exons from genomic CB1141 DNA, then Sanger sequencing the purified PCR product as described (Hare and Loer 2004). The mutation was confirmed by sequencing both strands for two independent PCR reactions. The *cat-4(gk245686)* mutation (strain VC20144) was confirmed in the same manner. PCR and sequencing primers were designed using Primer3 (http://www-genome.wi.mit.edu/cgi-bin/primer/primer3.cgi).

*C. elegans* genomic and predicted cDNA sequences were retrieved from WormBase and/or GenBank. Blast searches and Blast2 comparisons were performed using the NCBI Blast server. Multiple sequence alignments used CLUSTALW (www.ebi.ac.uk/clustalw/).

### Detection of serotonin and dopamine *in situ*

Serotonin was assessed in whole-mount worms by immunofluorescence as previously described (Desai *et al.* 1988; Loer and Kenyon 1993) using a polyclonal antiserum against 5HT paraformaldehyde conjugated to BSA (Sigma, St. Louis; catalog no. E5545, lot 091K4831) previously tested for specificity (Loer and Rivard 2007). DA was assessed in whole-mount worms via formaldehyde-induced fluorescence (FIF) as previously described (Lints and Emmons 1999). Treatment of worms with 5-hydroxytryptophan (5-HTP) or L-dopa prior to staining was done as described (Rivard *et al.* 2010).

### Chemical sensitivity and cuticle integrity tests and male mating assays

Most chemicals were obtained from Sigma-Aldrich, including levamisole (T512), 5-hydroxytryptophan, and L-dopa. BH4 and sepiapterin were obtained from B. Schircks Laboratories (Jona, Switzerland). We tested adult hermaphrodites grown for 3 days at 20° from synchronized L1 worms hatched in M9. Eggs isolated by bleaching gravid adult hermaphrodites were washed extensively with M9, then hatched in M9 at RT (22°–23°). Starved L1 worms were used immediately or stored at 12°–13°. Synchronized L1s were used no later than 7 days after egg isolation. Adult worms were washed from plates with M9 and 500-µl aliquots placed in 24-well plates. Because of “swimming-induced paralysis,” which can vary based on biogenic amine levels (McDonald *et al.* 2007), wells were scored after approximately the same length of time in M9 buffer. Chemicals (SDS, levamisole) were made as 2× solutions in M9; 500 µl of 2× solution was added to a well with 500 µl worms (50–100) to yield a working concentration. Individual worms in a well were observed through a stereomicroscope and scored as immobile if no movement was seen during the brief moment of observation (≤1 sec).

Rapid bleach hypersensitivity tests were done with standard *C. elegans* “alkaline bleach” [4.5% sodium hypochlorite (household bleach)/1M NaOH]. Gravid adult hermaphrodites were tested on NGM plates by applying a ∼5-µl drop of bleach on a worm and assaying time to rigidity using a stereomicroscope. Cuticle disintegration assays were carried out as described (Calvo *et al.* 2008). Briefly, gravid adults placed in a milder alkaline bleach solution (1% sodium hypochlorite, 0.25 M NaOH) were observed under a dissecting stereomicroscope, and the time of first major break in the cuticle was recorded. Plates were agitated manually every 30 sec. A total of 15–30 worms were scored in every experiment.

### Preparation of worm homogenates and the effect of BH4 supplementation

Nematode cultures were grown on NGM plates supplemented with 5 mM ascorbate, 200 μM BH4 for 3–5 days (until food was almost depleted). Mixed-stage populations (nonsynchronized, with predominance of adult worms) were recovered from plates with sterile M9 buffer and cleaned of bacteria by successive centrifugations at 1000 rpm. Finally, worms were incubated 30 min in M9 with agitation to reduce bacteria in the gut and centrifuged again; the resulting pellet was immediately frozen in liquid nitrogen for later use. The frozen worm pellet was resuspended in 400 µl distilled water containing 5 mM dithioerythritol, homogenized with an Ultraturrax (Iba, Stauffen, Germany), frozen in liquid nitrogen, thawed, homogenized again, and centrifuged 5 min at 16,000 × *g* at 4°.

### Determination of GTPCH1 and PTPS activity and BH4 content

GTPCH1 and PTPS enzymatic assays were carried out as described (Werner *et al.* 1997; Heller *et al.* 2001), with modifications to reduce the amount of required material. Results were related to protein concentrations measured in homogenates and eluates by Bradford assay (Biorad, Vienna). For BH4 determinations, 50 µl of homogenate was used for both iodine oxidation in acid and alkaline medium assays (Heller *et al.* 2001), and BH4 concentration was calculated from the difference of resulting biopterin from the two oxidation schemes (Fukushima and Nixon 1980). For acidic oxidation, 50 µl homogenate was mixed with 5 µl 1 M HCl and 5 µl 0.1 M iodine solution (prepared in 0.25 M potassium iodide). For basic oxidation, HCl was replaced by 1 M NaOH. After 60 min at RT in the dark, 10 µl 1 M HCl was added to the alkaline oxidation only, both incubations were centrifuged for 5 min at 16,000 × *g* at 4°, and supernatants added to 10 µl freshly prepared 0.1 M ascorbic acid. Biopterin was measured by HPLC after injection of 10 µl on a Nucleosil 10 SA column (250 mm long, 4 mm inner diameter, 10 µm particle size; Macherey Nagl, Düren, Germany), elution with 50 mM potassium phosphate, pH 3.0 at 35° and fluorescence detection (Excitation 350 nm, Emission 440 nm) with an Agilent 1200 HPLC (Agilent, Vienna).

For determining GTPCH1 activity, 80 µl of homogenate was separated from low MW compounds using Micro Bio-Spin 6 columns (Biorad) equilibrated to GTPCH1 assay buffer (100 mM Tris-HCl, pH 7.8, 2.5 mM EDTA, 300 mM KCl, 10% (v/v) glycerol), incubated with 1.5 mM GTP for 90 min at 37° in 85.7 µl total volume. Reaction was stopped by addition of 2.85 µl 1 M HCl and 2.85 µl 0.1 M iodine. Oxidation of resulting 7,8-dihydroneopterin triphosphate to neopterin triphosphate was achieved by incubation for 60 min at RT in the dark. After 2 min centrifugation at 16,000 × *g* at 4°, 2.85 µl 0.1 M ascorbic acid was added to the supernatant. After neutralization by addition of 2.85 µl 1 M NaOH, neopterin phosphates were cleaved to neopterin by incubation with 6.4 units alkaline phosphatase for 30 min at 37°. Neopterin was quantified by HPLC using 10 µl final reaction mixture injected on a reversed phase C-18 column (250 mm long, 4 mm inner diameter, 5 µm particle size; Lichrosphere, Merck, Darmstadt, Germany), eluted with 15 mM potassium phosphate, pH 6.0, at 25° and fluorescence detection (Ex 350 nm, Em 440 nm).

For measuring PTPS activity, 80 µl of homogenate was separated from low MW compounds using Micro Bio-Spin 6 columns (Biorad) equilibrated to PTPS assay buffer (0.1 M Tris-HCl, pH 7.4, 20 mM MgCl_2_), and incubated with 40 µM freshly prepared 7,8-dihydroneopterin triphosphate (using recombinant *Escherichia coli* GTPCH1) and 2 mM NADPH in the presence of *E. coli*-expressed recombinant mouse SR (2 nmol/min) in 100 µl total volume for 1 h at 37° (Werner *et al.* 1997). Reaction was stopped by addition of 5 µl 1 M HCl, and 5 µl 0.1 M iodine. Following further incubation for 1 h at RT in the dark, resulting biopterin was quantified by HPLC as for neopterin in the GTPCH1 assay described above.

### Isolation of *Leucobacter* Verde1 resistance mutants and whole genome sequencing

Selections for increased resistance to *Leucobacter* Verde1 exploited the hypersensitivity of certain *bus* and *srf* mutants to this bacterial pathogen, which is completely lethal to such mutants but not to wild-type *C. elegans* (Hodgkin *et al.* 2013). Mutants in three such genes (*bus-10*, *srf-2*, and *srf-5*) were used initially. Populations of these mutants were mutagenized with 0.05 M EMS (Brenner 1974). After mutagenesis, 50–80 individual L4 worms were picked to separate plates and grown on *E. coli* OP50 for two generations. About 200 F_2_ progeny from each plate were then transferred to mixed *E. coli/*Verde1 (10:1) lawns and incubated at RT for a further 7–10 days, after which most (273/300) plates contained only dead or dying worms. Plates with surviving fertile worms were retained, and single worms were picked from these to establish independent resistant lines. Outcrossing established that most resistant lines carried recessive extragenic mutations conferring resistance to Verde1 and increased bleach sensitivity. Genetic mapping and complementation tests, utilizing the bleach sensitivity phenotype, assigned 27 independent mutations to nine complementation groups. The largest complementation group (eight alleles) was mapped genetically to the right arm of LGIII. DNA from a mutant strain carrying one of these mutations (*e3016*) was prepared for whole genome sequencing using a standard library preparation without amplification, followed by Illumina 50-bp paired end sequencing on the HiSeq2000. Candidate mutations were identified using MAQGene (Bigelow *et al.* 2009), which revealed a nonsense mutation (W130opal) in *agmo-1*. Specific sequencing of *agmo-1* in strains for other alleles in this complementation group showed that all carried predicted severe mutations in this gene. Similarly, two other complementation groups were found to correspond to *cat-4* (alleles *e3015* and *e3030*) and *ptps-1(e3042)*. Detailed analyses of six other complementation groups will be presented elsewhere.

## Results

### The *C. elegans cat-4* (catecholamine defective) gene encodes GTP cyclohydrolase I (GTPCH1)

**Figure 2:**
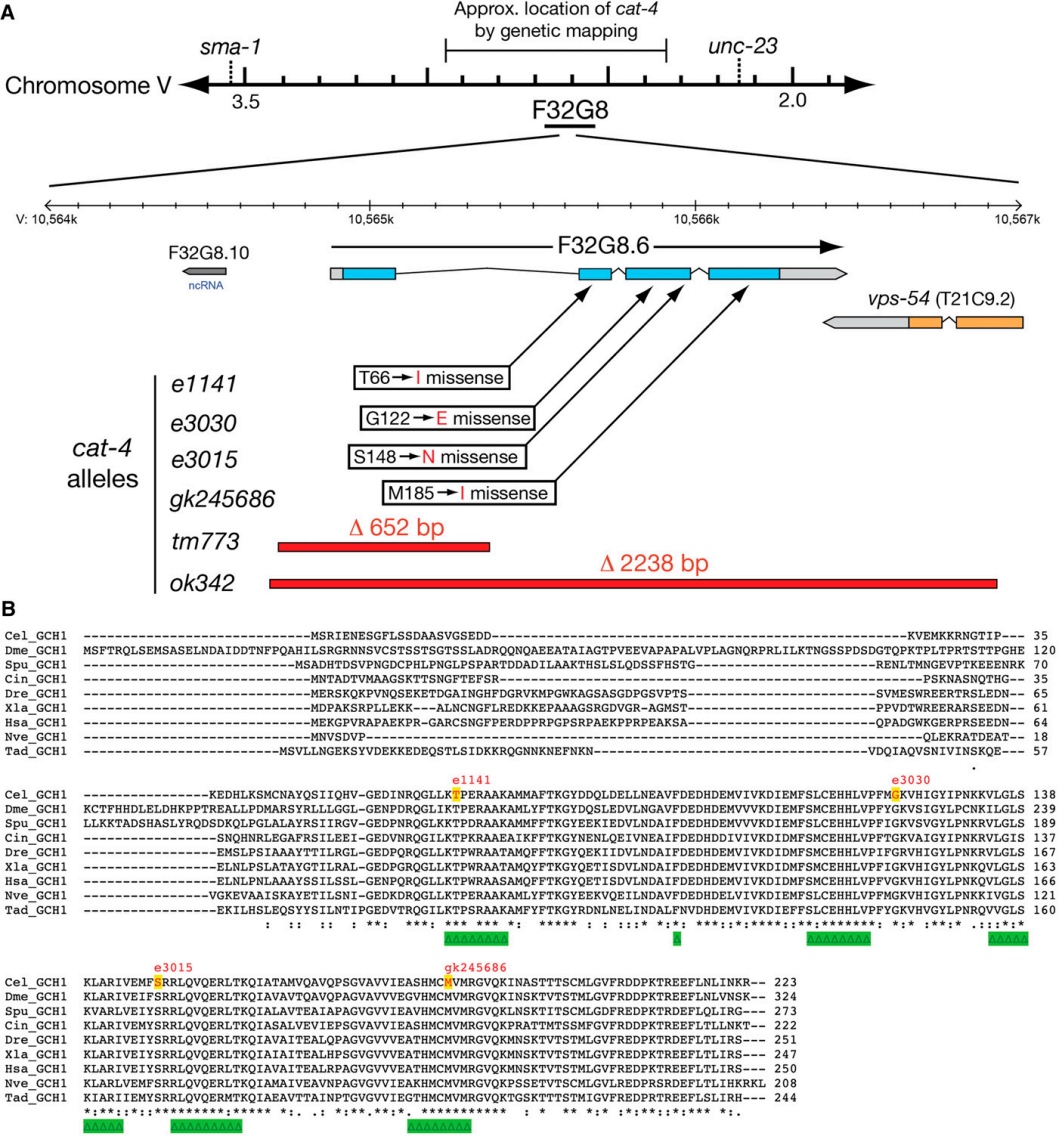
The *cat-4* gene encodes GTP cyclohydrolase I (GTPCH1). (A) Maps of *cat-4* region with mutant alleles and gene structure. *cat-4(e1141)* maps genetically between *sma-1* and *unc-23* on chromosome V in the same region as gene F32G8.6. Nature of *cat-4* alleles is shown below and approximate locations are indicated with arrows (point mutations) or red bars (deletions). Sequencing of *cat-4* cDNAs confirms the gene model shown (Figure S1) and the predicted proteins used for alignments. Image is partly derived from the WormBase genome browser. (B) Alignment of *C. elegans* CAT-4 with GTPCH1 proteins from other metazoans. Asterisks below alignment show 100% conserved residues; colon indicates conserved highly similar residues; and period indicates conserved weakly similar residues. Green triangles indicate amino acids likely within the active site (Maita *et al.* 2004). Residues altered in *cat-4* mutant alleles are marked with red letters/yellow backgrounds. Species abbreviations are as follows: Cel, *C. elegans*; Dme, *Drosophila melanogaster*; Spu, *Strongylocentrotus purpuratus*; Cin, *Ciona intestinalis*; Dre, *Danio rerio*; Xla, *Xenopus laevis*; has, *Homo sapiens*; Nve, *Nematostella vectensis* (a partial sequence); and Tad, *Trichoplax adherens*.

The *cat-4* gene maps genetically near a predicted GTPCH1-encoding gene (F32G8.6) on chromosome V. Sequencing F32G8.6 exons from the original allele *cat-4(e1141)*, revealed a missense mutation that changes an amino acid (T66I) that is 100% conserved in GTPCH1 proteins (Figure 2, Figure S1). Two putative knockout mutants with deletions (*ok342* and *tm773*) in F32G8.6 failed to complement *e1141*, were also 5HT- and DA-deficient, and were hypersensitive with a fragile cuticle (Table 2). We examined additional *cat-4* mutants with missense mutations altering different highly conserved amino acids (Figure 2); these mutants showed a range of phenotypes. *cat-4(e3030)* mutants had little or no apparent 5HT or DA, whereas *e3015* mutants had reduced 5HT and DA.

**Table 2:** Phenotypes of *C. elegans* pterin-related gene mutants

*C. elegans* strain	Alleles tested	Serotonin*^a^*	Dopamine*^b^*	Bleach hypersensitivity
N2 (wild type)	NA	+*^c^*	+	Non Hyp
				
*cat-4(−)* [GTPCH1]	*e1141*, *ok342*, *tm773*, *e3030*	–*^d^*	–	Hyp
				
*ptps-1(−)*	*tm1984*, *e3042*	–	–	Hyp
				
*pcbd-1(−)*	*tm5924*	+/–	+/–	Non Hyp
				
*qdpr-1(−)*	*tm2337*, *tm2373*	+/–	+/–	Non Hyp
				
*tph-1(−)*	*^e^*	–	+	Non Hyp
				
*cat-2(−)* [TH]	*^e^*	+	–	Non Hyp
				
*bas-1(−)* [AADC]	*^e^*	–	–	Non Hyp
				
*pah-1(−)*	*^e^*	+	+	Non Hyp
				
*agmo-1(−)*	*e3016*, *e3019*, *e3029*, *e3047*	+	+	Hyp
				
Y39G8B.1(*−*) [AR]*^f^*	*ok1682*	+	+	Non Hyp

NA, not applicable.

a Tested by immunoreactivity (IR).

b Tested by formaldehyde induced fluorescence (FIF).

c Wild type.

d Deficient.

e Reported previously.

f Y39G8B.1 encodes an aldose reductase (AR) ortholog, a possible partial substitute for sepiapterin reductase. The putative mutant, however, shows no phenotypes consistent with BH4 deficiency.

Bleach hypersensitivity and reduced melanin phenotypes of *cat-4(tm773)* mutant worms are rescued in transgenics carrying a plasmid containing F32G8.6 coding sequence, 3′-UTR, and 1500 bp upstream (Baker *et al.* 2012). We tested these transgenic worms for 5HT and DA expression; in two independent transgenic lines carrying extrachromosomal arrays, a high percentage of worms were rescued for DA and 5HT in all normal dopaminergic and serotonergic neurons (Figure S2).

### Mutants defective in BH4 synthesis display biogenic amine deficiency, chemical hypersensitivity, and cuticle fragility

**Figure 3:**
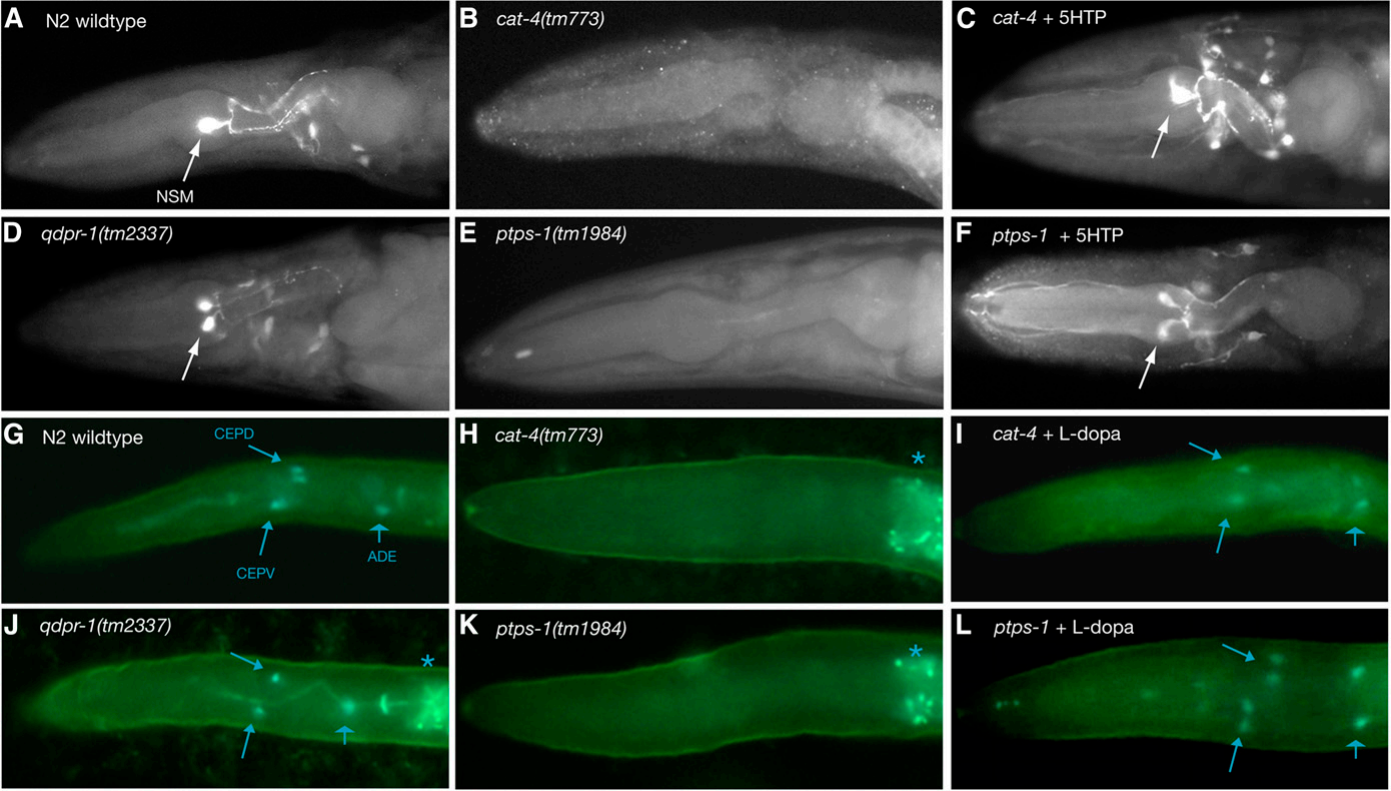
Neurotransmitter phenotypes of *cat-4*, *ptps-1*, and *qdpr-1* mutants. (A–F) Anti-5HT immunofluorescence of adult worm heads. One prominent 5HT neuron cell body (neurosecretory motorneuron, NSM) is marked with an arrow. Anterior is to the left. (A) Wild type (N2) and (D) *qdpr-1* mutants have normal 5HT; (B) *cat-4* and (E) *ptps-1* mutants lack 5HT. (C and F) 5HT-IR is restored in *cat-4* and *ptps-1* mutants by treatment with 5-hydroxytryptophan (5-HTP). 5-HTP also causes dopaminergic neurons to make 5HT. [Rescue of *cat-4* mutants by 5-HTP has been shown previously (Loer and Kenyon 1993).] (G–L) FIF showing dopamine (DA) in larval worm heads. DA-containing cells have characteristic blue-green fluorescence; background is more yellow-green. DA cells in the head are indicated with arrows (CEPD and CEPV, smaller arrowhead; and ADE, broad arrowhead). Asterisks indicate nonspecific intestinal fluorescence. In some heads, one can see both right and left side DA neurons. Anterior is to the left. (G) Wild type (N2) and (J) *qdpr-1* mutants have normal DA; (H) *cat-4* and (K) *ptps-1* mutants lack DA. (I and L) DA fluorescence is restored in *cat-4* and *ptps-1* mutants by treatment with L-dopa.

**Figure 4:**
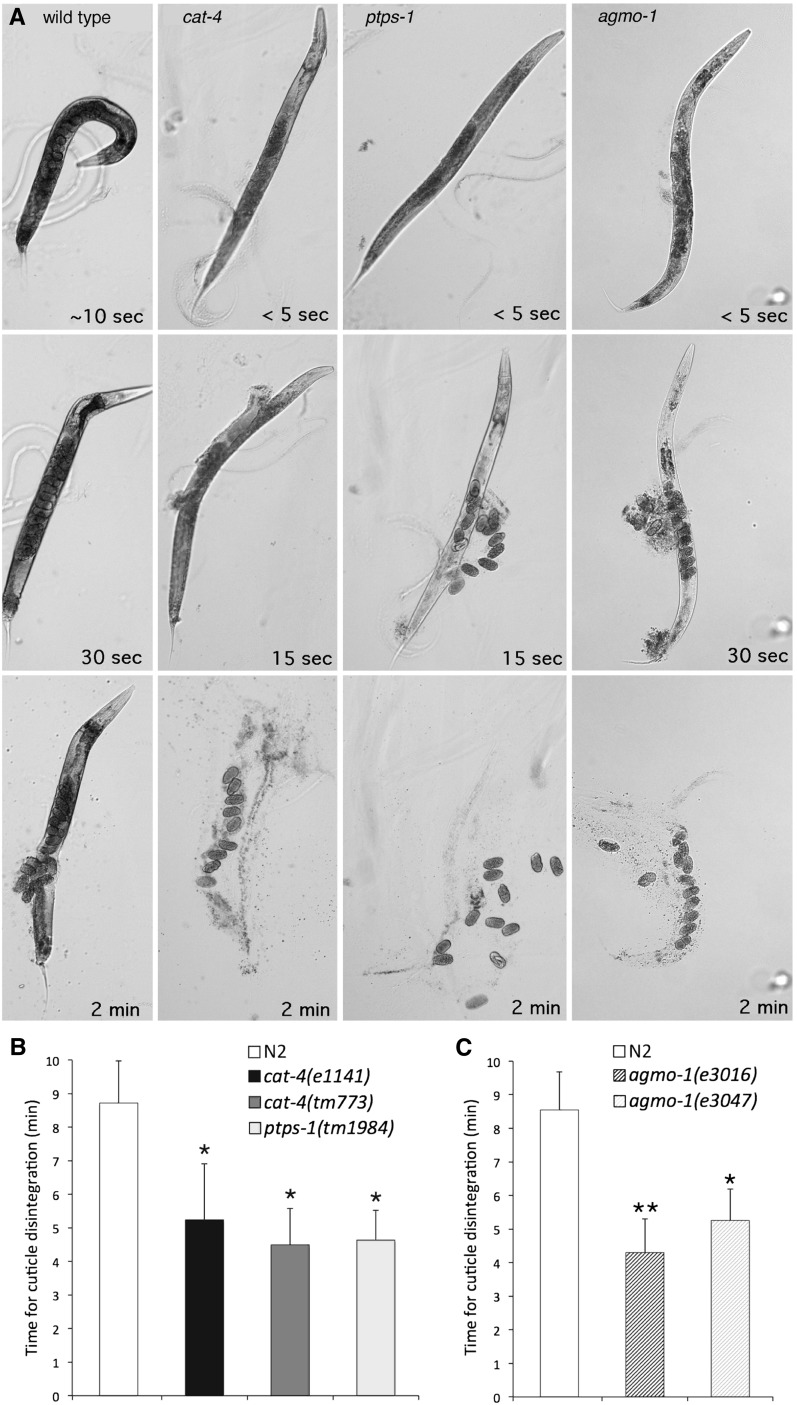
*cat-4*, *ptps-1*, and *agmo-1* mutants share a common hypersensitivity to exogenous chemicals. (A) Hypersensitivity and cuticle fragility shown by rapid death and disintegration of *cat-4*, *ptps-1*, and *agmo-1* mutants in standard alkaline bleach. Adult gravid hermaphrodites were photographed after addition of bleach drop. Top panels: N2 (wild type) worms were still wriggling after 10 sec in bleach, whereas *cat-4(tm773)*, *ptps-1(tm1984)*, and *agmo-1(e3016)* mutants were immobile/dead in a few seconds (<5 sec). Middle panels: N2 worm is dead but intact 30 s after bleaching. Mutants have exploded at multiple sites by 15–30 sec, releasing internal contents. Bottom panels: N2 worm has ruptured, but remains largely intact at 2 min; mutant worms have completely disintegrated and cuticles vanished, leaving eggs and internal debris. (B and C) Cuticle fragility of *cat-4*, *ptps-1* (B), and *agmo-1* (C) mutants. Time (mean ± SD) for cuticle disintegration in mild alkaline bleach, scored for the first major break in the worm cuticle. Groups for each experiment compared with one-factor ANOVA followed by planned pairwise comparisons made with Scheffè’s *F*-test; each experiment showed significant differences among the groups (overall ANOVA, *P* < < 0.0001). (B) Representative experiment with worms from mixed stage cultures (*n* = 15); *cat-4* and *ptps-1* compared to wild type (N2). Asterisks (*) indicate significant differences (*F*-test, *P* < < 0.0001) between each mutant and wild type. Mutants were not significantly different from one another (*P* > 0.05). (C) Two *agmo-1* mutants compared to wild type (N2), synchronized gravid adult hermaphrodites (*n* = 17–27). Both mutants were significantly different from wild type (*P* < <0.0001); double asterisks (**) indicate *e3016* was also significantly different from *e3047* (*P* = 0.002).

We confirmed the role of a predicted PTPS gene (B0041.6; *ptps-1*; Figure S3) by examining the phenotypes of worms with a deletion (*tm1984*) or nonsense mutation (*e3042*) in coding sequence. We found that both *ptps-1* mutants were 5HT-deficient, DA-deficient, and hypersensitive with a fragile cuticle, just like *cat-4* mutants (Figure 3, Figure 4, Table 2). Also like *cat-4* mutants, *ptps-1* mutants can be rescued for 5HT immunoreactivity (5HT-IR) by treating worms with 5-HTP, the immediate precursor to 5HT and product of TPH activity, and rescued for DA (as seen by FIF) by treatment with L-dopa, the immediate precursor to DA and product of TH activity (Figure 3, C, F, I, and L). Both treatments bypass the need for TH and TPH function in synthesis of the neurotransmitters, and also show that serotonergic and dopaminergic neurons are present and appear morphologically normal in *cat-4* and *ptps-1* mutants. We also determined that *ptps-1* mutant male worms, like *cat-4* mutant males (Loer and Kenyon 1993), were defective in the “turning” step of male mating behavior (Figure S4).

The hypersensitivity and cuticle fragility phenotypes of *cat-4* and *ptps-1* mutants are seen most dramatically when worms are placed in a standard “alkaline bleach” solution typically used to kill and dissolve gravid adult worms to isolate the more bleach-resistant eggs. Whereas wild-type worms die and become rigid after many seconds, and take minutes to dissolve, we observed that *cat-4* and *ptps-1* mutant adult worms died in a few seconds or less, ruptured quickly, and their cuticles dissolved completely in <2 min (Figure 4A). To quantify the cuticle fragility phenotype of *cat-4* and *ptps-1* mutants, we tested worms using a mild alkaline bleach treatment. Similar to the effect of standard bleach treatment, *cat-4* and *ptps-1* worms ruptured more quickly: a major break in the cuticle occurred after about 4 min in the solution, compared with 8 min for wild-type worms (Figure 4B). *cat-4* and *ptps-1* mutants showed comparably increased sensitivity to various chemicals and drugs, including the detergent SDS and the anthelminthic levamisole, which in nematodes acts as a cholinergic agonist (Figure S5, A and C). In tests of acute exposure causing worm immobility, the biopterin synthesis mutants were approximately twice as sensitive to SDS compared to wild type, and 5–20 times more sensitive to levamisole.

**Figure 5:**
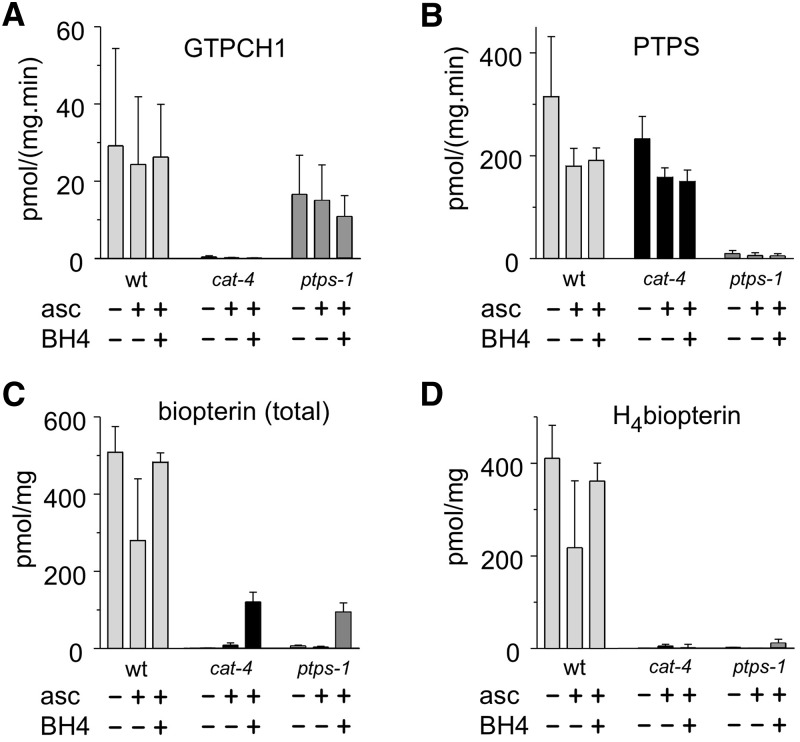
Biopterin synthetic enzyme activity and biopterin levels in wild-type and mutant *C. elegans*. Enzyme activity or biopterin content from worm homogenates from mixed stage cultures. Untreated (-/-), with ascorbate (asc, 5 mM) alone, or asc plus BH4 (200 µM), as indicated below each column. Enzyme activities and BH4 levels from worm homogenates were determined by HPLC with fluorescence detection (see *Materials and Methods*). All levels are expressed relative to protein mass. Mean ± SEM are shown for three to four independent measurements (two to three for *cat-4* mutant). (A) GTPCH1 activity. (B) PTPS activity. (C) Total biopterin derivatives concentration. (D) BH4 concentration. GTPCH1 and PTPS activities were measured in soluble fraction of worm extracts. Total biopterin and BH4 measurements were taken from the same samples. Worm strains: N2, wild type; *cat-4(tm773)*; and *ptps-1(tm1984)*.

To demonstrate that CAT-4 and PTPS-1 are *bona fide* BH4 synthetic enzymes, we measured GTPCH1 and PTPS activity in soluble protein extracts from homogenized worms, as well as total biopterin and BH4 content. *cat-4* null mutants lacked GTPCH1 activity, but displayed PTPS activity, whereas *ptps-1* mutants lacked PTPS activity, but had normal GTPCH1, confirming loss of the predicted functions of these genes (Figure 5, A and B). In addition, very low levels of total biopterin and BH4 were detected in the mutants, compared with levels in wild-type worms (Figure 5, C and D). As expected, BH4 supplementation did not alter enzymatic activities of GTPCH1 or PTPS (Figure 5, A and B), which were determined in homogenates freed from low molecular weight compounds by gel filtration. Rearing worms with exogenous BH4 also failed to increase levels of BH4 in mutants, although total biopterins did increase, indicating some uptake of biopterin (Figure 5, C and D). We observed no rescue of cuticle fragility (Figure S8) or neurotransmitter synthesis in *cat-4* and *ptps-1* mutants supplemented with exogenous biopterins.

### The chemical hypersensitivity and cuticle fragility defects of biopterin mutants result from loss of function in alkylglycerol monooxygenase AGMO

**Figure 6:**
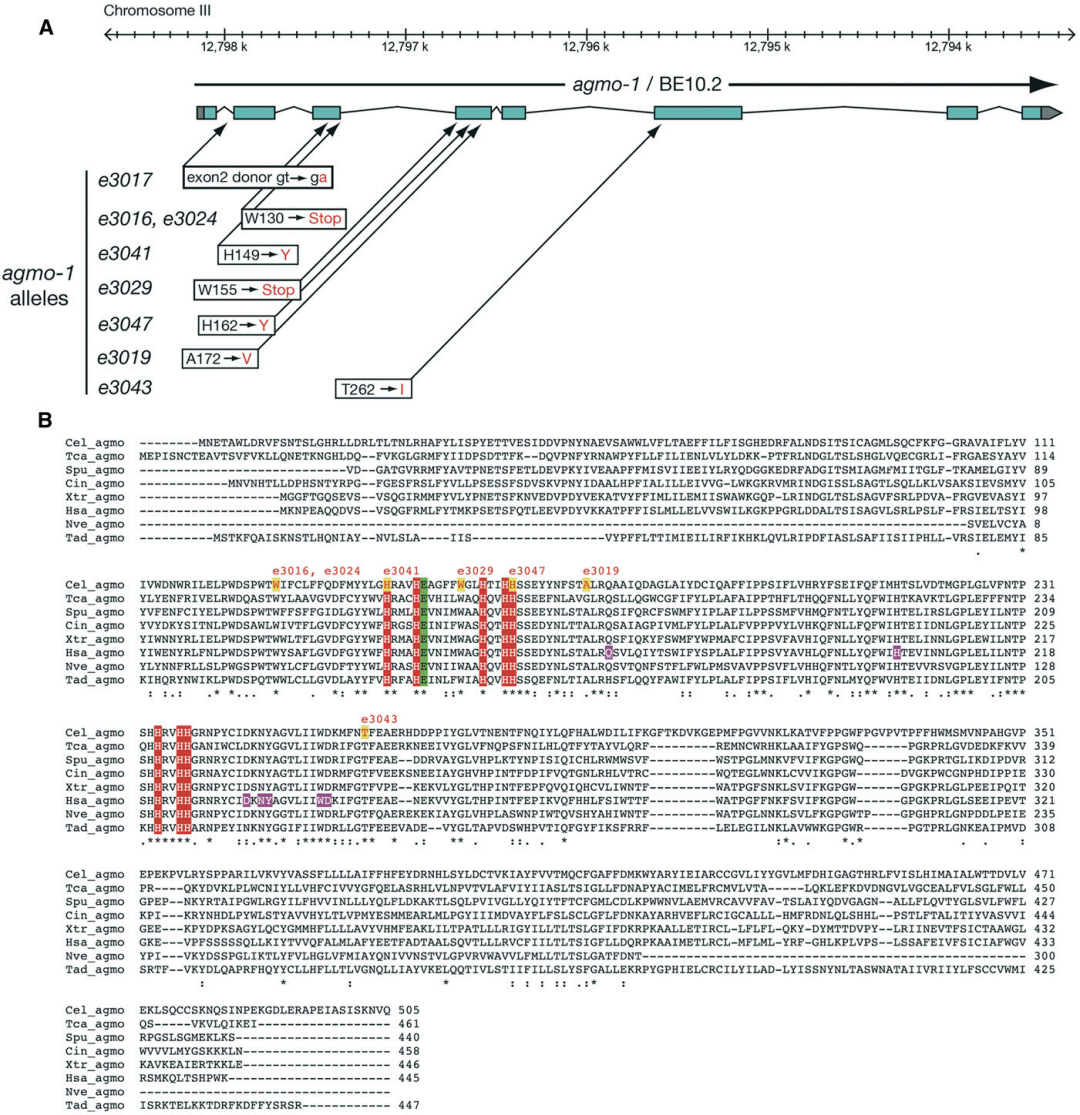
Mutations altering pathogen sensitivity and cuticle strength map to *agmo-1*. (A) Physical map of *agmo-1* region with gene model and location of *agmo-1* mutant alleles (arrows). All alleles were isolated as suppressors of lethality to *Leucobacter Verde1* in *bus* or *srf* mutants. Sequencing of *C. elegans agmo-1* cDNAs confirms the gene model shown (Figure S6), and the predicted protein used for alignments. Image is partly derived from the WormBase genome browser. (B) Alignments of predicted *C. elegans* AGMO protein with human AGMO and putative AGMO proteins from other metazoans. Sequences selected for alignment were best BLASTP matches with both *C. elegans* and human AGMO proteins; *C. elegans* AGMO-1 is ∼38% identical to human AGMO. Most notations are as in Figure 2. Eight 100%-conserved histidines (red) are found in all fatty acid hydroxylases and are required for human AGMO function (Watschinger *et al.* 2010). A conserved Glu (E154) likely required for biopterin binding is indicated in green; additional residues essential for human AGMO activity are in purple (Watschinger *et al.* 2012). Species abbreviations are as in Figure 2, except Tca, *Tribolium castaneum* and Xtr, *Xenopus tropicalis*.

As shown previously, knockout mutants in individual AAAH genes (*pah-1*, *cat-2*, and *tph-1*) are normal with respect to chemical sensitivity and cuticle strength (Table 2); *pah-1* mutants display cuticle defects only in sensitized backgrounds in which the cuticle is already compromised (Calvo *et al.* 2008). These observations suggested that deficiency in function of the newly identified BH4-dependent enzyme AGMO might explain the hypersensitivity and cuticle fragility phenotypes of *cat-4* and *ptps-1* mutants. We therefore examined other mutants with similar phenotypes of cuticle fragility. Genetic screens for *C. elegans* mutants with altered sensitivity to bacterial infections of the cuticle have recovered many mutants with associated chemical hypersensitivity and cuticle fragility phenotypes, presumably due to changes in the cuticle or surface properties of the worm (Gravato-Nobre *et al.* 2005). Among these hypersensitive mutants are those that fail to show the characteristic response to *Microbacterium nematophilum* infection, a swollen rectal epidermis; such mutants have the “Bus” phenotype (**b**acterially **u**n**s**wollen). Interestingly, mutations in certain *bus* genes confer *greater* sensitivity to cuticle infection by bacterial species to which wild-type worms are resistant. The bacterial sensitivity of *bus* mutants can be suppressed by mutation in **su**ppressor of **b**u**s**, or *subs* genes (Hodgkin *et al.* 2013, see *Materials and Methods*). Among *subs* mutants, we found eight independent mutant alleles of *agmo-1*, the *C. elegans* ortholog of the human alkylglycerol monooxygenase (AGMO) gene. Similar to *cat-4* and *ptps-1* mutants, *agmo-1* mutants displayed rapid cuticle disintegration in standard alkaline bleach (Figure 4A). Four *agmo-1* alleles contain premature stop codons, so are likely complete loss of function (Figure 6). Four missense alleles alter amino acids highly conserved among putative AGMO orthologs from other metazoans (Figure 6B, Figure S6), so may abolish or greatly reduce protein function; two of these affect conserved histidines that are among the eight conserved histidines characteristic of fatty acid hydroxylases.

Two new mutant alleles of *cat-4* and one *ptps-1* allele were also isolated via this screen, including a hypomorphic/reduction-of-function allele *cat-4(e3015)*, indicating that complete loss of biopterin synthesis is not required for the Subs phenotype. All *agmo-1* mutants tested were hypersensitive to a variety of chemicals, including alkaline bleach, SDS, and levamisole, comparable to *cat-4* and *ptps-1* mutants (Figure 4C, Figure S5, B and D). As expected, *agmo-1* mutants displayed normal levels of DA and 5HT in neurons (Figure S7), indicating the independence of the neuronal and epidermal roles of BH4.

### The BH4 regeneration cycle maintains biogenic amine levels under conditions of limiting BH4

**Figure 7:**
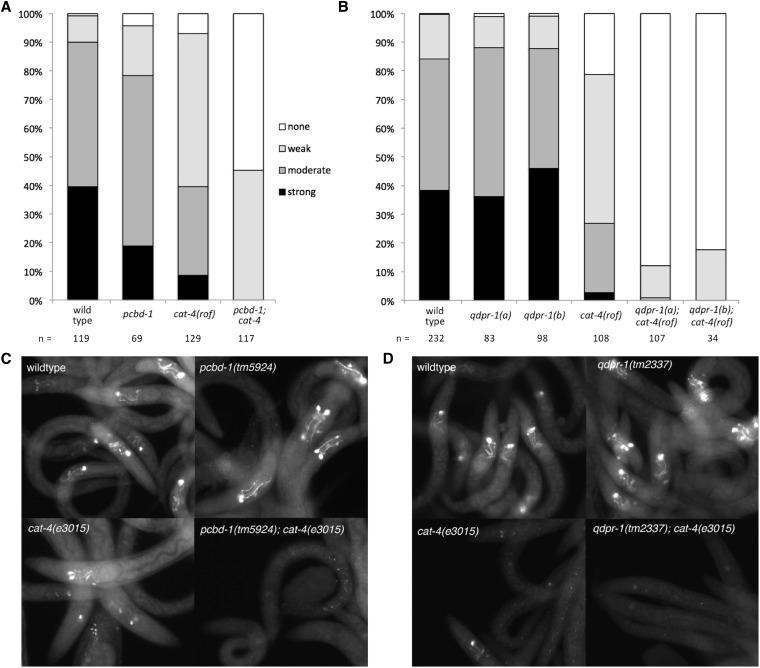
5HT synthesis is dependent on the biopterin regeneration pathway. Serotonin immunoreactivity (5HT-IR) in NSM somas and neurites of young (L1–L2) worms. Mixed populations of wild type (N2), single, and double mutant worms were scored by serotonin antibody staining. Staining definitions are as follows: strong, somas bright (saturated staining, no internal features apparent) and neurites bright; medium, somas not saturated, may show some internal structure (*i.e.*, a nucleus may be seen), neurites present; weak/faint, somas visible (may be just above background), neurites absent or very faint; and none, no stained structures apparent. (A) *pcbd-1(tm5924)* mutants display at most a mild reduction in 5HT-IR. Double mutant *pcbd-1*; *cat-4(rof)* worms display almost no 5HT-IR; *cat-4(rof)* = reduction of function allele = *e3015*. (B) Loss of 5HT-IR in *qdpr-1* mutant worms. Wild type (N2), *qdpr-1*, *cat-4*, and double mutant *qdpr-1*; *cat-4* worms scored as described in A. *qdpr-1(a)* = *tm2337*; *qdpr-1(b)* = *tm2373*. (C) Examples of staining in mutants including *pcbd-1(tm5924)*. (D) Examples of staining in mutants including *qdpr-1(tm2337)*. All images in C and D were taken using the same exposure.

We further tested putative knockout alleles of the worm biopterin regeneration enzyme genes *qdpr-1* and *pcbd-1*. These mutants showed no obvious morphological or behavioral phenotypes, and initially appeared wild type for 5HT, DA, and bleach sensitivity (Figure 3, Table 2). Because loss of BH4 regeneration in the context of normal *de novo* BH4 synthesis may not reduce BH4 levels sufficiently to cause an obvious phenotype, we also tested *qdpr-1* and *pcbd-1* mutants in combination with the *cat-4* reduction-of-function allele, *cat-4(e3015)*. Reduction of neurotransmitters in *cat-4(e3015)* worms was most apparent in young larvae; *e3015* adult worms were nearly wild type, suggesting that functional BH4 accumulates over the life of the worm (Figure S9). We found that both 5HT and DA were very strongly reduced in double mutants with *qdpr-1* and *pcbd-1* in comparison to the single mutant *cat-4(e3015)*. The double mutants also showed the strongest reduction in young larvae (Figure 7), although differences were also apparent among adults (Figure S10). For example, although 5HT-IR was absent in only 7–20% of L1–L2 *cat-4* worms, 55% of *pcbd-1(tm5924)*; *cat-4(e3015)* worms lacked 5HT staining (Figure 7, A and C). Similarly, 82% of *qdpr-1(tm2373)*; *cat-4(e3015)* and 88% of *qdpr-1(tm2337)*; *cat-4(e3015)* lacked 5HT staining (Figure 7, B and D). Similar results were obtained using FIF staining to detect DA (Figure S11). These results demonstrate an important role for biopterin regeneration under conditions in which biopterin levels may be limiting for neurotransmitter synthesis.

### BH4 synthesis genes are expressed in biogenic amine neurons and in the epidermis

**Figure 8:**
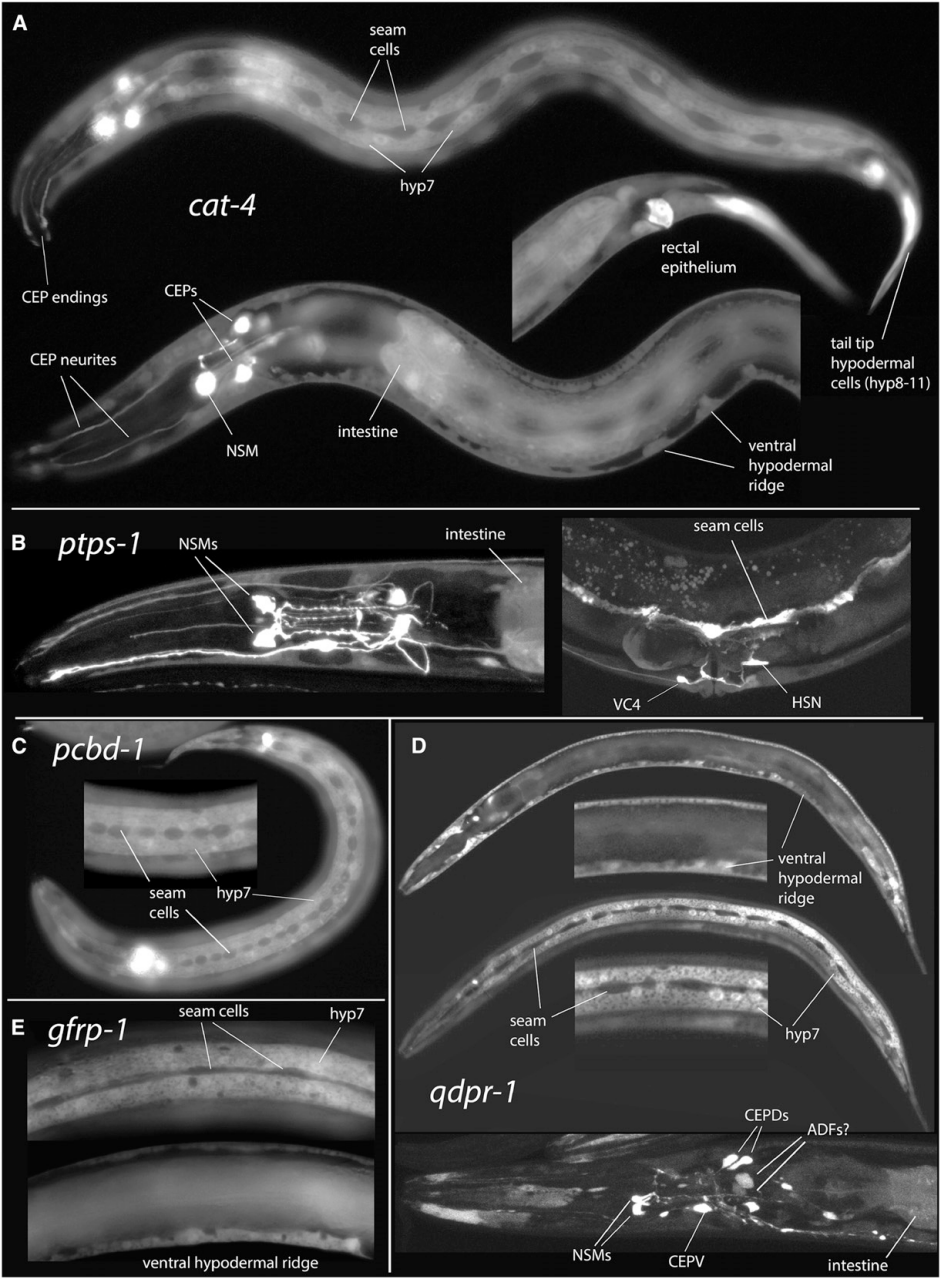
Biopterin synthetic genes are expressed in biogenic amine neurons and in the epidermis. (A) Expression of *cat-4* GFP reporters in larval stage 2 (L2) worms, construct with ∼2.7 kb upstream sequence (strain CZ9719). Top: Superficial focal plane showing epidermal expression, especially in the hyp7 syncytium. Seam cells have undergone doubling division and can be seen along the lateral side as darker regions among the brightly staining hyp7 cell. Dendritic endings of CEP neurons can be seen at the tip of the “nose.” Middle: Medial focal plane showing anal cells, strongly expressing tail epidermal cells and posterior intestinal cells expressing GFP. Bottom: Medial focal plane showing epidermal expression in the body and head. In these transgenics, expression in the head is seen in hyp6, but not the more anterior hyp5. NSM and CEP neuron somas are seen in the head, plus some neuronal processes (especially CEP processes). A few other neuronal somas stain less brightly. The anterior intestine also shows GFP expression, as do some rectal epithelial cells (likely B and Y cells). (B) *ptps-1*-GFP reporters. Left: head showing NSM expression plus other neurons and nonneuronal cells, including anterior intestine (dorsal–ventral view, maximum intensity projection) (MIP); strain OH11619, described by Zhang *et al.* (2014). Right: vulval region showing expression in neurons VC4, 5 (weak) and HSN, plus lateral seam cells (lateral view, MIP); strain CZ18321. (C) *pcbd-1* reporter in larva showing expression in epidermal syncytium hyp7, tail and head epidermis, but not in seam cells; strain CZ19212. Out of focus: pharyngeal muscle and rectal epithelium expression. Inset: enlargement of midbody region. (D) *qdpr-1* reporters. Top images: single optical sections from larva showing broad epidermal expression, but little expression in neurons; strain CZ19213. Insets: enlargements of midbody regions. Bottom image: Head showing expression in identified 5HT and DA neurons: NSMs, ADFs, CEPs, and other cells, with reduced epidermal expression; strain CZ19215 (lateral view, MIP). (E) *gfrp-1* reporter showing expression in epidermal syncytium hyp7 (top: superficial focal plane) and other epidermal cells (bottom: central focal plane). (A–E) Anterior is to the left in all worms. (A, C, and E) Standard epifluorescence. (B and D) Laser scanning confocal imaging; (B and D, bottom) images are maximal intensity projection of *Z*-stack; and (D, top and middle) single confocal image planes.

Consistent with the known role of GTPCH1 in synthesizing BH4, required for the function of AAAHs, GFP reporter constructs show *cat-4* expression in identified serotonergic and dopaminergic neurons (where TPH and TH are expressed, respectively), and in the epidermis (where both PAH and AGMO are expressed, see below). We examined *cat-4* reporter constructs from three different sources, including some previously described by others (see *Materials and Methods*; Table S1 and Table S2). Overall, in larval and adult worms, we observed strong expression in serotonergic and dopaminergic neurons, in most of the epidermis—especially the large epidermal syncytium (hyp7)—and more weakly in some intestinal cells (Figure 8A). Given the phenotypes of *ptps-1* mutants, and the predicted function of the gene, we expected that the gene’s expression would likely match that of *cat-4*/GTPCH1. We made and examined three independent *ptps-1*::GFP reporter transgenics (translational fusion in the final coding exon plus 2.0 kb of sequence upstream of the predicted ATG), as well as a *ptps-1* reporter transgene (∼2.6 kb upstream sequence) described elsewhere (Zhang *et al.* 2014). Transgenics with the shorter upstream sequence resembled the *cat-4* expression pattern, with expression in some epidermal cells, and a few serotonergic neurons (Figure 8B). The *ptps-1* reporters, however, were also observed in additional cells, and were not expressed in other epidermal cells or in all 5HT and DA neurons, suggesting these reporters may lack important positive or negative regulatory elements.

We made and examined reporter transgenics for the *pcbd-1*, *qdpr-1*, and *gfrp-1* genes, and also examined some described by others (Zhang *et al.* 2014). All were expressed in the epidermis, similar to that observed in *cat-4* transgenics (Figure 8, C–E), but were not highly expressed in 5HT and DA neurons. The epidermal expression, when observed, was similar in localization, intensity, and developmental timing in larvae. All of these transgenics also showed some expression of varying intensity in other cells (nonepidermal cells, and non-5HT and non-DA neurons in the head and body). When we examined one *qdpr-1* transgenic with 4.9-kb genomic sequence (full-length translational fusion with ∼4.0 kb upstream), we observed expression also in several known 5HT and DA neurons (Figure 8D, bottom panel). This transgenic showed some reduction in epidermal expression. With the exception of the larger *qdpr-1* construct, the other previously described transgenics (Zhang *et al.* 2014) were expressed similarly to those reported here.

### *agmo-1* is expressed throughout the epidermis beginning in embryogenesis

**Figure 9:**
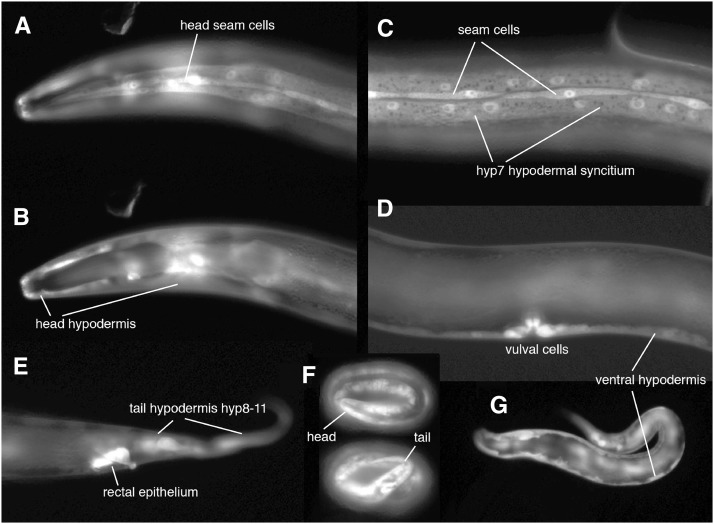
*agmo-1* is expressed in the *C. elegans* epidermis. (A–G) Expression of *agmo-1*::GFP reporter fusions in the epidermis; all worms at same magnification, standard epifluorescence. (A–E) Young adult hermaphrodites; anterior to the left. (A) Head, surface focal plane showing expression in epidermal syncytial cells and seam. (B) Head, mid-depth focal plane showing expression in all head epidermal cells (same head as A). (C) Lateral body surface showing expression in epidermal syncytium hyp7 and lateral seam cells. (D) Mid-depth focal plan showing expression in vulval cells and ventral epidermal ridge. (E) Tail showing strong expression in rectal epithelial cells and other tail epidermis. (F) Two 3-fold-stage embryos with very strong expression in epidermis. (G) Young larva, mid-depth focal plane, showing expression like that seen in adult (and all other larval stages).

We examined *agmo-1*::GFP reporter fusion transgenics and found that an *agmo-1* transgene with ∼2300 bp of upstream sequence was expressed in the epidermis similarly, but not identical to *cat-4* reporter constructs (Figure 9). *agmo-1*::GFP reporter constructs were expressed more broadly in the epidermis than were *cat-4* or *pah-1* reporter constructs (Figure 9, A–E, Figure S12). We typically saw expression in all epidermal syncytia and in seam cells at all stages, beginning in the embryo. Based on GFP intensity, *agmo-1* is particularly highly expressed in late stage embryos when the L1 cuticle is being constructed prior to hatching. *cat-4* and *pah-1* reporters are also expressed in embryos, although expression appears earlier in embryogenesis (Figure S13).

## Discussion

We have characterized the genetic basis of biopterin/BH4 biosynthesis and function in *C. elegans*. We have demonstrated that the mutationally identified *cat-4* gene encodes GTPCH1, and that mutations in a PTPS-encoding gene cause phenotypes identical to those of *cat-4* mutants. *cat-4* and *ptps-1* mutants lack their respective enzymatic activities, have greatly decreased BH4 content, and have phenotypes consistent with their predicted roles in BH4 synthesis, including loss of the neurotransmitters 5HT and DA. Biopterin synthesis mutants were not rescued by supplemental BH4 or sepiapterin. Although some exogenous biopterin was taken up, we could only detect the inactive form 7,8-dihydrobiopterin (BH2). In mammalian cell culture and in mice, BH4 is taken up as BH2 and then reduced to BH4 by DHFR (Hasegawa *et al.* 2005). In *C. elegans*, we hypothesize that exogenous BH4 is similarly taken up as BH2, but for unknown reasons is not subsequently reduced to BH4 by the *C. elegans* DHFR. In any case, the lack of functional rescue of the BH4-deficient mutants by exogenous biopterins is consistent with the general requirement for biopterin synthesis in cells and tissues that use BH4-dependent enzymes (Thöny *et al.* 2000).

Our observations, using 5HT and DA synthesis as a proxy for biopterin levels, demonstrate that *pcbd-1* and *qdpr-1* genes, predicted to encode BH4 regeneration enzymes, function to maintain biopterin needed for neurotransmitter synthesis. The biopterin regeneration mutants showed clear effects on neurotransmitter synthesis when combined with a reduction-of-function *cat-4* mutation. Because of the inherent variability of both techniques—immunofluorescence (for 5HT) and FIF (for DA)—possible slight reductions in staining observed in the *pcbd-1* or *qdpr-1* single mutants could not be definitively assessed. Thus in *C. elegans*, function of these BH4 regeneration genes may be specifically revealed when BH4 biosynthesis is impaired.

Besides lacking 5HT and DA, *cat-4* and *ptps-1* mutants display increased sensitivity to many chemicals, and a less sturdy cuticle. The cuticle defects of *cat-4* have been a long-standing conundrum, as they are not seen in mutants in known BH4-dependent AAAH enzymes (*pah-1*, *cat-2*, and *tph-1*). Solution of this puzzle awaited the cloning of the last remaining BH4-dependent enzyme, alkylglycerol monooxygenase/AGMO (Watschinger *et al.* 2010), allowing identification of the *C. elegans* AGMO ortholog. Mutations in *agmo-1* were predicted to cause cuticle fragility and hypersensitivity, and such mutations were indeed subsequently found in screens for altered resistance to a worm bacterial pathogen that infects animals via the cuticle (Hodgkin *et al.* 2013). Consistent with a role in establishing outer surface properties of the worm, *agmo-1* reporters are expressed throughout the epidermis, which secretes the cuticle, a complex extracellular matrix (Chisholm and Xu 2012). The epidermis is also most likely the cellular origin of the epicuticle, a poorly understood extracellular lipid-rich layer that has been hypothesized to function as a permeability barrier and in pathogen defense (Chisholm and Xu 2012). Identification of AGMO as required for cuticle integrity further supports the idea that lipid metabolism is critical for surface properties of nematodes.

Our analysis of biopterin-related gene expression in *C. elegans* by reporter fusion transgenics is generally consistent with BH4 synthesis being required cell autonomously for AAAH function in 5HT and DA neurons and for AGMO function in the epidermis. Not all our reporter transgenics, however, were expressed in patterns expected based on mutant phenotypes of the genes examined. Transgenes reported here are mostly transcriptional fusions and may lack positive or negative regulatory elements. Further work, using rescuing transgenes or under endogenous or tissue-specific control, will be required to fully define the cellular requirements for BH4 synthesis.

Our studies reveal the first *in vivo* biological role for the BH4-dependent lipid metabolic enzyme AGMO, which is the only enzyme known to degrade the ether lipid bond in alkylglycerols and alkylglycerol-lyso-phospholipids (Watschinger and Werner 2013). The precise biochemical role of AGMO in the cuticle and permeability barrier in the worm remains to be established. We do not know whether alkyl ether lipid metabolism in *C. elegans* serves primarily an anabolic or catabolic function: *agmo-1* mutant phenotypes could result from failure to synthesize a needed product or by accumulation of toxic intermediates. Failure of the AGMO reaction might lead to accumulation of ether lipids, somehow destabilizing the lipid-rich epicuticle. Alternatively, AGMO-1 substrates might alter cuticle development via signaling pathways, analogous to the effects of antitumor ether lipids on mammalian tumor cells (Arthur and Bittman 1998).

Other mutants with altered pathogen sensitivity in *C. elegans* display chemical hypersensitivity and cuticle fragility. The *bus* mutants are partially resistant to bacterial rectal infection by certain worm pathogens, apparently due to alterations in cuticle surface features (Gravato-Nobre *et al.* 2005). Some of these mutants are bleach hypersensitive, dying and rupturing more quickly than N2 wild type. Several *bus* genes have now been identified, and some encode lipid metabolic enzymes expressed in cells that overlap with *cat-4* expression, suggesting a pathway association with *agmo-1*. For example, *bus-18* (aka *acl-10*) encodes a lysocardiolipin acyltransferase expressed in the epidermis (Gravato-Nobre and Hodgkin 2008; Imae *et al.* 2010). However, in general, the cuticle defects of such *bus* mutants are more severe than those of biopterin synthesis or *agmo-1* mutants and extend to outer cuticle sloughing during handling, rupture at the vulva, and “skiddy” locomotion. These more severe cuticle defects suggest additional, widespread roles for lipid metabolism in the cuticle.

In conclusion, our systematic analysis of BH4 function in *C. elegans* confirms its essential role as a cofactor for TH and TPH in neurotransmitter synthesis in neurons. In addition to this well-established function, we elucidate here a new role for BH4 as a cofactor for AGMO in epidermal cells. Our studies reveal an unexpected *in vivo* role for AGMO in supporting cuticle stability and sensitivity to bacterial infection, presumably via ether lipid metabolism. These observations raise the question of whether AGMO might play comparable roles in epidermal function in other animals or in humans. The presence of a complete enzymatic pathway for *de novo* synthesis of BH4 has been established in the human epidermis (Chavan *et al.* 2006), but at present no mutations in mammalian or human AGMO have been reported, nor have severe skin defects been described in patients deficient in BH4 synthesis. Nevertheless, we note that the products of AGMO catalysis are converted to fatty acids by fatty aldehyde dehydrogenase (FALDH), and that FALDH deficiency in humans results in a combination of skin permeability barrier defects, ichthyosis, and neurological disease known as Sjögren-Larsson syndrome (Rizzo 2011). It would be interesting to explore whether AGMO contributes to lipid-based permeability barrier function in other organisms or in humans.

## Supplementary Material

Supporting Information
