# Supplementary material for: Cuticle Integrity and Biogenic Amine Synthesis in Caenorhabditis elegans Require the Cofactor Tetrahydrobiopterin (BH4)
Source: Genetics. 2015 Mar 24;200(1):237–53. doi: 10.1534/genetics.114.174110 (PMC4423366; doi:10.1534/genetics.114.174110)
Supplement: Supporting Information [file supp_200_1_237__index.html]

Cuticle Integrity and Biogenic Amine Synthesis in Caenorhabditis elegans Require the Cofactor Tetrahydrobiopterin (BH4) — Supporting Information 

# Cuticle Integrity and Biogenic Amine Synthesis in *Caenorhabditis elegans* Require the Cofactor Tetrahydrobiopterin (BH4)

## Supporting Information

**Supporting Information**

- Supporting Information - Figures S1-S13, File S1, Tables S1-S2, and References (PDF, 4 MB)
- Figure S1 - *cat-4* cDNA sequence encodes a 223 amino acid protein. (PDF, 440 KB)
- Figure S2 - Rescue of neurotransmitter synthesis in *cat-4* mutants by genomic F32G8.6-containing sequence. (PDF, 314 KB)
- Figure S3 - The *ptps-1*/ B0041.6 gene encodes 6-Pyruvoyl Tetrahydropterin Synthase (PTPS). (PDF, 493 KB)
- Figure S4 - Male turning behavior is defective in *ptps-1* mutants. (PDF, 143 KB)
- Figure S5 - Similar hypersensitivity phenotypes of *cat-4, ptps-1* and *agmo-1* mutants. (PDF, 199 KB)
- Figure S6 - An *agmo-1* cDNA encodes a 505 amino acid protein. (PDF, 2 MB)
- Figure S7 - *agmo-1* mutants have normal 5HT and DA. (PDF, 164 KB)
- Figure S8 - Exogenous biopterins do not rescue cuticle fragility in BH4-deficient mutants. (PDF, 204 KB)
- Figure S9 - *cat-4* reduction-of-function mutants accumulate 5HT during larval development. (PDF, 244 KB)
- Figure S10 - Biopterin regeneration genes *pcbd-1* and *qdpr-1* function in 5HT synthesis in adult worms. (PDF, 372 KB)
- Figure S11 - Biopterin regeneration genes *pcbd-1* and *qdpr-1* function in DA synthesis. (PDF, 155 KB)
- Figure S12 - Expression of *cat-4* and *pah-1* GFP reporters. (PDF, 361 KB)
- Figure S13 - Expression of *cat-4, pah-1* and *ptps-1* reporter constructs in embryos. (PDF, 472 KB)
- File S1 - Supporting Materials and Methods (PDF, 117 KB)
- Table S1 - Biopterin-related GFP reporter gene transgenics (this work) - primers and characteristics. (PDF, 89 KB)
- Table S2 - Biopterin-related GFP reporter gene transgenics from other sources. (PDF, 74 KB)
