# Supplementary material for: Cuticle Integrity and Biogenic Amine Synthesis in Caenorhabditis elegans Require the Cofactor Tetrahydrobiopterin (BH4)
Source: Genetics. 2015 Mar 24;200(1):237–53. doi: 10.1534/genetics.114.174110 (PMC4423366; doi:10.1534/genetics.114.174110)
Supplement: Supporting Information [file supp_114.174110_174110SI.pdf]

```
    SL1 spliced leader
   ggtttaattacccaagtttgaggctttacccactctatcaacatcataatatcaaa

atgtccagaattgagaacgaaagcggatttttgtcatctgacgccgcaagtgttggatcc
 M  S  R  I  E  N  E  S  G  F  L  S  S  D  A  A  S  V  G  S

gaagacgataaagttgagatgaagaaaagaaacggaacgattccaaaggaagatcatttg
 E  D  D  K  V  E  M  K  K  R  N  G  T  I  P  K  E  D  H  L

                                             ↓
aaatcaatgtgcaatgcctatcagagcatcattcaacatgtcggagaagacatcaatcgt
 K  S  M  C  N  A  Y  Q  S  I  I  Q  H  V  G  E  D  I  N  R

cagggacttctgaaaactccagaacgtgctgccaaagcaatgatggcattcacaaaggga
 Q  G  L  L  K  T  P  E  R  A  A  K  A  M  M  A  F  T  K  G

                   ↓
tacgatgatcaacttgatgagctcctcaacgaggcagtattcgacgaggatcacgatgag
 Y  D  D  Q  L  D  E  L  L  N  E  A  V  F  D  E  D  H  D  E

atggttattgtgaaagatattgaaatgttctccctttgtgagcatcatttagttccattt
 M  V  I  V  K  D  I  E  M  F  S  L  C  E  H  H  L  V  P  F

atgggaaaagttcatattggatacattccaaacaagaaggttcttggtctgtccaagttg
 M  G  K  V  H  I  G  Y  I  P  N  K  K  V  L  G  L  S  K  L

                                      ↓
gcaagaatagtcgagatgttcagcagaagacttcaagtccaagaacgtcttacaaagcaa
 A  R  I  V  E  M  F  S  R  R  L  Q  V  Q  E  R  L  T  K  Q

attgccaccgcaatggttcaagctgtacaaccatccggagttgcagttgttattgaagct
 I  A  T  A  M  V  Q  A  V  Q  P  S  G  V  A  V  V  I  E  A

agtcacatgtgtatggtaatgagaggagttcaaaagatcaatgcttccaccacaacatcc
 S  H  M  C  M  V  M  R  G  V  Q  K  I  N  A  S  T  T  T  S

tgcatgttgggagtattccgtgacgatccaaagactcgcgaagaattccttaatcttatc
 C  M  L  G  V  F  R  D  D  P  K  T  R  E  E  F  L  N  L  I

aacaaacgctaaaaattctagtcatttcctttttttacttgtttttttgggttttaactgt
 N  K  R

tactaacttttcataactttccatgtgaatctactaacatcttatgcttcagctttttaa

attcatacaacatgttcattgcctgg
```

Figure S1 *cat-4* cDNA sequence encodes a 223 amino acid protein. We isolated, cloned and sequenced *cat-4* cDNAs by RT-PCR from mixed stage *C. elegans him-5* worms as previously described (Hare and Loer 2004); cDNA clones were also obtained from the ORFeome project (Reboul et al. 2001). For *cat-4*, twelve independent clones from pooled ORFeome DNA were partially sequenced; 11/12 were identical F32G8.6 cDNAs, 1/12 was a different non-F32G8.6 sequence. We found that *cat-4* message is trans-spliced to SL1 (underlined at the 5' end). All cDNA clones we examined and those found in databases from various transcriptomics projects showed the same pattern of splicing, indicating there is likely a single type of mRNA and therefore a single isoform of the worm GTPCH1 protein with 223 amino acids. Introns are located in genomic sequence following the base indicated by a red arrow. Genbank accession numbers associated with these *cat-4* cDNA clones are KP290890-KP290893.

We also used RT-PCR to isolate *cat-4* cDNAs from *C. briggsae* which were very similar to those from *C. elegans*. RT-PCR from the diplogastrid nematode *Pristionchus pacificus* yielded 2 different cDNAs: a 9 exon transcript encoding a 270 aa protein, and an 8 exon transcript encoding a 245 aa protein. The longer cDNA matches some gene predictions, whereas the shorter cDNA encodes a more typical GTPCH1 protein.

It is notable that GTPCH1 transcripts are among the most abundant transcripts in some nematodes. For example, among SL1-spliced ESTs from a mixed stage library from the plant parasitic nematode *Pratylenchus penetrans,* 6% were from GTPCH1 (Mitreva et al. 2004). In several mammalian gastrointestinal parasites (e.g., *Ostertagia ostertagia*, *Haemonchus contortus*, *Teladorsagia circumcincta),* a significant fraction of transcripts – up to 30% – from infective L3 stage larvae encoded GTPCH1 (Hoekstra et al. 2000; Moore et al. 2000; Nisbet et al. 2008).

We also examined worms with the *cat-4* missense allele *gk245686* from the Million Mutation Project (Thompson et al. 2013). In this mutant, there is a relatively conservative change (M185I), although the alteration is in an amino acid that is 100% conserved in metazoans, and likely within the active site (see Fig 1). Perhaps surprisingly, worms with this mutation had no obvious neurotransmitter deficiency or bleach hypersensitivity, although the change is conservative.

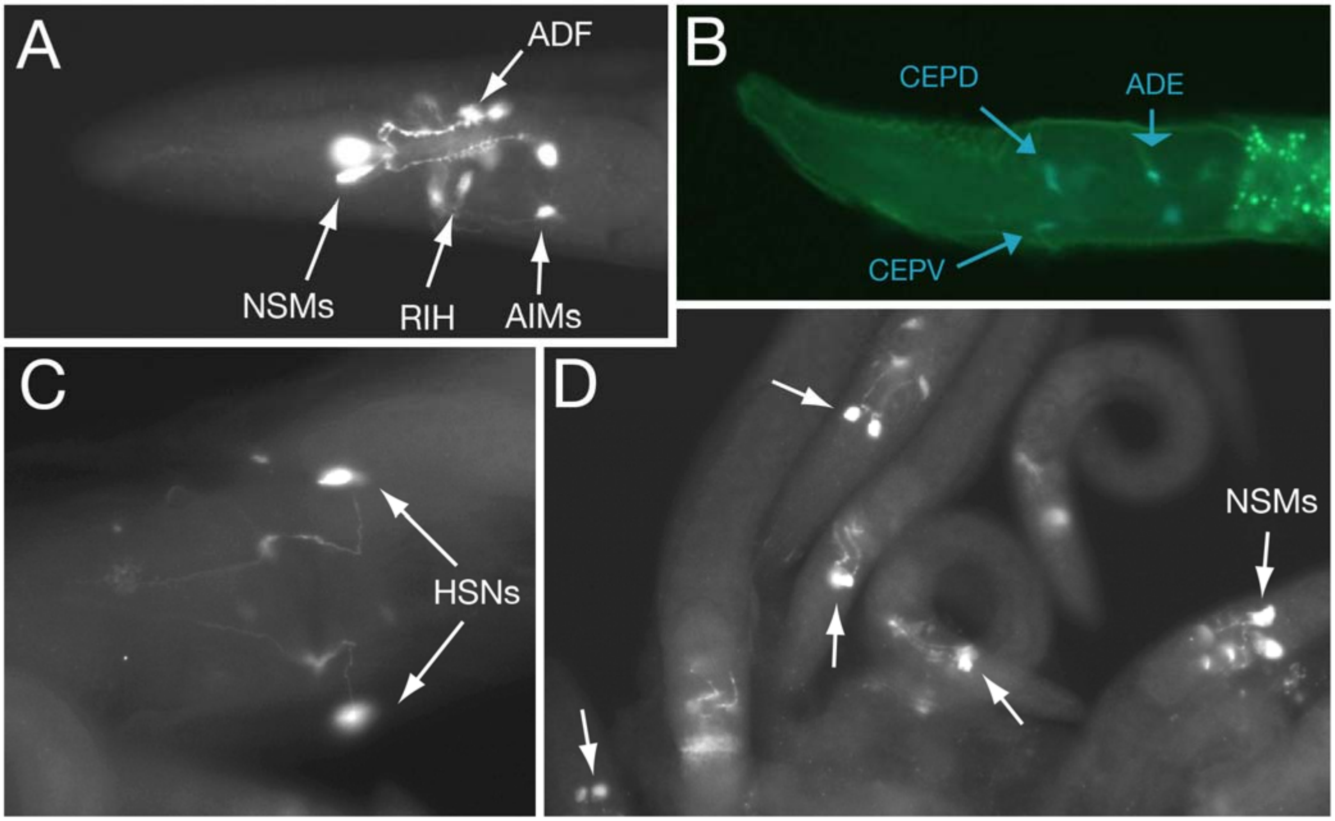

Figure S2 Rescue of neurotransmitter synthesis in *cat-4* mutants by genomic F32G8.6-containing sequence. Anti-5HT immunofluorescence (A, C, D) or FIF (B) of adult and larval *cat-4(tm773)* worms rescued with F32G8.6-containing plasmid (from Baker et al. 2012). (A) Head of adult worm showing presence of all normal serotonergic neurons; somas indicated with arrows. NSM neurites are apparent. (B) FIF of larval worm head showing presence of normal complement of DA neurons. (C) Adult hermaphrodite serotonergic HSN egg-laying neurons innervating vulval region and extending neurites anteriorly to the head. (D) Many rescued (5HT positive) larvae. NSM neuron somas indicated with arrow.

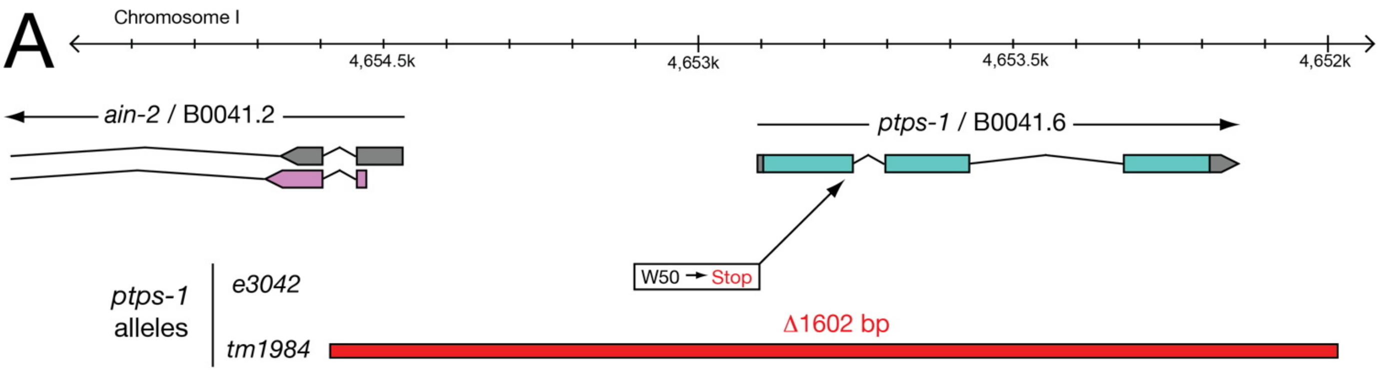

B

```
                                                                                                                          e3042
Cel_PTPS  ------------------------------MFRMPIVTMERVDSFSAAHRLHSEKLSDAENKETFGKCNNSNGHGHNYVWKVKLRGEVDPTSGMVYDLAKLKKEMS-LVLDTVDHRNL  87
Dme_PTPS  ------------------------------MSQQPVAFLTRRETFSACHRLHSPQLSDAENLEVFGKCNNFHGHGHNYTVEITVRGPIDRRTGMVLNITELKEAIETVIMKRLDHKNL  88
Spu_PTPS  ------------------------------MSDDFDIEAL-NGGFP-----MSPELSDDENIKLYDKCNNPNGHGHNYKVELTLRGKVYPVTGMVMNISDLKAYMKNGFLDLMDHKNL  82
Cin_PTPS  ----------------------------------MKVFLTRRLTLCASHRLHAPQLSDEDNLRIYGKCNPPNGHGHNYEVKVTVYGEVDPVTGMVINAVDLKEYMQKAIMEPMDHKCV  84
Dre_PTPS  ------------------------------MSER-VAFITRVCSFSACHRLHSKCLSDEENKRTFGKCNNPNGHGHNYTVEVTVRGKIDKNTGMVMNLTDLKEFIEEAVMKPLDHKNL  87
Xla_PTPS  SSLCSTDSTESSGLAAVNILNMQNCDLITSHPEPRIIQISRSLSFSASHRLHSNSLSEEENKRIFGKCNNPNGHGHNYKAIVTVRGKIDPITGMVMNLTDLKRYMEECITVPLDHKNV 118
Hsa_PTPS  ------MSTEGGGRRCQ-------------------AQVSRRISFSASHRLYSKFLSDEENLKLFGKCNNPNGHGHNYKVVVTVHGEIDPATGMVMNLADLKKYMEEAIMQPLDHKNL  93
Nve_PTPS  ----------------------------MACNKRPFVYITRSETFSACHRLHSTKLTDQQNIELFGKCNNKNGHGHNYKVEVTVASTVDEDTGMVMNLVALKGKIEEVLS-ELDHKNL  89
Tad_PTPS  ----------------------------MAERKR-IGYIKRIESFSAAHRLHSPFFTDEENRALYGKCNHENGHGHNYKLEVTLKGEIDLKTGMVMNIADLKVIINEVIMKQLDHKNL  89
                                          : *   ::* ***:: :::: :*   :.***  :******   :.: . :   :*** :   **  :.  .   :**: :

Cel_PTPS  DKDVEFFKTTVSTSENVAIYMFEKLKSVMSN-PSVLYKVTIEETPKNIFTYKGC--------------------------- 140
Dme_PTPS  DKDVEYFANTPSTTENLAVYIWDNIRLQLKK-PELLYEVKIHETPKNIISYRGPYPLNGIYNPINKRIAHDSCTNISSDSD 168
Spu_PTPS  DKDVNYFRTNVSTVENIAIFIWDSMAKCLPD-PSLLYEVKVHETDKNIAVYRGE--------------------------- 135
Cin_PTPS  DKDIDYFKDKPSTAENIAIYIWQQLEPYMKNEKCSLYKVQLCETENIVTEYMGEEG------------------------- 140
Dre_PTPS  DLDVPYFADVVSTTENLSVFIWDGLQKLLP--HDSLYEIKVYETAKNIVIYRGE--------------------------- 139
Xla_PTPS  DKDVPYFRNVVSTVENITVFIWEELQKRLP--AGLLYEVCVHETDSNSAVYRGE--------------------------- 170
Hsa_PTPS  DMDVPYFADVVSTTENVAVYIWDNLQKVLP--VGVLYKVKVYETDNNIVVYKGE--------------------------- 145
Nve_PTPS  DLDVEYFQTVVSTAENIAVYIYNQLNRGLPD-G-ILYEVRVHETDKNVAFYRGQ-----IKETLN---------------- 147
Tad_PTPS  DKDVPYFANYPSTAENIAVYIWDCLIEHLP----ILYEVLLYETDKNVAIYRGE--------TNN---------------- 142
          * *: :*    ** **:::::::: :   :      **:: : ** .    * *
```

Figure S3 The *ptps-1/* B0041.6 gene encodes 6-Pyruvoyl Tetrahydropterin Synthase (PTPS). (A) Physical map of *ptps-1* region with mutant alleles and gene model. Nature of *ptps-1* alleles is shown below and approximate locations indicated with arrow (point mutation) or red bar (deletion). Various EST, OST, and transcriptome sequencing support the gene model shown, and the predicted protein sequence. Image partly derived from WormBase genome browser editable SVG. (B) Alignments of *C. elegans* PTPS-1 predicted protein with PTPS proteins from other metazoans. Asterisks below alignment show 100% conserved amino acids, colon indicates conserved highly similar aa's, period indicates conserved weakly similar aa's. Location of *ptps-1* mutant allele *(e3042)* marked with red letter. Species abbreviations: Cel – *C. elegans*, Dme – *Drosophila melanogaster,* Spu – *Strongylocentrotus purpuratus*, Cin – *Ciona intestinalis*, Dre – *Danio rerio*, Xla – *Xenopus laevis*, Hsa – *Homo sapiens*, Nve – *Nematostella vectensis*, Tad – *Trichoplax adherens.*

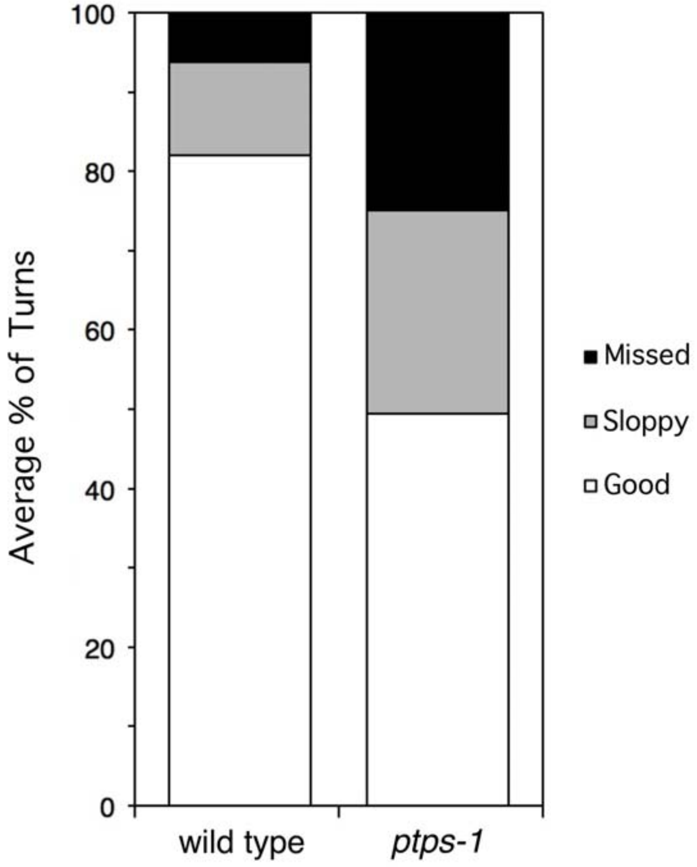

Figure S4 Male turning behavior is defective in *ptps-1* mutants. Columns show average percentage of each type of turn (good, sloppy, missed; definition of turn types from (Loer and Kenyon 1993) for each individual male, each observed for 5 min (wild type, n = 10 *him-5(e1490)* males; n = 12 *ptps-1(tm1984); him-5(e1490)* males).

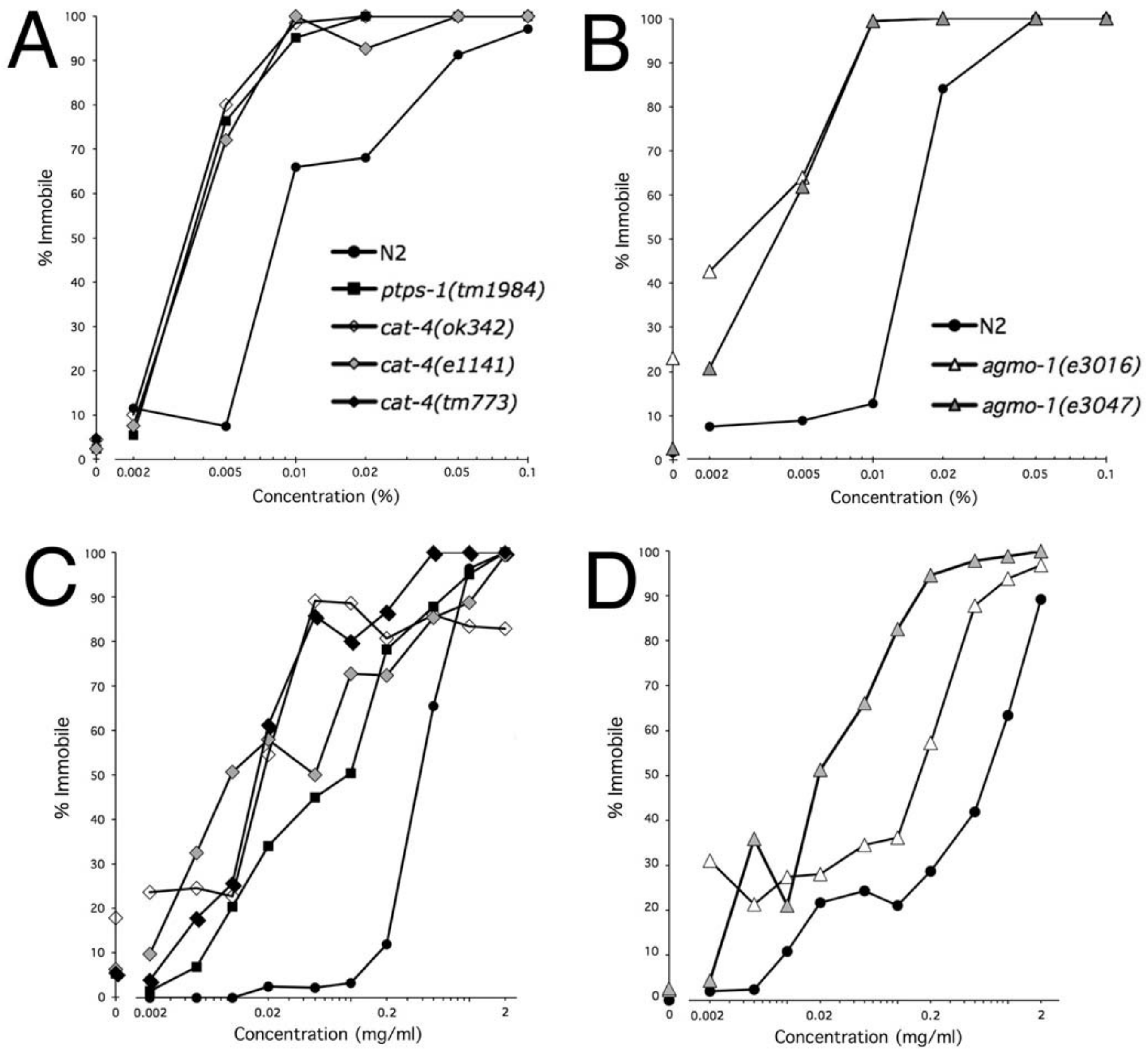

Figure S5 Similar hypersensitivity phenotypes of *cat-4*, *ptps-1* and *agmo-1* mutants. (A, B) SDS hypersensitivity of *cat-4, ptps-1* (A) and *agmo-1* (B) mutants. Synchronized adult gravid hermaphrodites were exposed to SDS in M9 buffer for 30 min, then examined for movement. Legend indicates alleles tested. (C, D) Levamisole hypersensitivity of *cat-4, ptps-1* (C) and *agmo-1* (D) mutants. Worms were exposed to levamisole in M9 for 5 min, then examined for movement. Legends as in A, B.

```
      SL1 spliced leader
   1  ggtttaattacccaagtttgagaactcttctccacaaaattttaaccaaatgaaaagaggatgaacgagacggca    75
                                                                     M  N  E  T  A

  76  tggttggatagggtattctcaaatacgtcactgggacatcgattgctggatagactaactctcacgaatcttcgt   150
       W  L  D  R  V  F  S  N  T  S  L  G  H  R  L  L  D  R  L  T  L  T  N  L  R
 151  cacgcattctaccttatctcaccttacgagaccaccgtcgagtcgattgatgatgttccgaactataacgctgaa   225
       H  A  F  Y  L  I  S  P  Y  E  T  T  V  E  S  I  D  D  V  P  N  Y  N  A  E
 226  gtttctgcgtggtggctggtgtttcttactgccgagtttttcatttattcatttccggacatgaagacagattt   300
       V  S  A  W  W  L  V  F  L  T  A  E  F  F  I  L  F  I  S  G  H  E  D  R  F

 301  gcactcaatgattcaataacgtcaatttgtgctggaatgctcagccaatgcttcaaatttggtggtcgagctgtc   375
       A  L  N  D  S  I  T  S  I  C  A  G  M  L  S  Q  C  F  K  F  G  G  R  A  V
 376  gcgatattcttgtacgtgattgtgtgggataactggcgaatattagaacctccatgggattccccgtggacatgg   450
       A  I  F  L  Y  V  I  V  W  D  N  W  R  I  L  E  P  P  W  D  S  P  W  T  W

 451  atttttgcctgttctttcaagattttatgtattatctggggcatcgggctgtgcatgaagccggcttcttctgg   525
       I  F  C  L  F  F  Q  D  F  M  Y  Y  L  G  H  R  A  V  H  E  A  G  F  F  W
 526  ggtcttcacacaattcaccatagctccgaatactacaatttctcaactgctctacgacaagctgccatacaagat   600
       G  L  H  T  I  H  H  S  S  E  Y  Y  N  F  S  T  A  L  R  Q  A  A  I  Q  D
 601  gctggattagcgatctatgactgtattcaggcattcttcatccctccatcaatattttagttcatcgatatttt   675
       A  G  L  A  I  Y  D  C  I  Q  A  F  F  I  P  P  S  I  F  L  V  H  R  Y  F

 676  tcggagattttccaatttatcatgcatacctcgttggtggacaccatgggaccccttggcttagtattcaacact   750
       S  E  I  F  Q  F  I  M  H  T  S  L  V  D  T  M  G  P  L  G  L  V  F  N  T
 751  ccgtctcatcatcgagtacatcatggaagaaatccgtattgtattgacaagaattatggaggagttttcattatt   825
       P  S  H  H  R  V  H  H  G  R  N  P  Y  C  I  D  K  N  Y  G  G  V  F  I  I

 826  tgggataagatgttcaacacatttgaagccgaacgtcacgatgacccaccaatctatggattagttaccaacgag   900
       W  D  K  M  F  N  T  F  E  A  E  R  H  D  D  P  P  I  Y  G  L  V  T  N  E
 901  aacactttcaatcaaatctacctccaattccatgctctttgggacattttaattttcaaaggattcacaaaagat   975
       N  T  F  N  Q  I  Y  L  Q  F  H  A  L  W  D  I  L  I  F  K  G  F  T  K  D
 976  gtgaaaggagagcccatgtttcctggagttgtgaacaaattgaaagcaaccgtattcccgccgggctggttccca  1050
       V  K  G  E  P  M  F  P  G  V  V  N  K  L  K  A  T  V  F  P  P  G  W  F  P
1051  ggggttcctgtcaccccgttctttcattggatgagcatggttaatccagctcacggagtacctgagccagagaaa  1125
       G  V  P  V  T  P  F  F  H  W  M  S  M  V  N  P  A  H  G  V  P  E  P  E  K
1126  cctgttctcagatacagcccacctgcgaggatcctagtgaaagtttacgtggcatcgtcattcttgttgttgttg  1200
       P  V  L  R  Y  S  P  P  A  R  I  L  V  K  V  Y  V  A  S  S  F  L  L  L  L
1201  gctatattcttccattttgaatacgaccggaatcatttgagctacttggattgtacagtcaagattgcatacttt  1275
       A  I  F  F  H  F  E  Y  D  R  N  H  L  S  Y  L  D  C  T  V  K  I  A  Y  F

1276  gtggttacgatgcaatgttttggagcatttttgatatgaaatggtatgcccggtacattgaaattgctcgttgt  1350
       V  V  T  M  Q  C  F  G  A  F  F  D  M  K  W  Y  A  R  Y  I  E  I  A  R  C
1351  tgtggagttctcatctattacggagtactcatgttcgatcatattggtgcaggaactcatcgtctttttgtcatt  1425
       C  G  V  L  I  Y  Y  G  V  L  M  F  D  H  I  G  A  G  T  H  R  L  F  V  I

1426  tcactgcatatcatggctattgcattgtggacgactgatgttttggtggagaaactctcccaatgctgctcaaag  1500
       S  L  H  I  M  A  I  A  L  W  T  T  D  V  L  V  E  K  L  S  Q  C  C  S  K
1501  aatcaatcaataaatccagaaaaaggtgacctggaacgggctccagaaattgcatcgatctcgaaaaatgttcaa  1575
       N  Q  S  I  N  P  E  K  G  D  L  E  R  A  P  E  I  A  S  I  S  K  N  V  Q
1576  taatatgattttatagagttgttgtttccattgtctttccaggctgtcatattctagtcactcttttcccttttt  1650
       *
1651  ttgtttgatggttttcttaaattaaagatttttctctcaaaaaa                                 1725
```

Figure S6 An *agmo-1* cDNA encodes a 505 amino acid protein. For *agmo-1*, we completed sequencing of cDNA yk1597b01 (previously partially sequenced, accession BJ763208) kindly provided by Yuji Kohara. The clone had a single nucleotide difference from genomic sequence that would result in a single amino acid difference from that predicted from genomic sequence (nt: T425C; aa: L122P) Both differences are marked in red. Introns located following the base indicated by a red arrow. The trans-spliced leader SL1 is underlined at the 5' end; the likely polyadenylation signal is underlined in the 3' UTR. We also sequenced twelve independent ORFeome clones (Reboul et al. 2001). We found clones of two different lengths, but both types appeared to be abnormal with an unspliced intron #4 which would result in a severely truncated protein. Genbank accession number for this *agmo-1* cDNA clone is KP290894.

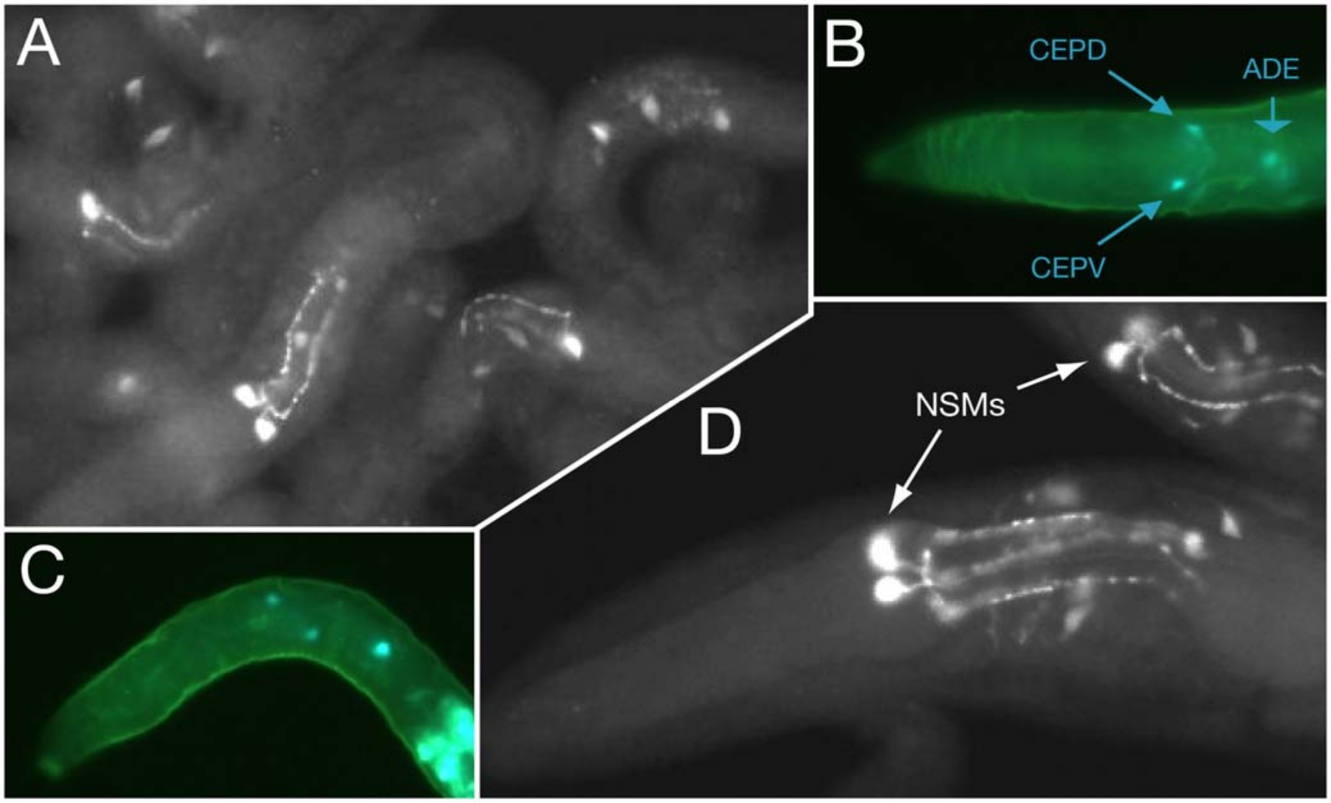

Figure S7 *agmo-1* mutants have normal 5HT and DA**.** (A, B) *agmo-1(e3047);* (C, D) *agmo-1(e3016).* Anti-5HT immunofluorescence (A, D) of worm heads of larvae (A, D) and adult (D) showing normal 5HT neurons. FIF (B, C) of larval heads showing normal DA neuron staining.

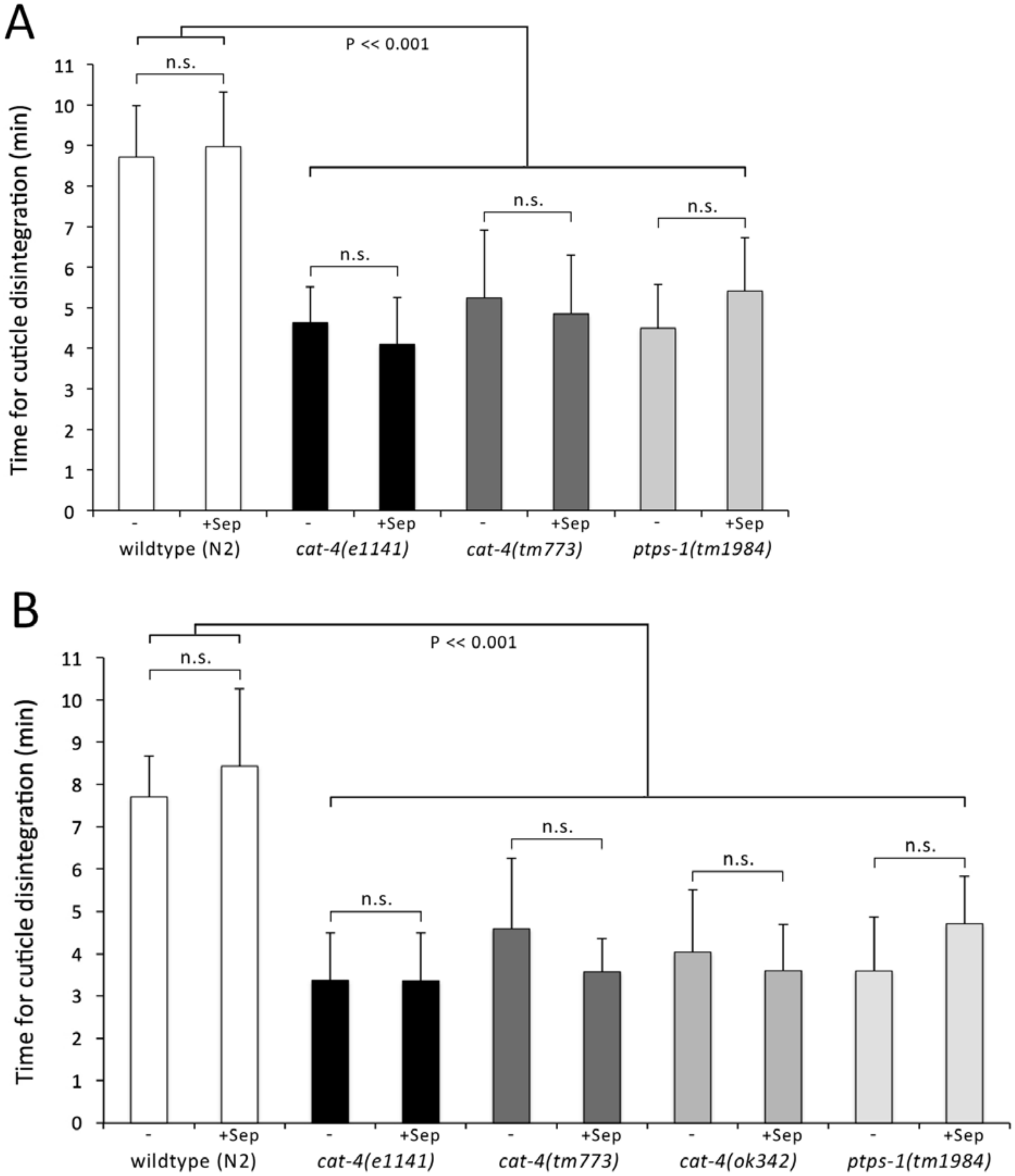

Figure S8 Exogenous biopterins do not rescue cuticle fragility in BH4-deficient mutants. (A-E) Cuticle disintegration time (mean ± S.D.) during mild alkaline bleach treatment, scored as first major cuticle rupture; worms from mixed stage cultures (n = 15), as in Fig 4 (B, C). For all experiments, groups were compared with 1-factor ANOVA followed by planned pairwise comparisons made with Scheffè's F-test (Sokal and Rohlf 1981). (A) Wild-type worms compared to biopterin deficient worms, with or without supplemental sepiapterin (100 µM). There were significant differences among the groups (overall ANOVA, P<< 0.0001). All pairwise comparisons between a genotype with sepiapterin (+Sep) or without (-) were not significant (n.s.). All pairwise comparisons of wild type with mutants were significantly different (P<<0.001). No biopterin deficient genotype was significantly different from another. (B) Additional experiment comparing wild-type to biopterin deficient worms, with or without supplemental sepiapterin, including *cat-4(ok342)*. Results like in (A). A customary approach in mammalian cells to increase BH4 intracellularly is to use sepiapterin (Werner-Felmayer et al. 2002; Aguado et al. 2006), since SR can effectively convert sepiapterin to 7,8 dihydrobiopterin (BH2) intracellularly, which is then reduced to BH4 by dihydrofolate reductase (DHFR). In *C. elegans,* we believe that other reductases substitute for SR and carry out the last steps in the synthesis of BH4 (since there is no ortholog); these enzymes should also be able to convert sepiapterin to BH2, the latter being further reduced to BH4 by DHFR in the so-called salvage pathway (Thöny et al. 2000).

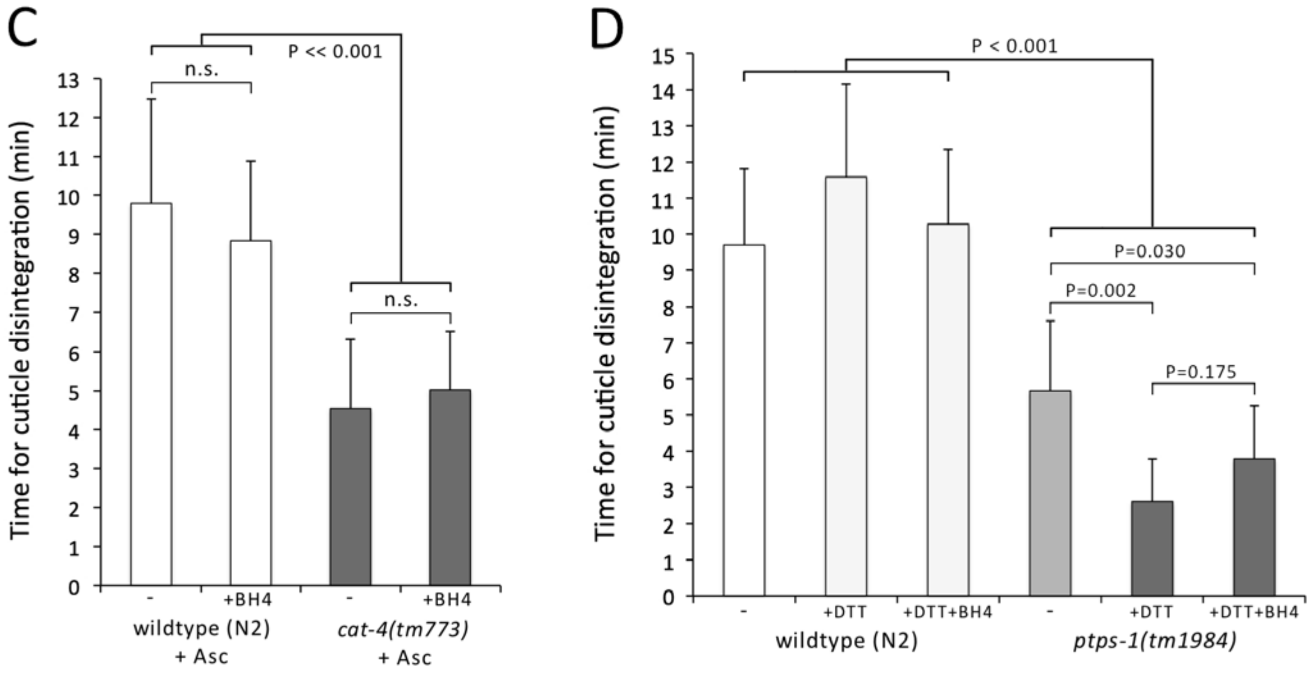

Figure S8 Exogenous biopterins do not rescue cuticle fragility in BH4-deficient mutants. (C) Supplementation with BH4 (200 µM), including ascorbate (Asc, 5 mM added to reduce oxidation) does not alter cuticle fragility in wild type or *cat-4(tm773)* mutant. Comparisons as in (A). (D) Supplementation with BH4 (200 µM) with or without dithiothreitol (DTT, 5 mm added to reduce oxidation) does not alter cuticle fragility in wild type (left columns, all N2 group comparisons not significant). Addition of DTT makes *ptps-1(tm1984)* mutant cuticles significantly more fragile (P<0.05 in pairwise comparisons with or without DTT). BH4 may reduce damage caused by DTT although the difference is not statistically significant (P=0.175). DTT can reduce disulfide bonds in the highly cross-linked *C. elegans* cuticle (Stenvall et al. 2011).

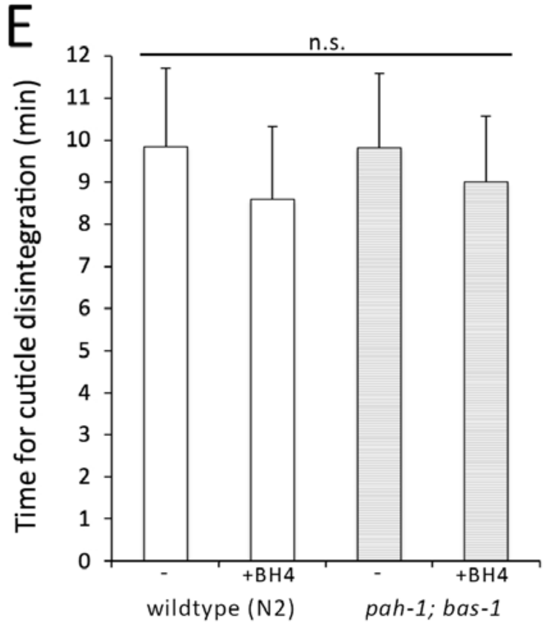

Figure S8 Exogenous biopterins do not rescue cuticle fragility in BH4-deficient mutants. (E) Loss of PAH activity, 5HT and DA do not affect cuticle fragility; supplementation with BH4 does not affect cuticle fragility of wild type or *pah-1; bas-1* mutant. There were no significant differences among the groups in the overall 1-factor ANOVA. Double mutant *pah-1(tm520); bas-1(ad446)* lacks PAH and aromatic amino acid decarboxylase activities; *bas-1* mutants are 5HT- and DA-deficient (Hare and Loer 2004; Calvo et al. 2008).

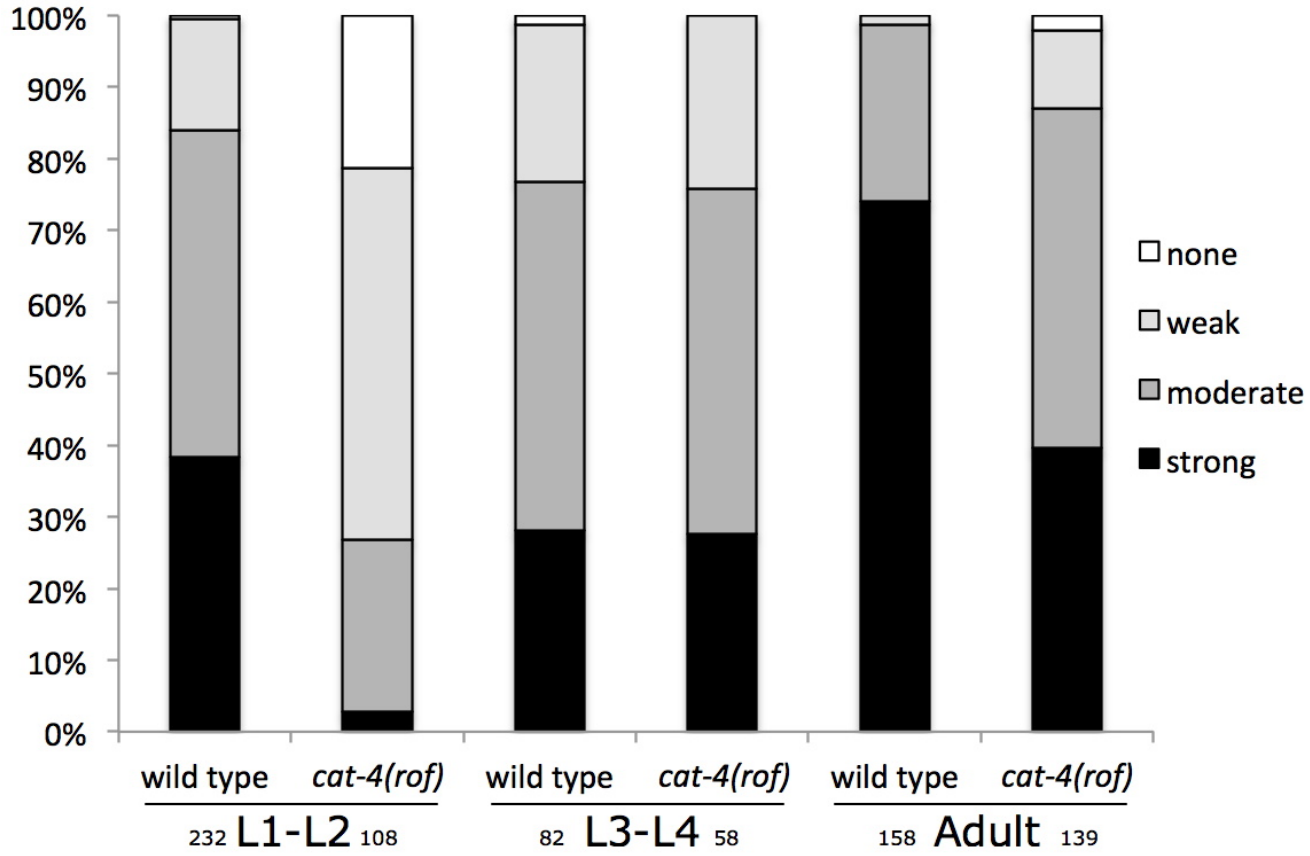

Figure S9 *cat-4* reduction-of-function mutants accumulate 5HT during larval development. Anti-5HT immunoreactivity differences in wild type and *cat-4(e3015)* reduction of function *(rof)* are most apparent in young larvae. 85% of wild-type worms are strongly to moderately stained whereas only 27% of *cat-4(e3015)* L1-L2 worms are strongly to moderately stained. Mutants in mixed populations of young (L1-L2), older larvae (L3-L4), and adults, scored for intensity of NSM cell body staining (as in Fig. 7A, B). Numbers of worms scored shown below the column.

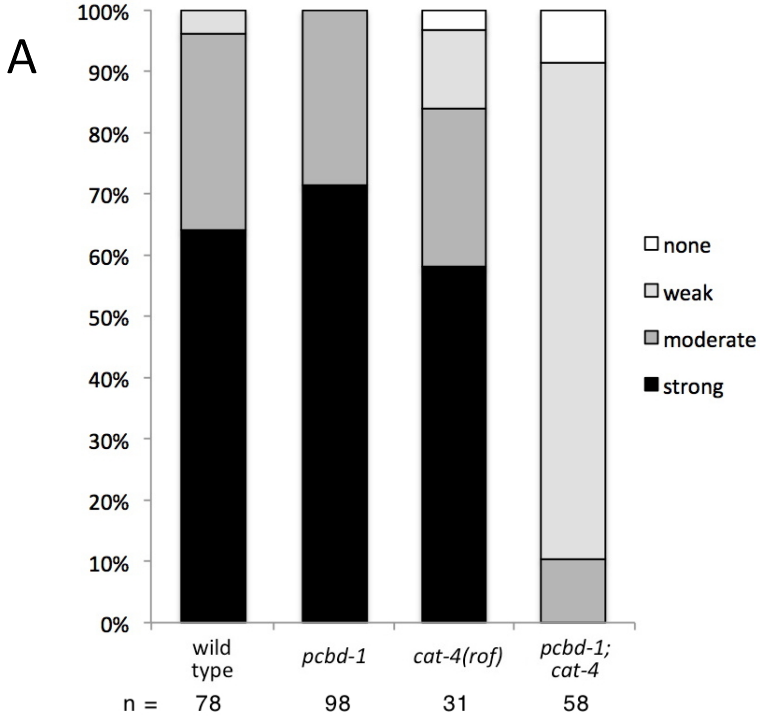

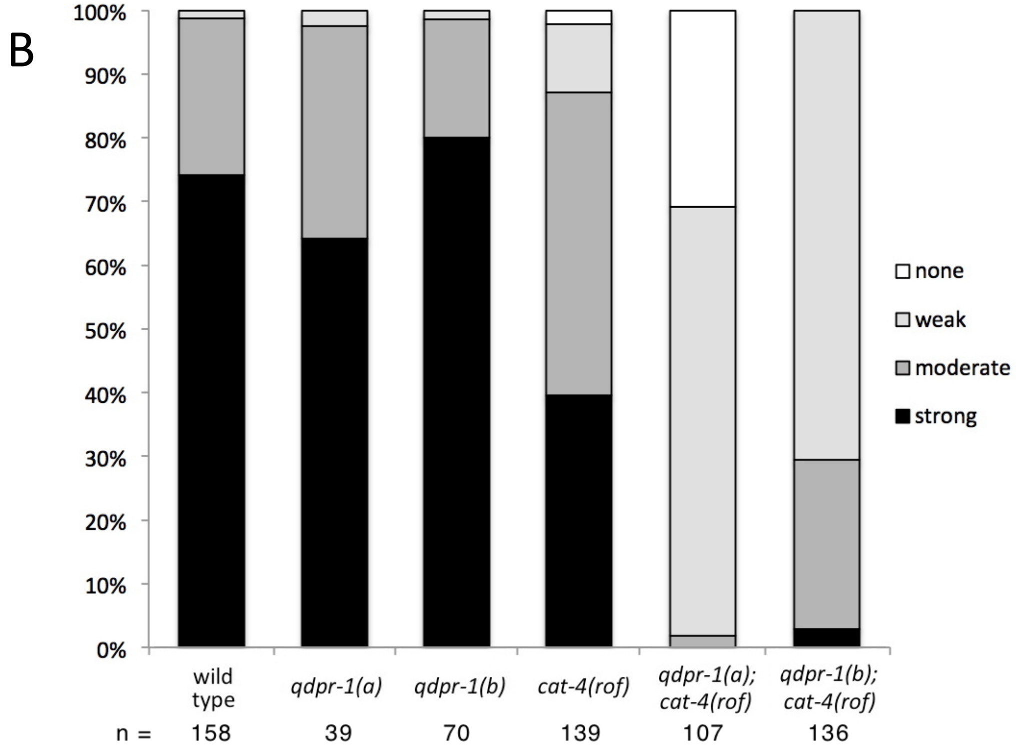

Figure S10 Biopterin regeneration genes *pcbd-1* and *qdpr-1* function in 5HT synthesis in adult worms. Anti-5HT immunoreactivity differences in wild type, single mutant, and double mutants in adult worms (see legend for Fig 7A, B). Numbers of worm scored shown below the columns. (A) *pcbd-1* single and double mutants with *cat-4(e3015)*. (B) *qdpr-1* single and double mutants with *cat-4(e3015).*

A

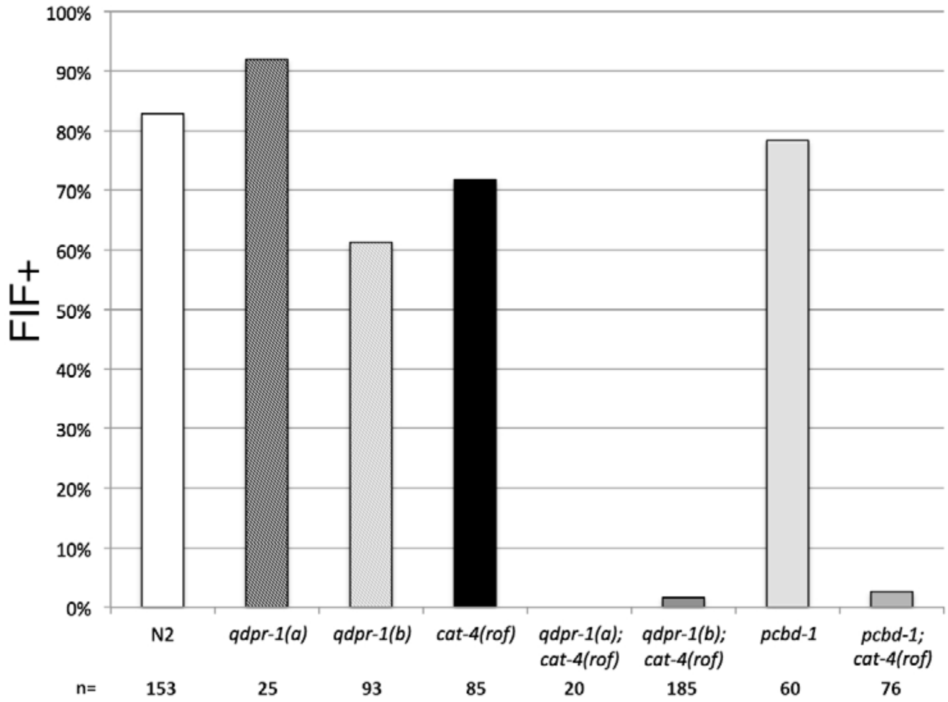

B

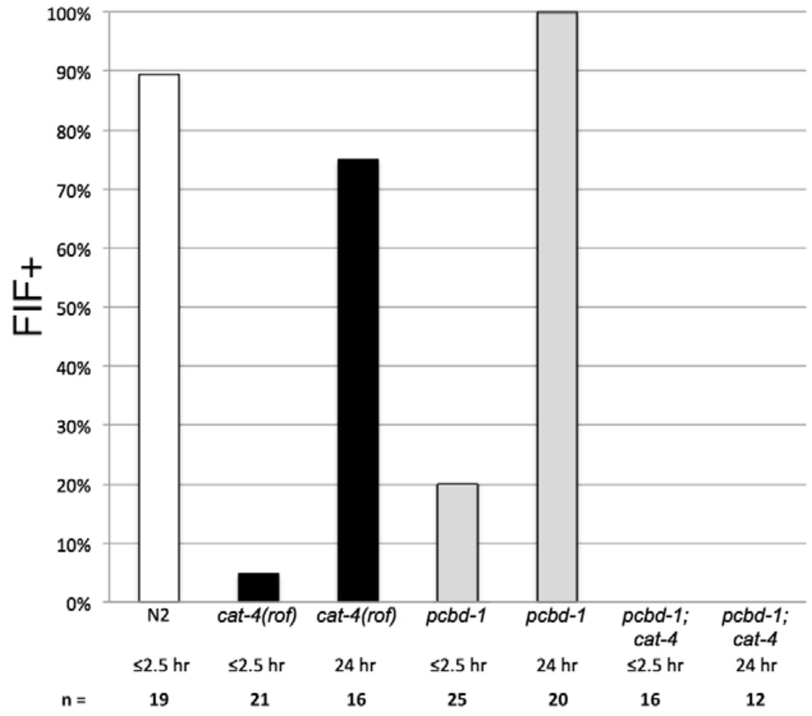

Figure S11 Biopterin regeneration genes *pcbd-1* and *qdpr-1* function in DA synthesis. (A) Mixed populations, young worms (L1-L2) scored for DA by formaldehyde induced fluorescence (FIF). Y axis – percentage of worms with one or more FIF+ head neurons (without regard to brightness). Alleles used: *cat-4(rof)* = 'reduction of function' = *e3015*; *qdpr-1(a) = tm2337; qdpr-1(b) = tm2373; pcbd-1(tm5924).* (B) Loss of DA in BH4 synthesis and regeneration mutants is most apparent in very young worms. Staged worms scored for DA by FIF – early L1s (≤ 2.5 hr post-hatching), mid-L2s (~ 24 post-hatching). Y axis and alleles used as in (A). Eggs were picked to a seeded plate and allowed to hatch for 2.5 hr, at which time hatchling worms were immediately subjected to FIF staining, or transferred to a new seeded plate, incubated at 20°, and tested by FIF 24 hr later.

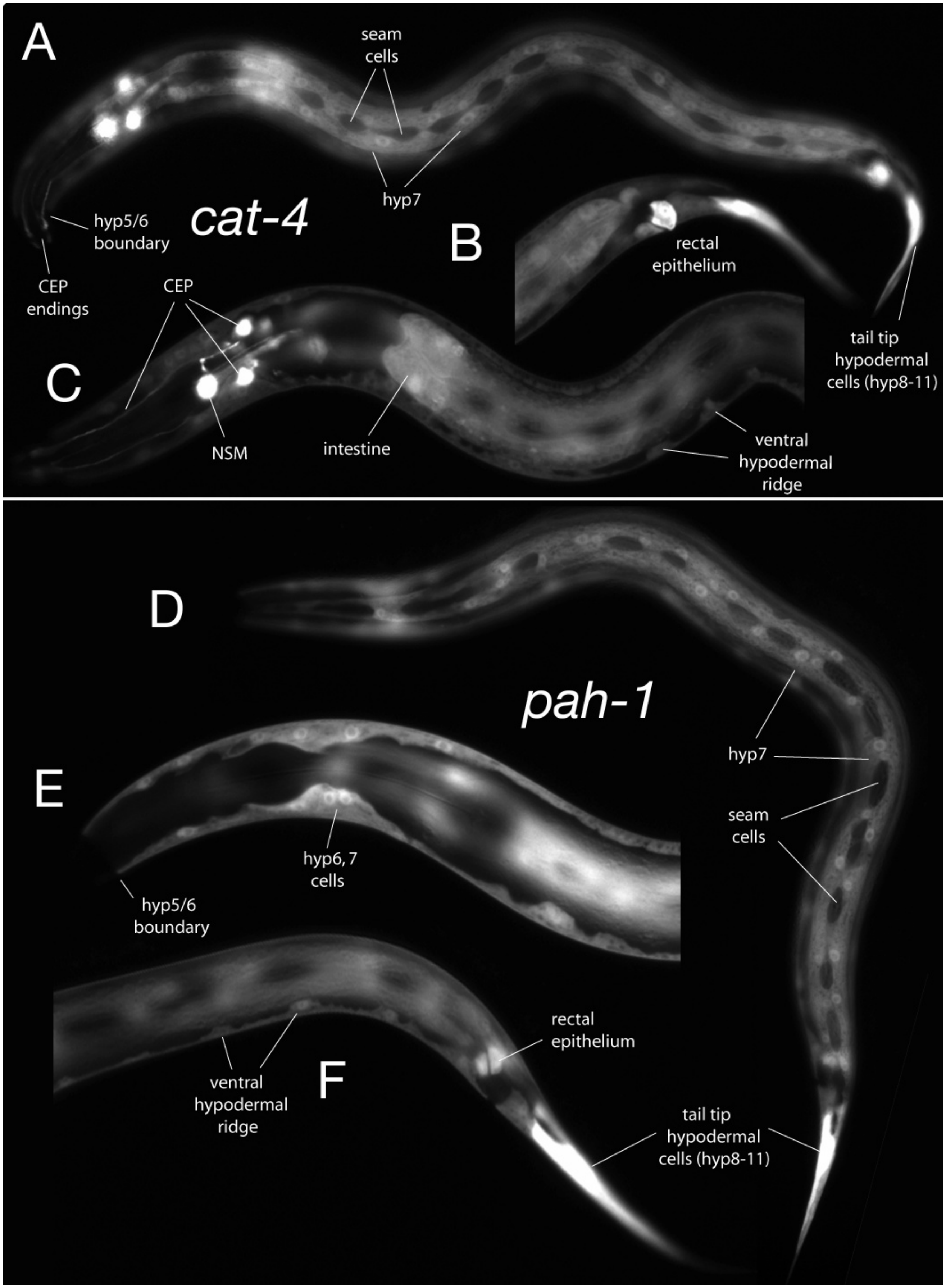
A
seam cells
hyp7
hyp5/6 boundary
CEP endings
cat-4
B
rectal epithelium
tail tip hypodermal cells (hyp8-11)
CEP
C
NSM
intestine
ventral hypodermal ridge
D
pah-1
hyp7
seam cells
E
hyp6, 7 cells
hyp5/6 boundary
rectal epithelium
F
ventral hypodermal ridge
tail tip hypodermal cells (hyp8-11)

Figure S12 Expression of *cat-4* and *pah-1* GFP reporters. Anterior is to the left in all worms. (A-C) *cat-4*::GFP transgenic L2 stage worms, repeated from Fig 8A to facilitate comparison with *pah-1*::GFP expression (D-F). (A) Superficial focal plane showing epidermal expression, especially in hyp7 syncytium. Seam cells have undergone doubling division and can be seen along the lateral side as darker regions among the brightly staining hyp7 cell. Dendritic endings of CEP neurons can be seen at the tip of the 'nose.' (B) Medial focal plane showing anal cells, strongly expressing tail epidermal cells and posterior intestinal cells expressing GFP. (C) Medial focal plane showing epidermal expression in the body and head, and the boundary between hyp6 and unstained hyp5. NSM and CEP neuron somas are seen in the head, plus some neuronal processes (especially CEP processes). A few other neuronal somas stain less brightly. The anteriormost intestine also shows GFP expression, as do some rectal epithelial cells (here in what appear to be B & Y cells). (D-F) *pah-1*::GFP transgenic L2 stage worms. (D) Superficial focal plane showing especially hyp7 expression, with dark seam cells (post-doubling division) lying over hyp7. (E) Medial focal plane showing anterior epidermal expression. The hyp6 (expressing) / hyp5 (dark, not expressing) boundary is very clear here. (E) Medial focal plane in the posterior showing intestine, some rectal epithelial cells, and strongly expressing epidermal tail cells. (F) Medial focal plane showing ventral hypodermal ridge, rectal epithelium cells, and strongly expressing tail epidermal cells expressing GFP.

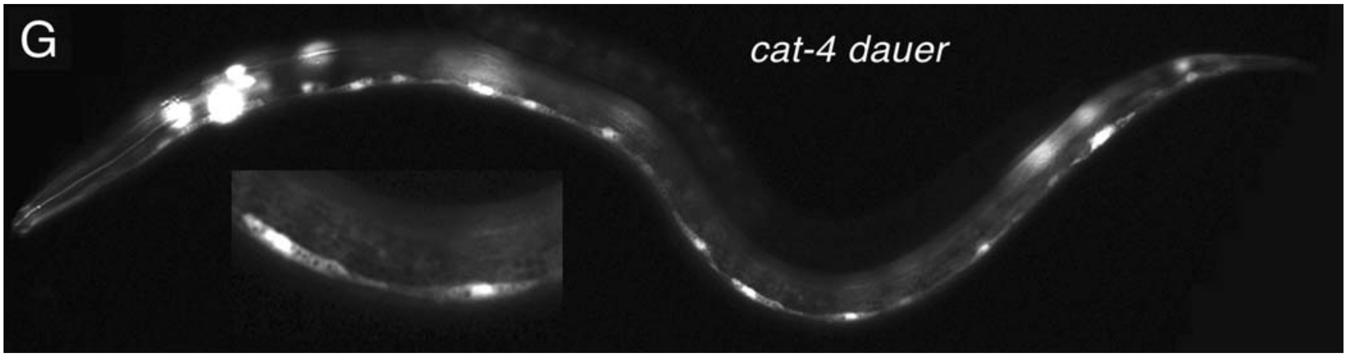

Figure S12 (G) *cat-4*::GFP worm showing expression in lateral epidermal seam cells during dauer stage. Anterior to the left. Inset: closeup of two seam cells in midbody region.

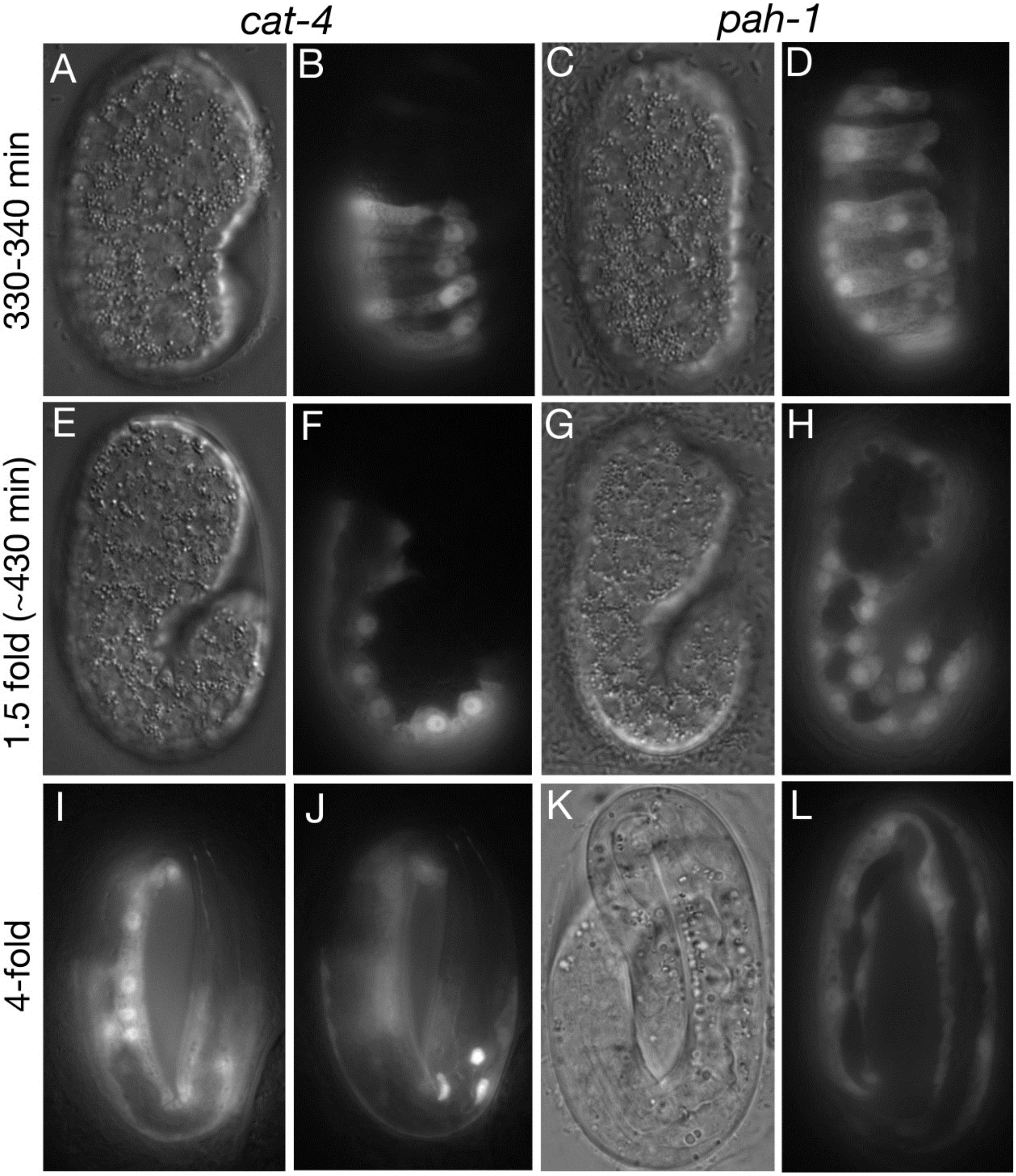

Figure S13 Expression of *cat-4*, *pah-1* and *ptps-1* reporter constructs in embryos. Left hand columns (A, B, E, F, I, J) - *cat-4*::GFP embryos; Right hand columns (C, D, G, H, K, L) *pah-1*::GFP embryos. Pairs of photos (e.g., A+B) have DIC view of embryos (A) matched with GFP fluorescence (B) in same superficial focal plane. Top row (A-D): 330-340 min, post nuclear counter-migration. Embryos have just begun elongation, dorsal view, anterior up. (B) *cat-4*::GFP embryo shows expression in posterior dorsal epidermal cells. (D) *pah-1*::GFP is expressed in both anterior and posterior dorsal epidermal cells. Middle row (E-H). 1.5-fold stage, lateral view. (F) *cat-4*::GFP expression is seen in posterior dorsal epidermal cells. (H) *pah-1*::GFP is expressed in most dorsal and ventral epidermal cells, but not in lateral seam cells. Bottom row (I-L). Late (4-fold) embryos. (I) Superficial focal plane of anterior of worm showing *cat-4*::GFP epidermal staining (except in seam cells) similar to that seen in larvae. CEP neurons dendritic endings are seen in tip of nose. (J) Deeper focal plane of same worm showing NSM and CEP neurons expressing GFP. (K) DIC view of *pah-1*::GFP late embryo (4-fold). (L) Different, superficial focal plane of same worm.

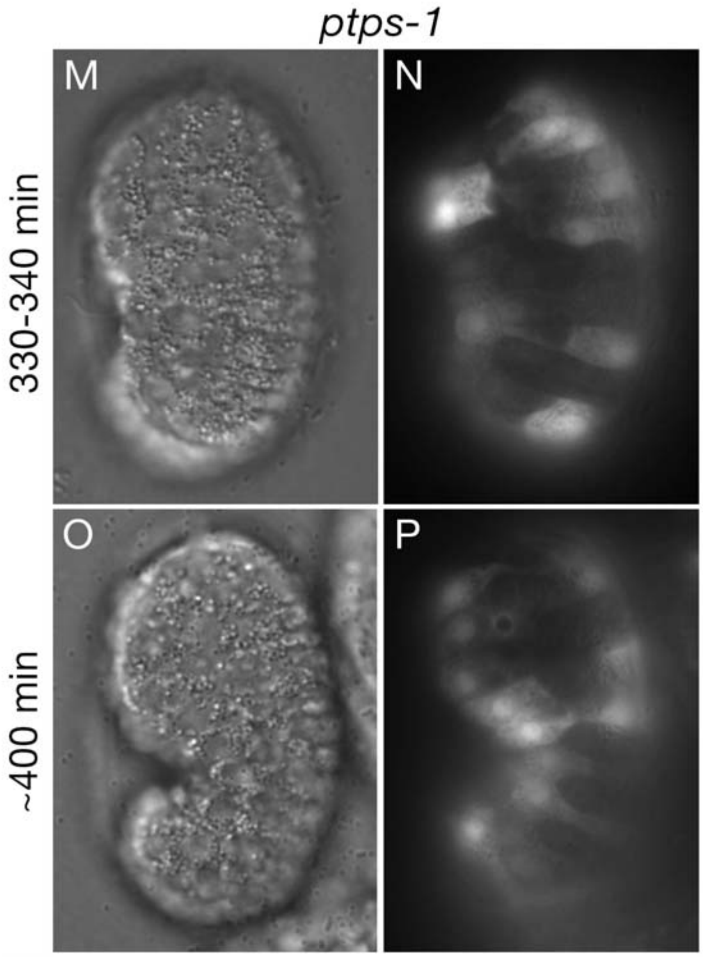

Figure S13 Expression of *cat-4*, *pah-1* and *ptps-1* reporter constructs in embryos. (M-P) *ptps-1*::GFP embryos (strain OH11619). Pairs of matching photos showing DIC view of embryo with GFP fluorescence at the indicated times. Expression seen in both anterior and posterior dorsal epidermal cells

File S1

Supporting Materials and Methods

Worms strains used in this study:

N2 wild type
CB369 *unc-51(e369)*
HA1335 *cat-4(ok342)V*
VC20144 *cat-4(gk245686)V*
CLB49 [line 4] *glsEx3 [rol-6(dom) cat-4(+)]; cat-4(tm773)V*
CB7130 *him-8(e1489)IV; cat-4(e3030)V*
LC84 *ptps-1(tm1984)I; him-5(e1490)V*
LC87 *qdpr-1(tm2337)III*
LC133 *pcbd-1(tm5924)I*
LC130 *qdpr-1(tm2373)III; cat-4(e3015)V*
LC83 *pah-1(tm520); bas-1(ad446)*
CB7127 *agmo-1(e3019)III*
CB7129 *agmo-1(e3047)III*
CB1490 *him-5(e1490)V*
CB1141 *cat-4(e1141)V*
LC81 *cat-4(tm773)V*
CLB49 [line 1] *glsEx1 [rol-6(dom) cat-4(+)]; cat-4(tm773)V*
CB7107 *him-8(e1489)IV; cat-4(e3015)V*
LC80 *ptps-1(tm1984)I*
CB7094 *ptps-1(e3042)I*
LC90 *qdpr-1(tm2373)III*
LC129 *qdpr-1(tm2337)III; cat-4(e3015)V*
LC131 *pcbd-1(tm5924)I; cat-4(e3015)V*
CB7014 *agmo-1(e3016)III*
CB7128 *agmo-1(e3029)III*
VC1198 *Y39G8B.1(ok1682)II*

See also transgenic reporter fusion strains listed in Tables S1, S2.

Table S1 Biopterin-related GFP reporter gene transgenics (this work) – primers and characteristics

Gene	Primers†	Sequence	Fusion type	Size (bp)**	Transgenics ‡
cat-4	AC1763 (A)<br>AC1764 (A*)<br>AC1765 (B)	AAAGGTTGCATGTTGCAGATG<br>TGCATGTTGCAGATGGAAATTG<br>CATTTTGATATTATGATGTTGATAGA	Transcriptional, through ATG	2654	CZ9718, -19 *(juEx2054, -5)*
ptps-1	AC3051 (A)<br>AC3050 (B)	ATAGTCCGGTCTTGTACCAC<br>ACATCCTTTATAAGTGAAAATATT	Translational, in final coding exon	2751 (~2000)	CZ18320, -21, -22 *(juEx5474, -5, -6)*
	AC3049 (A)§	TCGAATTTTCGCGGACAAGG	Translational, in final coding exon	1160 (~450)	CZ18092, -93 *(juEx5477, -8)*
pcbd-1	AC3217 (A)<br>AC3218 (B)	ATCAGAGTAGGAGTCAGGGAG<br>TGAATGAGAAGATGCGTTGAGAAG	Transcriptional, upstream of ATG	1505	CZ19212 *(juEx5785)*
qdpr-1	AC3219 (A)<br>AC3220 (B)	CGGTATCCTTGTCGCCAAAC<br>TGTTCGAAACTGCAAGGAAAAAGT	Transcriptional, upstream of ATG	1048	CZ19213, -14 *(juEx5786, -7)*
qdpr-1	qdpr1AL1 (A)<br>qdpr1AL2 (A*)<br>qdpr1BR (B)	TGGCGGAATCGATTTATTTG<br>GCCATATTGCGTTCAATGAG<br>TCGATGTTCCATTTTCAGTGG	Translational, in final coding exon	4887 (~4000)	CZ19215 *(juEx5788)*
gfrp-1	AC3221 (A)<br>AC3222 (B)	AATACGGTGCCAGGTGTCAG<br>TTCTTGATTTTTTTGTTGCGGAAT	Transcriptional, upstream of ATG	1088	CZ19216 *(juEx5789)*
agmo-1	AC3030 (A)<br>AC3031 (B)	ACTTGGCAAACAGTTGGAAGC<br>CCTCTTTTCATTTGGTTAAAATTT	Transcriptional, upstream of ATG	2281	CZ17563, -64, -65 *(juEx5238, -39, -40)*
pah-1	AC1769 (A)<br>AC1770 (A*)<br>AC1771 (B)	ATGGAAGGTCAGATTGGATATC<br>TCAGATTGGATATCTTCCACG<br>CATTTTCAGAGAACAAGATTTGGT	Transcriptional, through ATG of an isoform	2005	CZ9720, -21 *juEx2056, -7*

† - Primer name ('style' of primer, ala Hobert, 2002); not shown above, all 'B' primers begin with:
AGTCGACCTGCAGGCATGCAAGCT (sequence overlapping GFP coding to create fusion)

§ - Primer paired with the same B (AC3050)

** - Length of sequence upstream to GFP (length upstream of predicted translation start if translational fusion)

‡ - Strain number (beginning with CZ) and allele designation of extrachromosomal transgenes (*juEx*)

Table S2 Biopterin-related GFP reporter gene transgenics from other sources

Gene	Fusion type	Size (bp)**	Transgenic	Reference
cat-4	translational, exon 2	~2800 (~2000)	JY739†	Sze et al. 2002†
cat-4	transcriptional	~630	OH8482	Flames and Hobert 2009
ptps-1	transcriptional	~2600	OH11619	Zhang et al. 2014
pcbd-1	transcriptional	~800	OH12385	Zhang et al. 2014
qdpr-1	transcriptional	~700	OH12386	Zhang et al. 2014
gfrp-1	translational, exon 2	~2500 (~1400)	OH12387	Zhang et al. 2014

** - length of sequence upstream to GFP (length upstream of predicted translation start if translational fusion).
† - this strain shows expression in biogenic amine neurons and the *epidermis, not in muscle cells* as reported.

References for Supporting Information

Aguado, C., B. Perez, M. Ugarte and L. R. Desviat, 2006. Analysis of the effect of tetrahydrobiopterin on PAH gene expression in hepatoma cells. FEBS Lett 580: 1697-1701.

Baker, R. H., C. Britton, B. Roberts, C. M. Loer, J. B. Matthews and A. J. Nisbet, 2012. Melanisation of *Teladorsagia circumcincta* larvae exposed to sunlight: a role for GTP-cyclohydrolase in nematode survival. Int J Parasitol 42: 887-891.

Calvo, A. C., A. L. Pey, M. Ying, C. M. Loer and A. Martinez, 2008. Anabolic function of phenylalanine hydroxylase in *Caenorhabditis elegans*. FASEB J 22: 3046-3058.

Flames, N. and O. Hobert, 2009. Gene regulatory logic of dopamine neuron differentiation. Nature 458: 885-889.

Hare, E. E. and C. M. Loer, 2004. Function and evolution of the serotonin-synthetic *bas-1* gene and other aromatic amino acid decarboxylase genes in *Caenorhabditis*. BMC Evol Biol 4: 24.

Hoekstra, R., A. Visser, M. Otsen, J. Tibben, J. A. Lenstra and M. H. Roos, 2000. EST sequencing of the parasitic nematode *Haemonchus contortus* suggests a shift in gene expression during transition to the parasitic stages. Mol Biochem Parasitol 110: 53-68.

Loer, C. M. and C. J. Kenyon, 1993. Serotonin-deficient mutants and male mating behavior in the nematode *Caenorhabditis elegans*. J Neurosci 13: 5407-5417.

Mitreva, M., A. A. Elling, M. Dante, A. P. Kloek, A. Kalyanaraman, S. Aluru, S. W. Clifton, D. M. Bird, T. J. Baum and J. P. McCarter, 2004. A survey of SL1-spliced transcripts from the root-lesion nematode *Pratylenchus penetrans*. Mol Genet Genomics 272: 138-148.

Moore, J., L. Tetley and E. Devaney, 2000. Identification of abundant mRNAs from the third stage larvae of the parasitic nematode, *Ostertagia ostertagi*. Biochem J 347 Pt 3: 763-770.

Nisbet, A. J., D. L. Redmond, J. B. Matthews, C. Watkins, R. Yaga, J. T. Jones, M. Nath and D. P. Knox, 2008. Stage-specific gene expression in *Teladorsagia circumcincta* (Nematoda: Strongylida) infective larvae and early parasitic stages. Int J Parasitol 38: 829-838.

Reboul, J., P. Vaglio, N. Tzellas, N. Thierry-Mieg, T. Moore, C. Jackson, T. Shin-i, Y. Kohara, D. Thierry-Mieg, J. Thierry-Mieg, H. Lee, J. Hitti, L. Doucette-Stamm, J. L. Hartley, G. F. Temple, M. A. Brasch, J. Vandenhaute, P. E. Lamesch, D. E. Hill and M. Vidal, 2001. Open-reading-frame sequence tags (OSTs) support the existence of at least 17,300 genes in *C. elegans*. Nat Genet 27: 332-336.

Sokal, R. R. and F. J. Rohlf, 1981. *Biometry*. New York, WH Freeman.

Stenvall, J., J. C. Fierro-Gonzalez, P. Swoboda, K. Saamarthy, Q. Cheng, B. Cacho-Valadez, E. S. Arner, O. P. Persson, A. Miranda-Vizuete and S. Tuck, 2011. Selenoprotein TRXR-1 and GSR-1 are essential for removal of old cuticle during molting in *Caenorhabditis elegans*. Proc Natl Acad Sci U S A 108: 1064-1069.

Sze, J. Y., S. Zhang, J. Li and G. Ruvkun, 2002. The C. elegans POU-domain transcription factor UNC-86 regulates the *tph-1* tryptophan hydroxylase gene and neurite outgrowth in specific serotonergic neurons. Development 129: 3901-3911.

Thompson, O., M. Edgley, P. Strasbourger, S. Flibotte, B. Ewing, R. Adair, V. Au, I. Chaudhry, L. Fernando, H. Hutter, A. Kieffer, J. Lau, N. Lee, A. Miller, G. Raymant, B. Shen, J. Shendure, J. Taylor, E. H. Turner, L. W. Hillier, D. G. Moerman and R. H. Waterston, 2013. The million mutation project: A new approach to genetics in *Caenorhabditis elegans*. Genome Res 23: 1749-1762.

Thöny, B., G. Auerbach and N. Blau, 2000. Tetrahydrobiopterin biosynthesis, regeneration and functions. Biochem J 347: 1-16.

Werner-Felmayer, G., G. Golderer and E. R. Werner, 2002. Tetrahydrobiopterin biosynthesis, utilization and pharmacological effects. Curr Drug Metab 3: 159-173.

Zhang, F., A. Bhattacharya, J. C. Nelson, N. Abe, P. Gordon, C. Lloret-Fernandez, M. Maicas, N. Flames, R. S. Mann, D. A. Colon-Ramos and O. Hobert, 2014. The LIM and POU homeobox genes *ttx-3* and *unc-86* act as terminal selectors in distinct cholinergic and serotonergic neuron types. Development 141: 422-435.
