# Supplementary material for: Cuticle Integrity and Biogenic Amine Synthesis in Caenorhabditis elegans Require the Cofactor Tetrahydrobiopterin (BH4)
Source: Genetics. 2015 Mar 24;200(1):237–53. doi: 10.1534/genetics.114.174110 (PMC4423366; doi:10.1534/genetics.114.174110)
Supplement: Supporting Information [file supp_114.174110_FigureS1.pdf]

```
 SL1 spliced leader
ggtttaattacccaagtttgaggctttacccactctatcaacatcataatatcaaa

atgtccagaattgagaacgaaagcggatttttgtcatctgacgccgcaagtgttggatcc
 M  S  R  I  E  N  E  S  G  F  L  S  S  D  A  A  S  V  G  S

gaagacgataaagttgagatgaagaaaagaaacggaacgattccaaaggaagatcatttg
 E  D  D  K  V  E  M  K  K  R  N  G  T  I  P  K  E  D  H  L
                                          ↓
aaatcaatgtgcaatgcctatcagagcatcattcaacatgtcggagaagacatcaatcgt
 K  S  M  C  N  A  Y  Q  S  I  I  Q  H  V  G  E  D  I  N  R

cagggacttctgaaaactccagaacgtgctgccaaagcaatgatggcattcacaaaggga
 Q  G  L  L  K  T  P  E  R  A  A  K  A  M  M  A  F  T  K  G
                  ↓
tacgatgatcaacttgatgagctcctcaacgaggcagtattcgacgaggatcacgatgag
 Y  D  D  Q  L  D  E  L  L  N  E  A  V  F  D  E  D  H  D  E

atggttattgtgaaagatattgaaatgttctccctttgtgagcatcatttagttccattt
 M  V  I  V  K  D  I  E  M  F  S  L  C  E  H  H  L  V  P  F

atgggaaaagttcatattggatacattccaaacaagaaggttcttggtctgtccaagttg
 M  G  K  V  H  I  G  Y  I  P  N  K  K  V  L  G  L  S  K  L
                                    ↓
gcaagaatagtcgagatgttcagcagaagacttcaagtccaagaacgtcttacaaagcaa
 A  R  I  V  E  M  F  S  R  R  L  Q  V  Q  E  R  L  T  K  Q

attgccaccgcaatggttcaagctgtacaaccatccggagttgcagttgttattgaagct
 I  A  T  A  M  V  Q  A  V  Q  P  S  G  V  A  V  V  I  E  A

agtcacatgtgtatggtaatgagaggagttcaaaagatcaatgcttccaccacaacatcc
 S  H  M  C  M  V  M  R  G  V  Q  K  I  N  A  S  T  T  T  S

tgcatgttgggagtattccgtgacgatccaaagactcgcgaagaattccttaatcttatc
 C  M  L  G  V  F  R  D  D  P  K  T  R  E  E  F  L  N  L  I

aacaaacgctaaaaattctagtcatttcctttttttacttgtttttttgggttttaactgt
 N  K  R

tactaacttttcataactttccatgtgaatctactaacatcttatgcttcagctttttaa

attcatacaacatgttcattgcctgg
```

Figure S1 *cat-4* cDNA sequence encodes a 223 amino acid protein. We isolated, cloned and sequenced *cat-4* cDNAs by RT-PCR from mixed stage *C. elegans him-5* worms as previously described (Hare and Loer 2004); cDNA clones were also obtained from the ORFeome project (Reboul et al. 2001). For *cat-4*, twelve independent clones from pooled ORFeome DNA were partially sequenced; 11/12 were identical F32G8.6 cDNAs, 1/12 was a different non-F32G8.6 sequence. We found that *cat-4* message is trans-spliced to SL1 (underlined at the 5' end). All cDNA clones we examined and those found in databases from various transcriptomics projects showed the same pattern of splicing, indicating there is likely a single type of mRNA and therefore a single isoform of the worm GTPCH1 protein with 223 amino acids. Introns are located in genomic sequence following the base indicated by a red arrow. Genbank accession numbers associated with these *cat-4* cDNA clones are KP290890-KP290893.

We also used RT-PCR to isolate *cat-4* cDNAs from *C. briggsae* which were very similar to those from *C. elegans*. RT-PCR from the diplogastrid nematode *Pristionchus pacificus* yielded 2 different cDNAs: a 9 exon transcript encoding a 270 aa protein, and an 8 exon transcript encoding a 245 aa protein. The longer cDNA matches some gene predictions, whereas the shorter cDNA encodes a more typical GTPCH1 protein.

It is notable that GTPCH1 transcripts are among the most abundant transcripts in some nematodes. For example, among SL1-spliced ESTs from a mixed stage library from the plant parasitic nematode *Pratylenchus penetrans,* 6% were from GTPCH1 (Mitreva et al. 2004). In several mammalian gastrointestinal parasites (e.g., *Ostertagia ostertagia*, *Haemonchus contortus*, *Teladorsagia circumcincta),* a significant fraction of transcripts – up to 30% – from infective L3 stage larvae encoded GTPCH1 (Hoekstra et al. 2000; Moore et al. 2000; Nisbet et al. 2008).

We also examined worms with the *cat-4* missense allele *gk245686* from the Million Mutation Project (Thompson et al. 2013). In this mutant, there is a relatively conservative change (M185I), although the alteration is in an amino acid that is 100% conserved in metazoans, and likely within the active site (see Fig 1). Perhaps surprisingly, worms with this mutation had no obvious neurotransmitter deficiency or bleach hypersensitivity, although the change is conservative.
