# Supplementary material for: Cuticle Integrity and Biogenic Amine Synthesis in Caenorhabditis elegans Require the Cofactor Tetrahydrobiopterin (BH4)
Source: Genetics. 2015 Mar 24;200(1):237–53. doi: 10.1534/genetics.114.174110 (PMC4423366; doi:10.1534/genetics.114.174110)
Supplement: Supporting Information [file supp_114.174110_FigureS2.pdf]

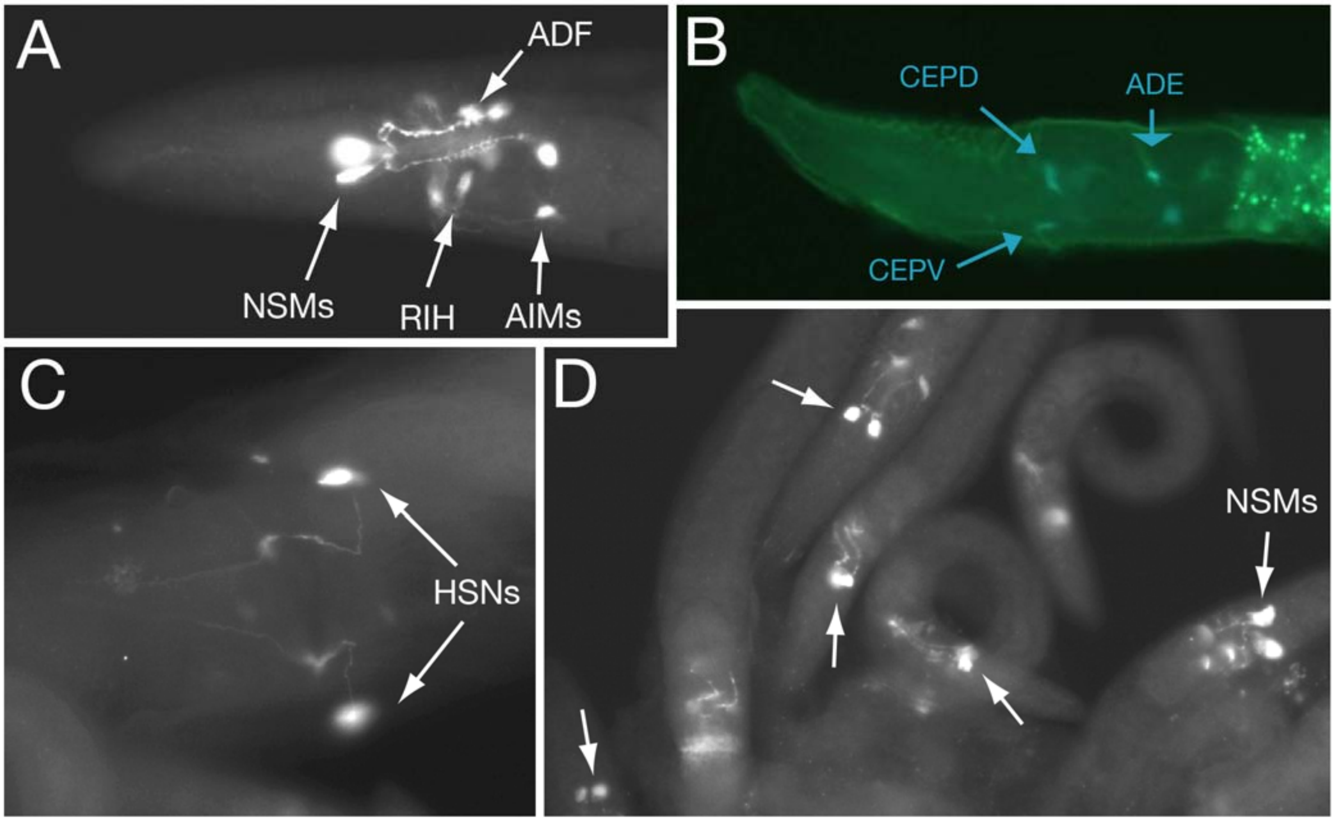

Figure S2 Rescue of neurotransmitter synthesis in *cat-4* mutants by genomic F32G8.6-containing sequence. Anti-5HT immunofluorescence (A, C, D) or FIF (B) of adult and larval *cat-4(tm773)* worms rescued with F32G8.6-containing plasmid (from Baker et al. 2012). (A) Head of adult worm showing presence of all normal serotonergic neurons; somas indicated with arrows. NSM neurites are apparent. (B) FIF of larval worm head showing presence of normal complement of DA neurons. (C) Adult hermaphrodite serotonergic HSN egg-laying neurons innervating vulval region and extending neurites anteriorly to the head. (D) Many rescued (5HT positive) larvae. NSM neuron somas indicated with arrow.
