# Supplementary material for: Cuticle Integrity and Biogenic Amine Synthesis in Caenorhabditis elegans Require the Cofactor Tetrahydrobiopterin (BH4)
Source: Genetics. 2015 Mar 24;200(1):237–53. doi: 10.1534/genetics.114.174110 (PMC4423366; doi:10.1534/genetics.114.174110)
Supplement: Supporting Information [file supp_114.174110_FigureS3.pdf]

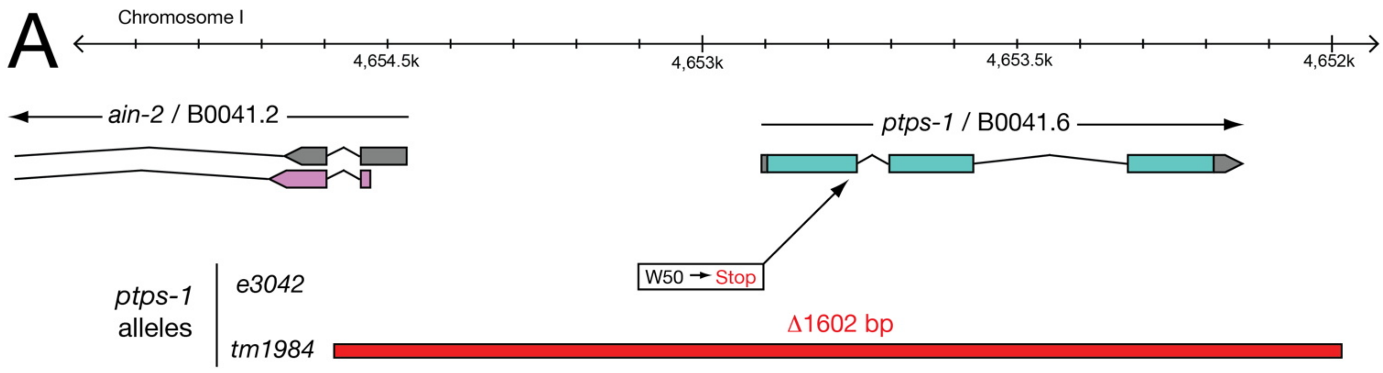

B

```
                                                                                                    e3042
Cel_PTPS  ------------------------------MFRMPIVTMERVDSFSAAHRLHSEKLSDAENKETFGKCNNSNGHGHNYVWKVKLRGEVDPTSGMVYDLAKLKKEMS-LVLDTVDHRNL  87
Dme_PTPS  ------------------------------MSQQPVAFLTRRETFSACHRLHSPQLSDAENLEVFGKCNNFHGHGHNYTVEITVRGPIDRRTGMVLNITELKEAIETVIMKRLDHKNL  88
Spu_PTPS  ------------------------------MSDDFDIEAL-NGGFP-----MSPELSDDENIKLYDKCNNPNGHGHNYKVELTLRGKVYPVTGMVMNISDLKAYMKNGFLDLMDHKNL  82
Cin_PTPS  ----------------------------------MKVFLTRRLTLCASHRLHAPQLSDEDNLRIYGKCNPPNGHGHNYEVKVTVYGEVDPVTGMVINAVDLKEYMQKAIMEPMDHKCV  84
Dre_PTPS  ------------------------------MSER-VAFITRVCSFSACHRLHSKCLSDEENKRTFGKCNNPNGHGHNYTVEVTVRGKIDKNTGMVMNLTDLKEFIEEAVMKPLDHKNL  87
Xla_PTPS  SSLCSTDSTESSGLAAVNILNMQNCDLITSHPEPRIIQISRSLSFSASHRLHSNSLSEEENKRIFGKCNNPNGHGHNYKAIVTVRGKIDPITGMVMNLTDLKRYMEECITVPLDHKNV 118
Hsa_PTPS  ------MSTEGGGRRCQ-------------------AQVSRRISFSASHRLYSKFLSDEENLKLFGKCNNPNGHGHNYKVVVTVHGEIDPATGMVMNLADLKKYMEEAIMQPLDHKNL  93
Nve_PTPS  ----------------------------MACNKRPFVYITRSETFSACHRLHSTKLTDQQNIELFGKCNNKNGHGHNYKVEVTVASTVDEDTGMVMNLVALKGKIEEVLS-ELDHKNL  89
Tad_PTPS  ----------------------------MAERKR-IGYIKRIESFSAAHRLHSPFFTDEENRALYGKCNHENGHGHNYKLEVTLKGEIDLKTGMVMNIADLKVIINEVIMKQLDHKNL  89
                                              : *   ::* ***:: :::: :*   :.***  :******   :.: . :   :*** :   **  :.  .   :**: :

Cel_PTPS  DKDVEFFKTTVSTSENVAIYMFEKLKSVMSN-PSVLYKVTIEETPKNIFTYKGC-------------------------- 140
Dme_PTPS  DKDVEYFANTPSTTENLAVYIWDNIRLQLKK-PELLYEVKIHETPKNIISYRGPYPLNGIYNPINKRIAHDSCTNISSDSD 168
Spu_PTPS  DKDVNYFRTNVSTVENIAIFIWDSMAKCLPD-PSLLYEVKVHETDKNIAVYRGE-------------------------- 135
Cin_PTPS  DKDIDYFKDKPSTAENIAIYIWQQLEPYMKNEKCSLYKVQLCETENIVTEYMGEEG------------------------ 140
Dre_PTPS  DLDVPYFADVVSTTENLSVFIWDGLQKLLP--HDSLYEIKVYETAKNIVIYRGE-------------------------- 139
Xla_PTPS  DKDVPYFRNVVSTVENITVFIWEELQKRLP--AGLLYEVCVHETDSNSAVYRGE-------------------------- 170
Hsa_PTPS  DMDVPYFADVVSTTENVAVYIWDNLQKVLP--VGVLYKVKVYETDNNIVVYKGE-------------------------- 145
Nve_PTPS  DLDVEYFQTVVSTAENIAVYIYNQLNRGLPD-G-ILYEVRVHETDKNVAFYRGQ-----IKETLN--------------- 147
Tad_PTPS  DKDVPYFANYPSTAENIAVYIWDCLIEHLP----ILYEVLLYETDKNVAIYRGE--------TNN--------------- 142
          * *: :*    ** **:::::: :   :      **:: : ** .    * *
```

Figure S3 The *ptps-1/* B0041.6 gene encodes 6-Pyruvoyl Tetrahydropterin Synthase (PTPS). (A) Physical map of *ptps-1* region with mutant alleles and gene model. Nature of *ptps-1* alleles is shown below and approximate locations indicated with arrow (point mutation) or red bar (deletion). Various EST, OST, and transcriptome sequencing support the gene model shown, and the predicted protein sequence. Image partly derived from WormBase genome browser editable SVG. (B) Alignments of *C. elegans* PTPS-1 predicted protein with PTPS proteins from other metazoans. Asterisks below alignment show 100% conserved amino acids, colon indicates conserved highly similar aa's, period indicates conserved weakly similar aa's. Location of *ptps-1* mutant allele *(e3042)* marked with red letter. Species abbreviations: Cel – *C. elegans*, Dme – *Drosophila melanogaster,* Spu – *Strongylocentrotus purpuratus*, Cin – *Ciona intestinalis*, Dre – *Danio rerio*, Xla – *Xenopus laevis*, Hsa – *Homo sapiens*, Nve – *Nematostella vectensis*, Tad – *Trichoplax adherens.*
