# Supplementary material for: Cuticle Integrity and Biogenic Amine Synthesis in Caenorhabditis elegans Require the Cofactor Tetrahydrobiopterin (BH4)
Source: Genetics. 2015 Mar 24;200(1):237–53. doi: 10.1534/genetics.114.174110 (PMC4423366; doi:10.1534/genetics.114.174110)
Supplement: Supporting Information [file supp_114.174110_FigureS4.pdf]

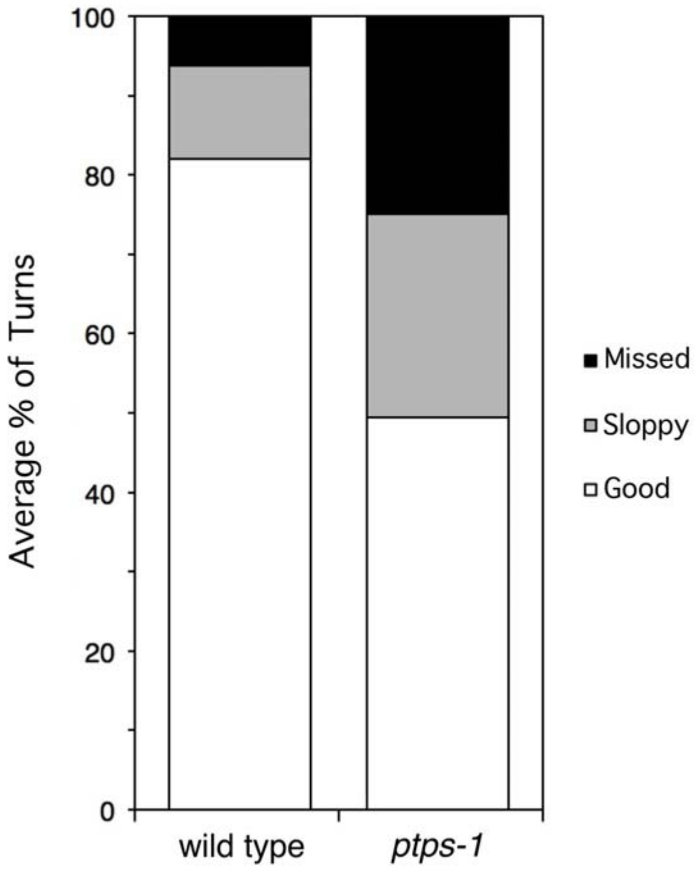

Figure S4 Male turning behavior is defective in *ptps-1* mutants. Columns show average percentage of each type of turn (good, sloppy, missed; definition of turn types from (Loer and Kenyon 1993) for each individual male, each observed for 5 min (wild type, n = 10 *him-5(e1490)* males; n = 12 *ptps-1(tm1984); him-5(e1490)* males).
