# Supplementary material for: Cuticle Integrity and Biogenic Amine Synthesis in Caenorhabditis elegans Require the Cofactor Tetrahydrobiopterin (BH4)
Source: Genetics. 2015 Mar 24;200(1):237–53. doi: 10.1534/genetics.114.174110 (PMC4423366; doi:10.1534/genetics.114.174110)
Supplement: Supporting Information [file supp_114.174110_FigureS5.pdf]

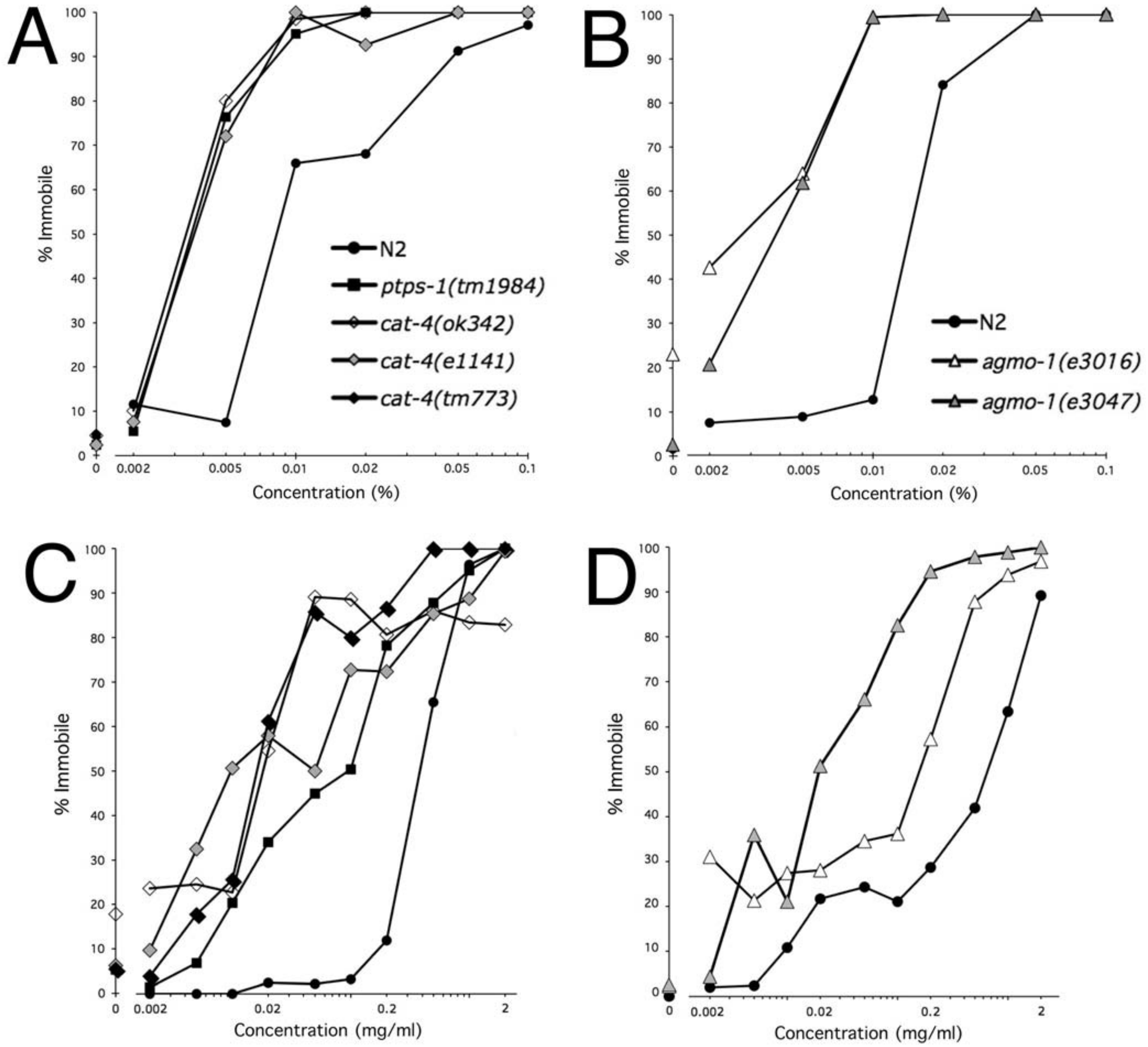

Figure S5 Similar hypersensitivity phenotypes of *cat-4*, *ptps-1* and *agmo-1* mutants. (A, B) SDS hypersensitivity of *cat-4, ptps-1* (A) and *agmo-1* (B) mutants. Synchronized adult gravid hermaphrodites were exposed to SDS in M9 buffer for 30 min, then examined for movement. Legend indicates alleles tested. (C, D) Levamisole hypersensitivity of *cat-4, ptps-1* (C) and *agmo-1* (D) mutants. Worms were exposed to levamisole in M9 for 5 min, then examined for movement. Legends as in A, B.
