# Supplementary material for: Cuticle Integrity and Biogenic Amine Synthesis in Caenorhabditis elegans Require the Cofactor Tetrahydrobiopterin (BH4)
Source: Genetics. 2015 Mar 24;200(1):237–53. doi: 10.1534/genetics.114.174110 (PMC4423366; doi:10.1534/genetics.114.174110)
Supplement: Supporting Information [file supp_114.174110_FigureS6.pdf]

```
      SL1 spliced leader
   1  ggtttaattacccaagtttgagaactcttctccacaaaattttaaccaaatgaaaagaggatgaacgagacggca  75
                                                                     M  N  E  T  A
                                                          ↓
  76  tggttggatagggtattctcaaatacgtcactgggacatcgattgctggatagactaactctcacgaatcttcgt  150
       W  L  D  R  V  F  S  N  T  S  L  G  H  R  L  L  D  R  L  T  L  T  N  L  R
 151  cacgcattctaccttatctcaccttacgagaccaccgtcgagtcgattgatgatgttccgaactataacgctgaa  225
       H  A  F  Y  L  I  S  P  Y  E  T  T  V  E  S  I  D  D  V  P  N  Y  N  A  E
 226  gtttctgcgtggtggctggtgtttcttactgccgagttttcatttattcatttccggacatgaagacagattt  300
       V  S  A  W  W  L  V  F  L  T  A  E  F  F  I  L  F  I  S  G  H  E  D  R  F
                                                            ↓
 301  gcactcaatgattcaataacgtcaatttgtgctggaatgctcagccaatgcttcaaatttggtggtcgagctgtc  375
       A  L  N  D  S  I  T  S  I  C  A  G  M  L  S  Q  C  F  K  F  G  G  R  A  V
 376  gcgatattcttgtacgtgattgtgtgggataactggcgaatattagaacctccatgggattccccgtggacatgg  450
       A  I  F  L  Y  V  I  V  W  D  N  W  R  I  L  E  P  P  W  D  S  P  W  T  W
                                                               ↓
 451  atttttgcctgttctttcaagattttatgtattatctggggcatcgggctgtgcatgaagccggcttcttctgg  525
       I  F  C  L  F  F  Q  D  F  M  Y  Y  L  G  H  R  A  V  H  E  A  G  F  F  W
 526  ggtcttcacacaattcaccatagctccgaatactacaatttctcaactgctctacgacaagctgccatacaagat  600
       G  L  H  T  I  H  H  S  S  E  Y  Y  N  F  S  T  A  L  R  Q  A  A  I  Q  D
 601  gctggattagcgatctatgactgtattcaggcattcttcatccctccatcaatattttagttcatcgatatttt  675
       A  G  L  A  I  Y  D  C  I  Q  A  F  F  I  P  P  S  I  F  L  V  H  R  Y  F
                                      ↓
 676  tcggagattttccaatttatcatgcatacctcgttggtggacaccatgggaccccttggcttagtattcaacact  750
       S  E  I  F  Q  F  I  M  H  T  S  L  V  D  T  M  G  P  L  G  L  V  F  N  T
 751  ccgtctcatcatcgagtacatcatggaagaaatccgtattgtattgacaagaattatggaggagttttcattatt  825
       P  S  H  H  R  V  H  H  G  R  N  P  Y  C  I  D  K  N  Y  G  G  V  F  I  I
              ↓
 826  tgggataagatgttcaacacatttgaagccgaacgtcacgatgacccaccaatctatggattagttaccaacgag  900
       W  D  K  M  F  N  T  F  E  A  E  R  H  D  D  P  P  I  Y  G  L  V  T  N  E
 901  aacactttcaatcaaatctacctccaattccatgctctttgggacattttaattttcaaaggattcacaaaagat  975
       N  T  F  N  Q  I  Y  L  Q  F  H  A  L  W  D  I  L  I  F  K  G  F  T  K  D
 976  gtgaaaggagagcccatgtttcctggagttgtgaacaaattgaaagcaaccgtattcccgccgggctggttccca  1050
       V  K  G  E  P  M  F  P  G  V  V  N  K  L  K  A  T  V  F  P  P  G  W  F  P
1051  ggggttcctgtcaccccgttctttcattggatgagcatggttaatccagctcacggagtacctgagccagagaaa  1125
       G  V  P  V  T  P  F  F  H  W  M  S  M  V  N  P  A  H  G  V  P  E  P  E  K
1126  cctgttctcagatacagcccacctgcgaggatcctagtgaaagtttacgtggcatcgtcattcttgttgttgttg  1200
       P  V  L  R  Y  S  P  P  A  R  I  L  V  K  V  Y  V  A  S  S  F  L  L  L  L
1201  gctatattcttccattttgaatacgaccggaatcatttgagctacttggattgtacagtcaagattgcatacttt  1275
       A  I  F  F  H  F  E  Y  D  R  N  H  L  S  Y  L  D  C  T  V  K  I  A  Y  F
                                               ↓
1276  gtggttacgatgcaatgttttggagcatttttgatatgaaatggtatgcccggtacattgaaattgctcgttgt  1350
       V  V  T  M  Q  C  F  G  A  F  F  D  M  K  W  Y  A  R  Y  I  E  I  A  R  C
1351  tgtggagttctcatctattacggagtactcatgttcgatcatattggtgcaggaactcatcgtcttttgtcatt  1425
       C  G  V  L  I  Y  Y  G  V  L  M  F  D  H  I  G  A  G  T  H  R  L  F  V  I
                                                        ↓
1426  tcactgcatatcatggctattgcattgtggacgactgatgttttggtggagaaactctcccaatgctgctcaaag  1500
       S  L  H  I  M  A  I  A  L  W  T  T  D  V  L  V  E  K  L  S  Q  C  C  S  K
1501  aatcaatcaataaatccagaaaaaggtgacctggaacgggctccagaaattgcatcgatctcgaaaaatgttcaa  1575
       N  Q  S  I  N  P  E  K  G  D  L  E  R  A  P  E  I  A  S  I  S  K  N  V  Q
1576  taatatgattttatagagttgttgtttccattgtctttccaggctgtcatattctagtcactcttttcccttttt  1650
       *
1651  ttgtttgatggttttcttaaattaaagatttttctctcaaaaaa                                1725
```

Figure S6 An *agmo-1* cDNA encodes a 505 amino acid protein. For *agmo-1*, we completed sequencing of cDNA yk1597b01 (previously partially sequenced, accession BJ763208) kindly provided by Yuji Kohara. The clone had a single nucleotide difference from genomic sequence that would result in a single amino acid difference from that predicted from genomic sequence (nt: T425C; aa: L122P) Both differences are marked in red. Introns located following the base indicated by a red arrow. The trans-spliced leader SL1 is underlined at the 5' end; the likely polyadenylation signal is underlined in the 3' UTR. We also sequenced twelve independent ORFeome clones (Reboul et al. 2001). We found clones of two different lengths, but both types appeared to be abnormal with an unspliced intron #4 which would result in a severely truncated protein. Genbank accession number for this *agmo-1* cDNA clone is KP290894.
