# Supplementary material for: Cuticle Integrity and Biogenic Amine Synthesis in Caenorhabditis elegans Require the Cofactor Tetrahydrobiopterin (BH4)
Source: Genetics. 2015 Mar 24;200(1):237–53. doi: 10.1534/genetics.114.174110 (PMC4423366; doi:10.1534/genetics.114.174110)
Supplement: Supporting Information [file supp_114.174110_FigureS7.pdf]

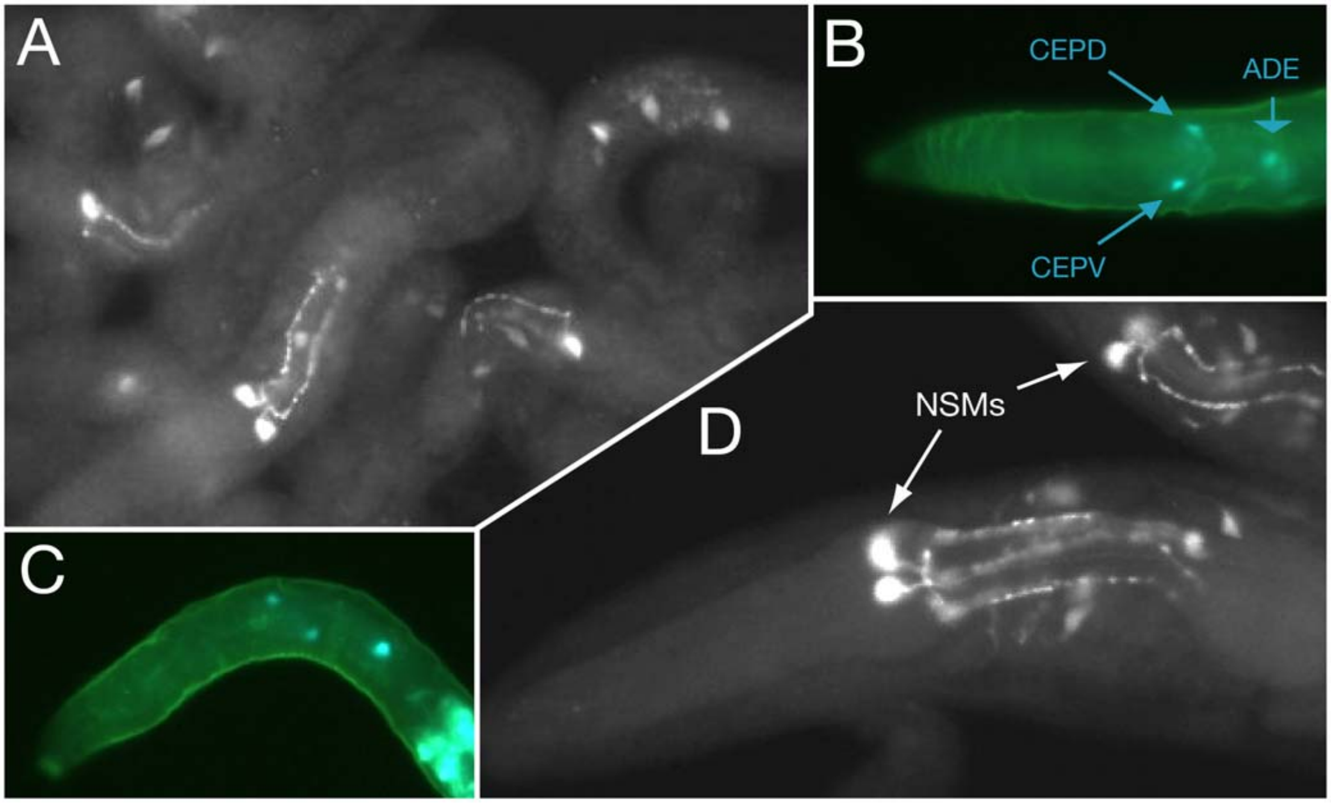

Figure S7 *agmo-1* mutants have normal 5HT and DA**.** (A, B) *agmo-1(e3047);* (C, D) *agmo-1(e3016).* Anti-5HT immunofluorescence (A, D) of worm heads of larvae (A, D) and adult (D) showing normal 5HT neurons. FIF (B, C) of larval heads showing normal DA neuron staining.
