# Supplementary material for: Cuticle Integrity and Biogenic Amine Synthesis in Caenorhabditis elegans Require the Cofactor Tetrahydrobiopterin (BH4)
Source: Genetics. 2015 Mar 24;200(1):237–53. doi: 10.1534/genetics.114.174110 (PMC4423366; doi:10.1534/genetics.114.174110)
Supplement: Supporting Information [file supp_114.174110_FigureS8.pdf]

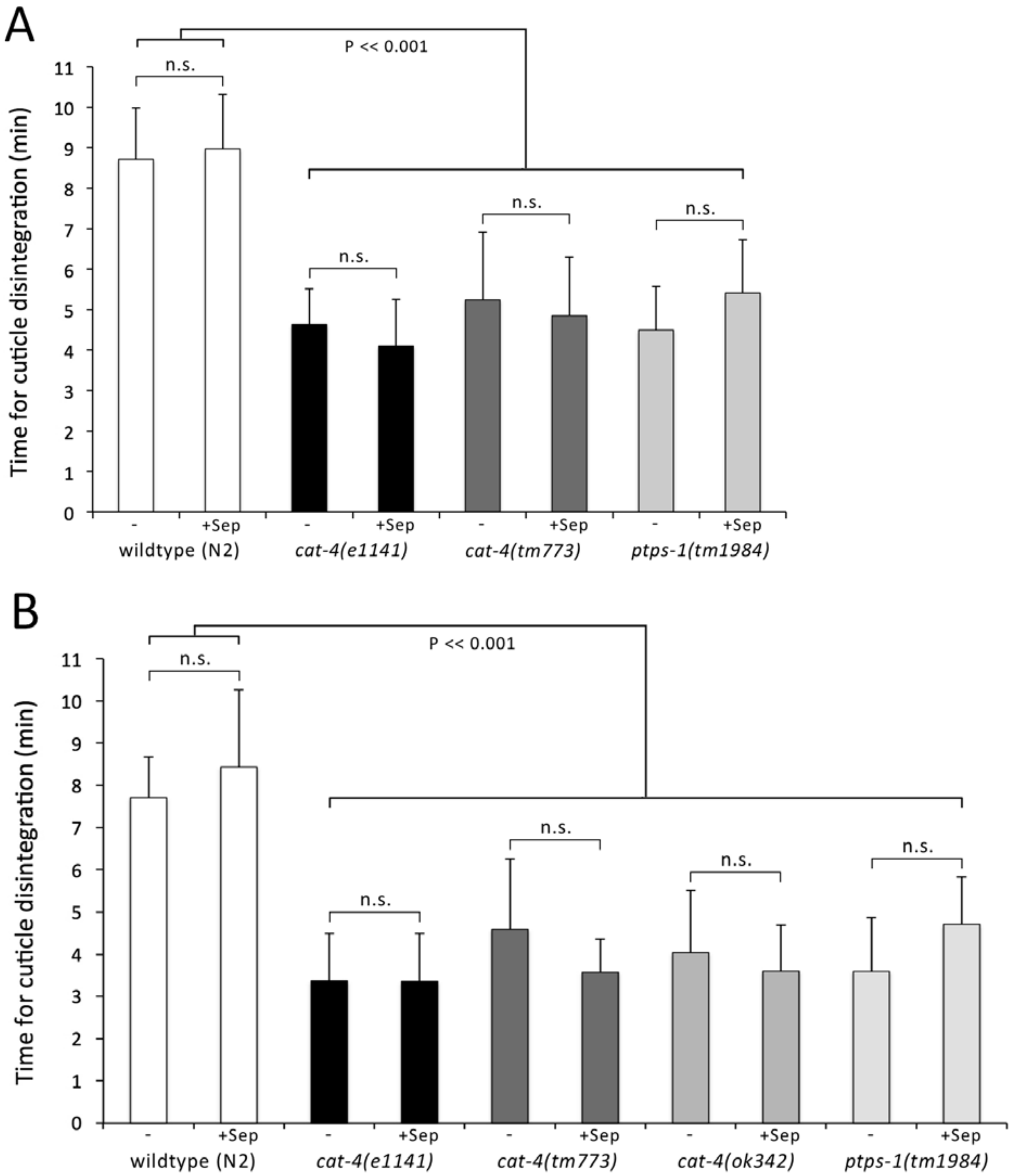

Figure S8 Exogenous biopterins do not rescue cuticle fragility in BH4-deficient mutants. (A-E) Cuticle disintegration time (mean ± S.D.) during mild alkaline bleach treatment, scored as first major cuticle rupture; worms from mixed stage cultures (n = 15), as in Fig 4 (B, C). For all experiments, groups were compared with 1-factor ANOVA followed by planned pairwise comparisons made with Scheffè's F-test (Sokal and Rohlf 1981). (A) Wild-type worms compared to biopterin deficient worms, with or without supplemental sepiapterin (100 µM). There were significant differences among the groups (overall ANOVA, P<< 0.0001). All pairwise comparisons between a genotype with sepiapterin (+Sep) or without (-) were not significant (n.s.). All pairwise comparisons of wild type with mutants were significantly different (P<<0.001). No biopterin deficient genotype was significantly different from another. (B) Additional experiment comparing wild-type to biopterin deficient worms, with or without supplemental sepiapterin, including *cat-4(ok342)*. Results like in (A). A customary approach in mammalian cells to increase BH4 intracellularly is to use sepiapterin (Werner-Felmayer et al. 2002; Aguado et al. 2006), since SR can effectively convert sepiapterin to 7,8 dihydrobiopterin (BH2) intracellularly, which is then reduced to BH4 by dihydrofolate reductase (DHFR). In *C. elegans,* we believe that other reductases substitute for SR and carry out the last steps in the synthesis of BH4 (since there is no ortholog); these enzymes should also be able to convert sepiapterin to BH2, the latter being further reduced to BH4 by DHFR in the so-called salvage pathway (Thöny et al. 2000).

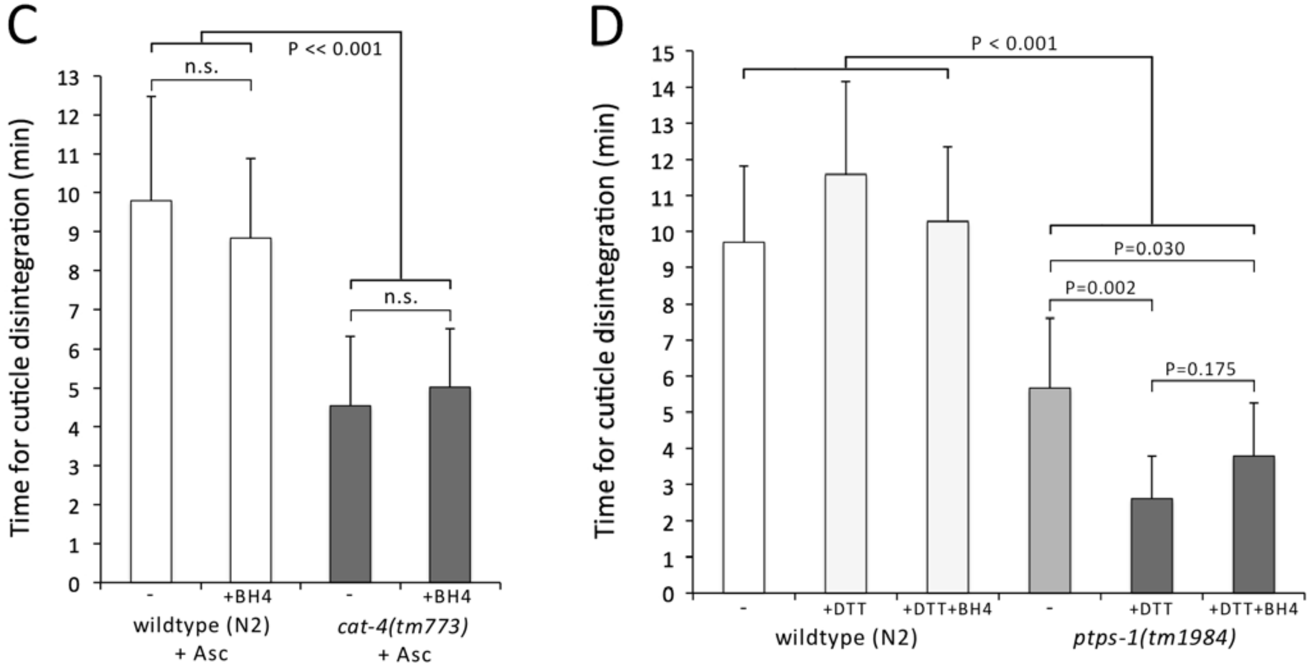

Figure S8 Exogenous biopterins do not rescue cuticle fragility in BH4-deficient mutants. (C) Supplementation with BH4 (200 µM), including ascorbate (Asc, 5 mM added to reduce oxidation) does not alter cuticle fragility in wild type or *cat-4(tm773)* mutant. Comparisons as in (A). (D) Supplementation with BH4 (200 µM) with or without dithiothreitol (DTT, 5 mm added to reduce oxidation) does not alter cuticle fragility in wild type (left columns, all N2 group comparisons not significant). Addition of DTT makes *ptps-1(tm1984)* mutant cuticles significantly more fragile (P<0.05 in pairwise comparisons with or without DTT). BH4 may reduce damage caused by DTT although the difference is not statistically significant (P=0.175). DTT can reduce disulfide bonds in the highly cross-linked *C. elegans* cuticle (Stenvall et al. 2011).

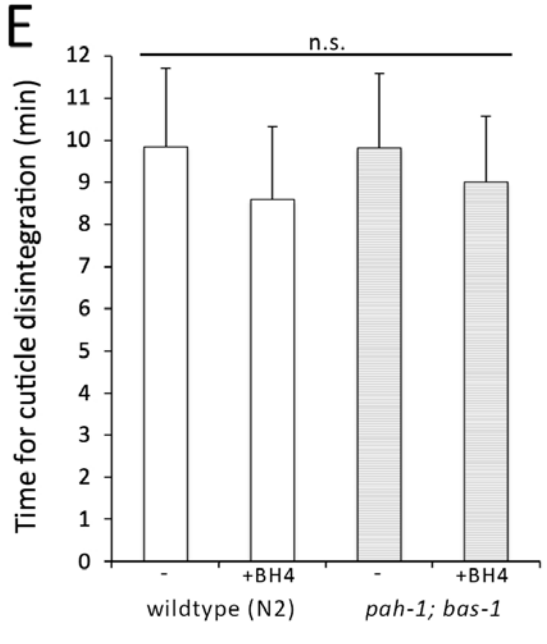

Figure S8 Exogenous biopterins do not rescue cuticle fragility in BH4-deficient mutants. (E) Loss of PAH activity, 5HT and DA do not affect cuticle fragility; supplementation with BH4 does not affect cuticle fragility of wild type or *pah-1; bas-1* mutant. There were no significant differences among the groups in the overall 1-factor ANOVA. Double mutant *pah-1(tm520); bas-1(ad446)* lacks PAH and aromatic amino acid decarboxylase activities; *bas-1* mutants are 5HT- and DA-deficient (Hare and Loer 2004; Calvo et al. 2008).
