# Supplementary material for: Cuticle Integrity and Biogenic Amine Synthesis in Caenorhabditis elegans Require the Cofactor Tetrahydrobiopterin (BH4)
Source: Genetics. 2015 Mar 24;200(1):237–53. doi: 10.1534/genetics.114.174110 (PMC4423366; doi:10.1534/genetics.114.174110)
Supplement: Supporting Information [file supp_114.174110_FigureS9.pdf]

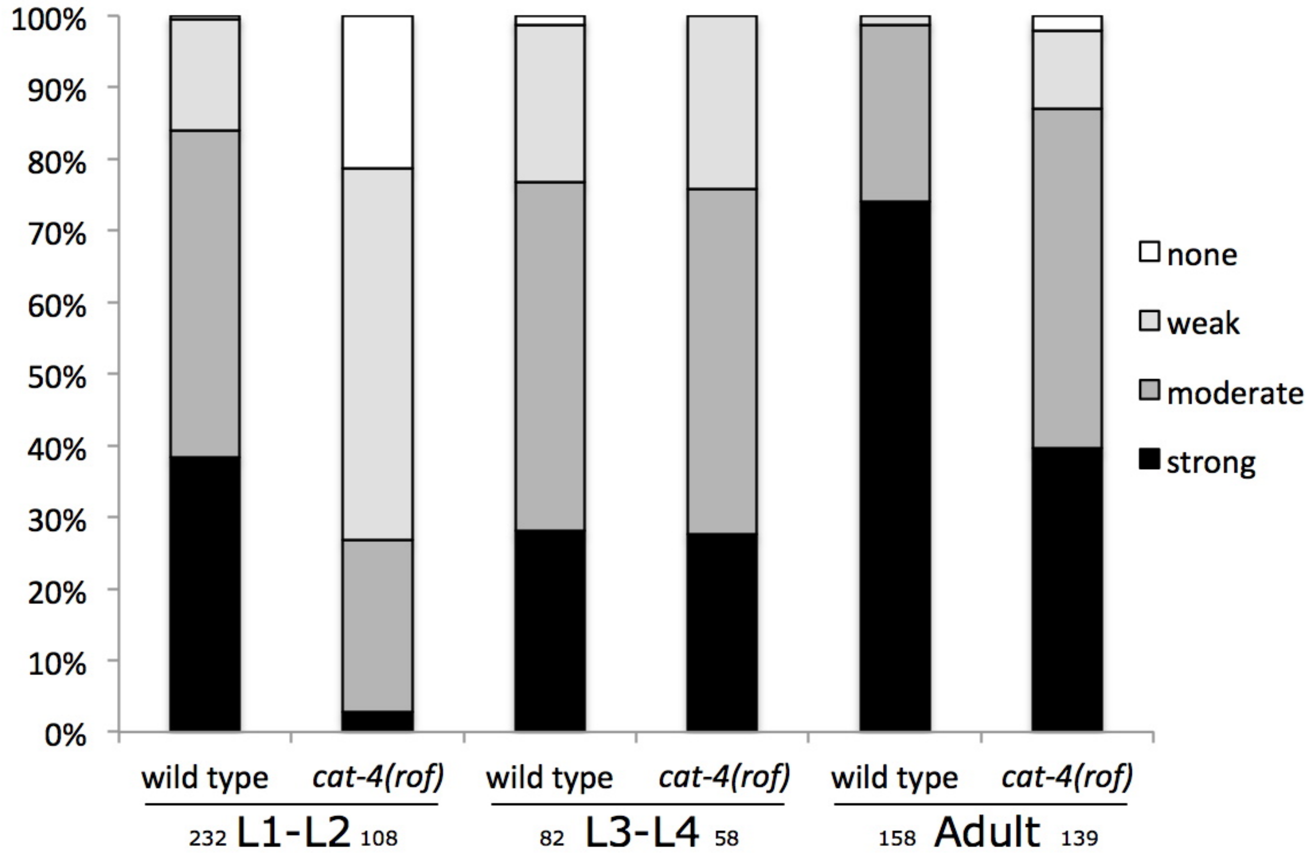

Figure S9 *cat-4* reduction-of-function mutants accumulate 5HT during larval development. Anti-5HT immunoreactivity differences in wild type and *cat-4(e3015)* reduction of function *(rof)* are most apparent in young larvae. 85% of wild-type worms are strongly to moderately stained whereas only 27% of *cat-4(e3015)* L1-L2 worms are strongly to moderately stained. Mutants in mixed populations of young (L1-L2), older larvae (L3-L4), and adults, scored for intensity of NSM cell body staining (as in Fig. 7A, B). Numbers of worms scored shown below the column.
