# Supplementary material for: Cuticle Integrity and Biogenic Amine Synthesis in Caenorhabditis elegans Require the Cofactor Tetrahydrobiopterin (BH4)
Source: Genetics. 2015 Mar 24;200(1):237–53. doi: 10.1534/genetics.114.174110 (PMC4423366; doi:10.1534/genetics.114.174110)
Supplement: Supporting Information [file supp_114.174110_FigureS10.pdf]

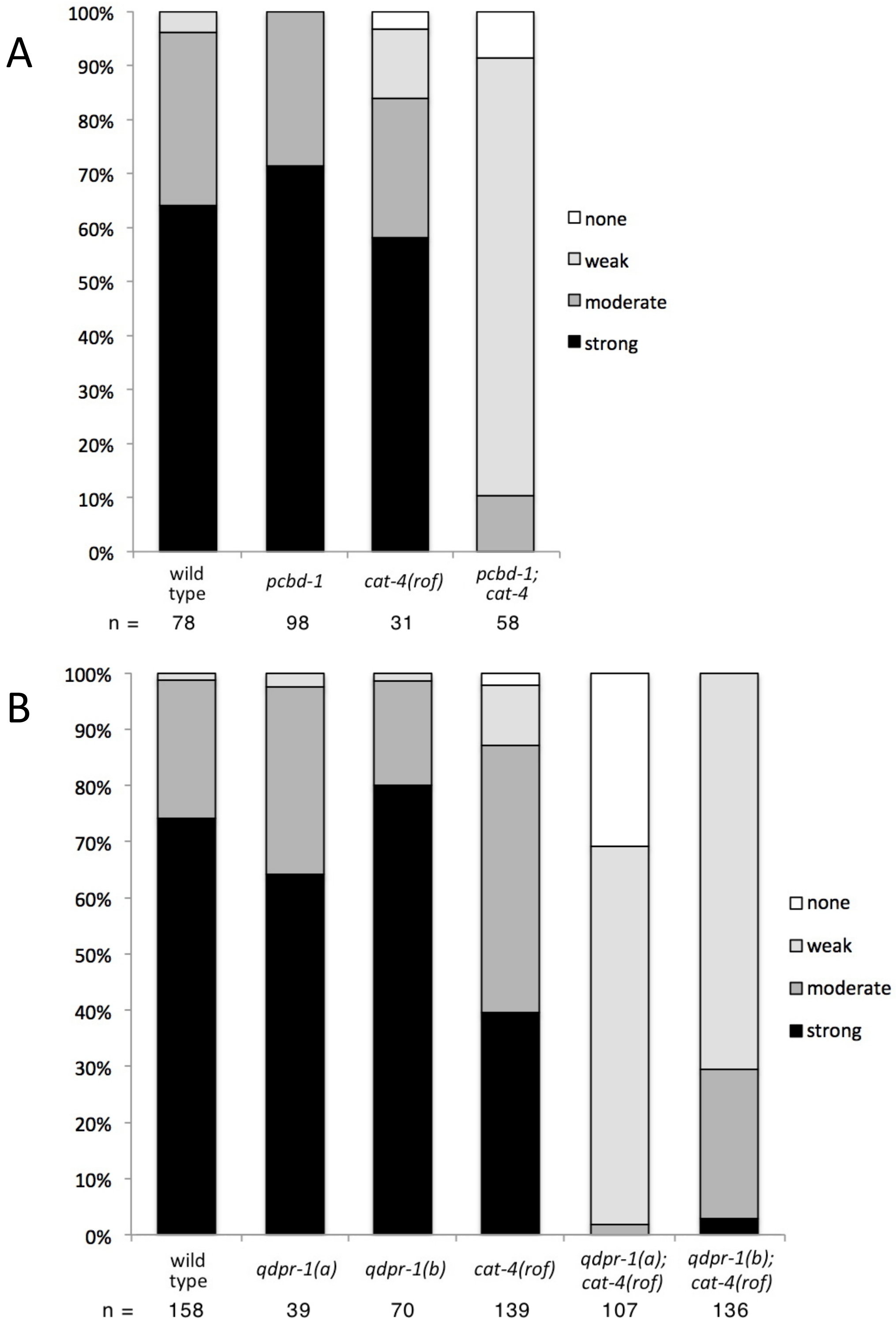

Figure S10 Biopterin regeneration genes *pcbd-1* and *qdpr-1* function in 5HT synthesis in adult worms. Anti-5HT immunoreactivity differences in wild type, single mutant, and double mutants in adult worms (see legend for Fig 7A, B). Numbers of worm scored shown below the columns. (A) *pcbd-1* single and double mutants with *cat-4(e3015)*. (B) *qdpr-1* single and double mutants with *cat-4(e3015).*
