# Supplementary material for: Cuticle Integrity and Biogenic Amine Synthesis in Caenorhabditis elegans Require the Cofactor Tetrahydrobiopterin (BH4)
Source: Genetics. 2015 Mar 24;200(1):237–53. doi: 10.1534/genetics.114.174110 (PMC4423366; doi:10.1534/genetics.114.174110)
Supplement: Supporting Information [file supp_114.174110_FigureS11.pdf]

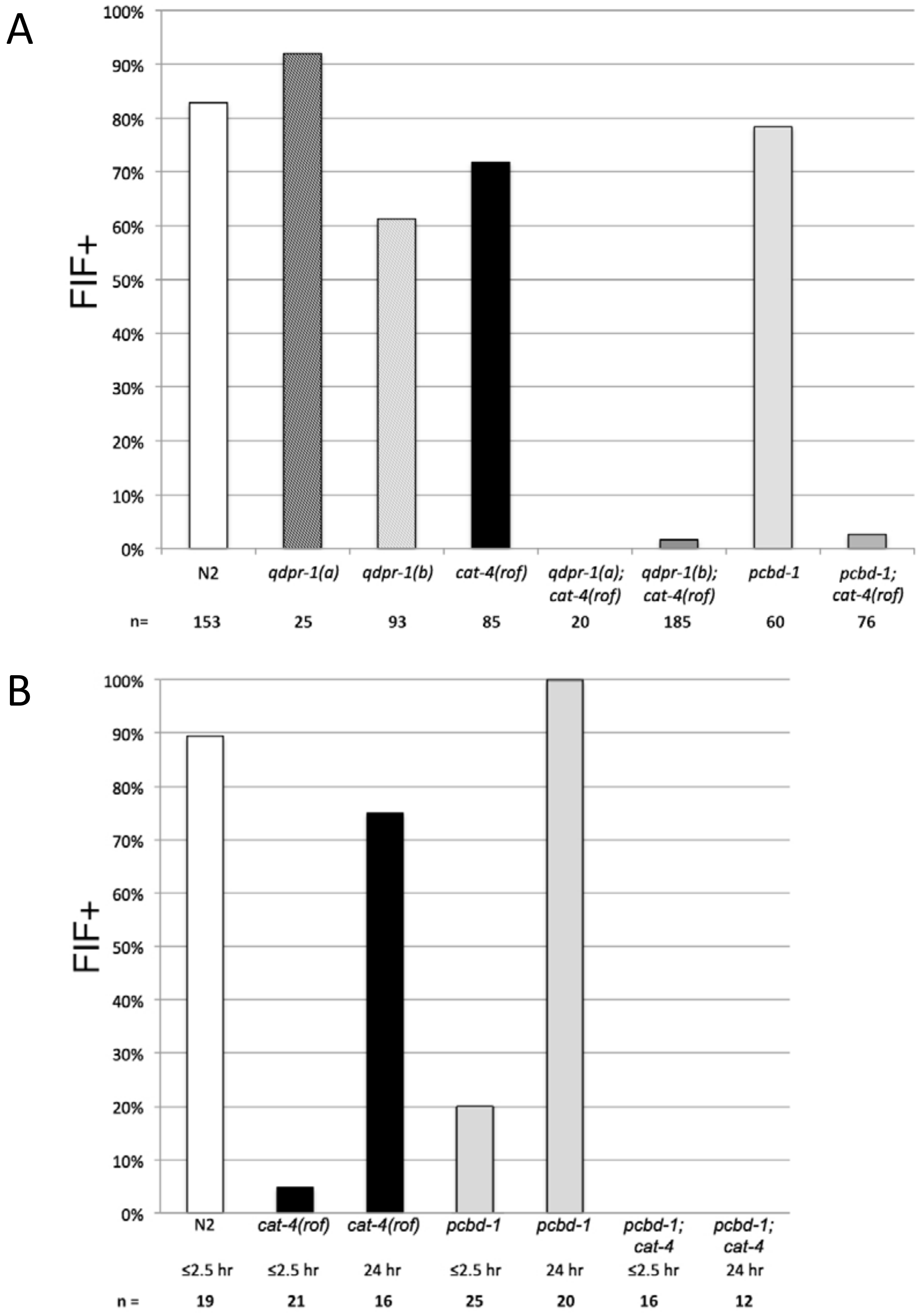

Figure S11 Biopterin regeneration genes *pcbd-1* and *qdpr-1* function in DA synthesis. (A) Mixed populations, young worms (L1-L2) scored for DA by formaldehyde induced fluorescence (FIF). Y axis – percentage of worms with one or more FIF+ head neurons (without regard to brightness). Alleles used: *cat-4(rof)* = 'reduction of function' = *e3015*; *qdpr-1(a) = tm2337; qdpr-1(b) = tm2373; pcbd-1(tm5924).* (B) Loss of DA in BH4 synthesis and regeneration mutants is most apparent in very young worms. Staged worms scored for DA by FIF – early L1s (≤ 2.5 hr post-hatching), mid-L2s (~ 24 post-hatching). Y axis and alleles used as in (A). Eggs were picked to a seeded plate and allowed to hatch for 2.5 hr, at which time hatchling worms were immediately subjected to FIF staining, or transferred to a new seeded plate, incubated at 20°, and tested by FIF 24 hr later.
