# Supplementary material for: Cuticle Integrity and Biogenic Amine Synthesis in Caenorhabditis elegans Require the Cofactor Tetrahydrobiopterin (BH4)
Source: Genetics. 2015 Mar 24;200(1):237–53. doi: 10.1534/genetics.114.174110 (PMC4423366; doi:10.1534/genetics.114.174110)
Supplement: Supporting Information [file supp_114.174110_FigureS12.pdf]

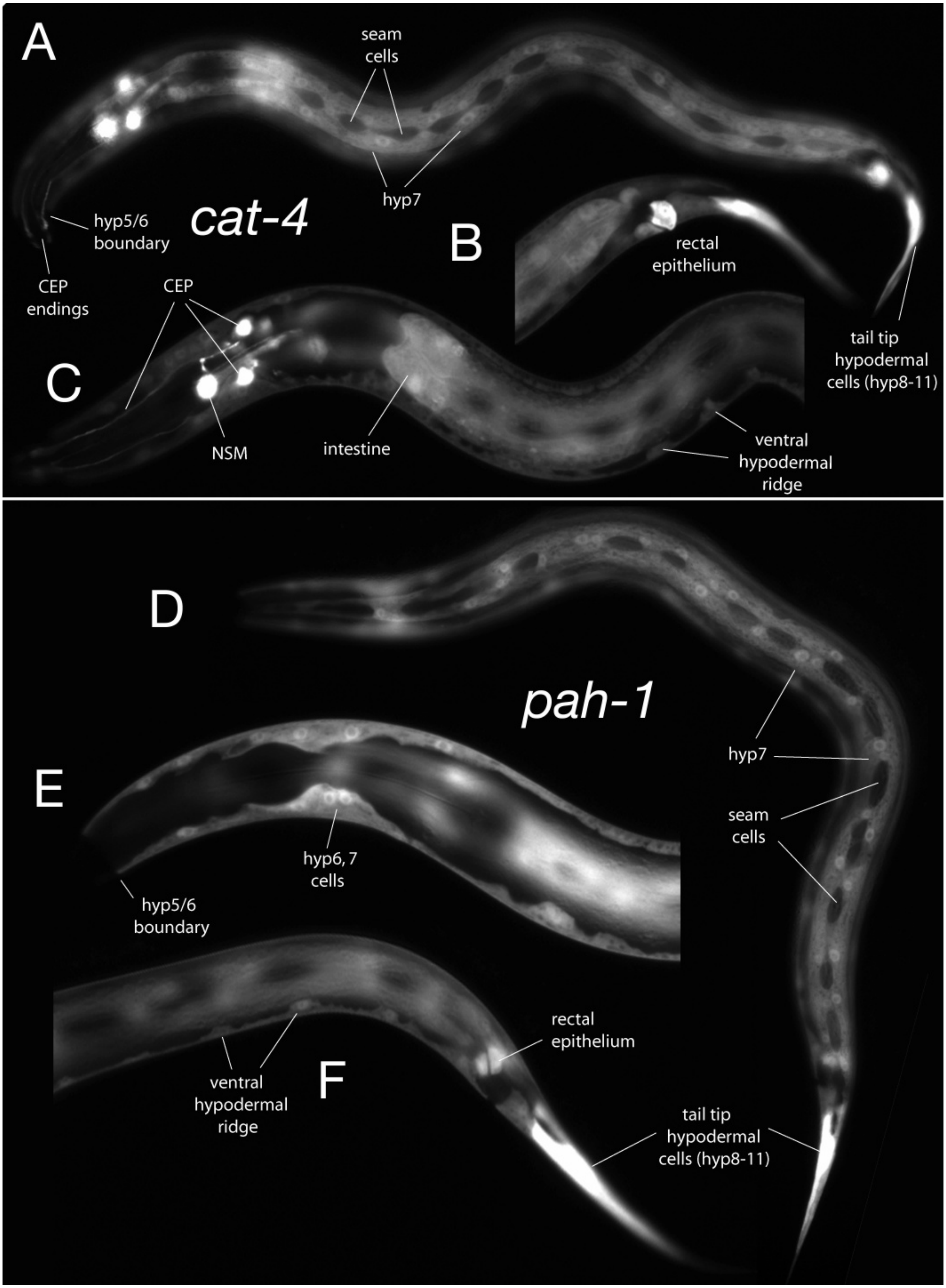
A
seam
cells
hyp7
hyp5/6
boundary
cat-4
CEP
endings
B
rectal
epithelium
CEP
C
NSM
intestine
tail tip
hypodermal
cells (hyp8-11)
ventral
hypodermal
ridge
D
pah-1
hyp7
E
seam
cells
hyp6, 7
cells
hyp5/6
boundary
rectal
epithelium
F
ventral
hypodermal
ridge
tail tip
hypodermal
cells (hyp8-11)

Figure S12 Expression of *cat-4* and *pah-1* GFP reporters. Anterior is to the left in all worms. (A-C) *cat-4*::GFP transgenic L2 stage worms, repeated from Fig 8A to facilitate comparison with *pah-1*::GFP expression (D-F). (A) Superficial focal plane showing epidermal expression, especially in hyp7 syncytium. Seam cells have undergone doubling division and can be seen along the lateral side as darker regions among the brightly staining hyp7 cell. Dendritic endings of CEP neurons can be seen at the tip of the 'nose.' (B) Medial focal plane showing anal cells, strongly expressing tail epidermal cells and posterior intestinal cells expressing GFP. (C) Medial focal plane showing epidermal expression in the body and head, and the boundary between hyp6 and unstained hyp5. NSM and CEP neuron somas are seen in the head, plus some neuronal processes (especially CEP processes). A few other neuronal somas stain less brightly. The anteriormost intestine also shows GFP expression, as do some rectal epithelial cells (here in what appear to be B & Y cells). (D-F) *pah-1*::GFP transgenic L2 stage worms. (D) Superficial focal plane showing especially hyp7 expression, with dark seam cells (post-doubling division) lying over hyp7. (E) Medial focal plane showing anterior epidermal expression. The hyp6 (expressing) / hyp5 (dark, not expressing) boundary is very clear here. (E) Medial focal plane in the posterior showing intestine, some rectal epithelial cells, and strongly expressing epidermal tail cells. (F) Medial focal plane showing ventral hypodermal ridge, rectal epithelium cells, and strongly expressing tail epidermal cells expressing GFP.

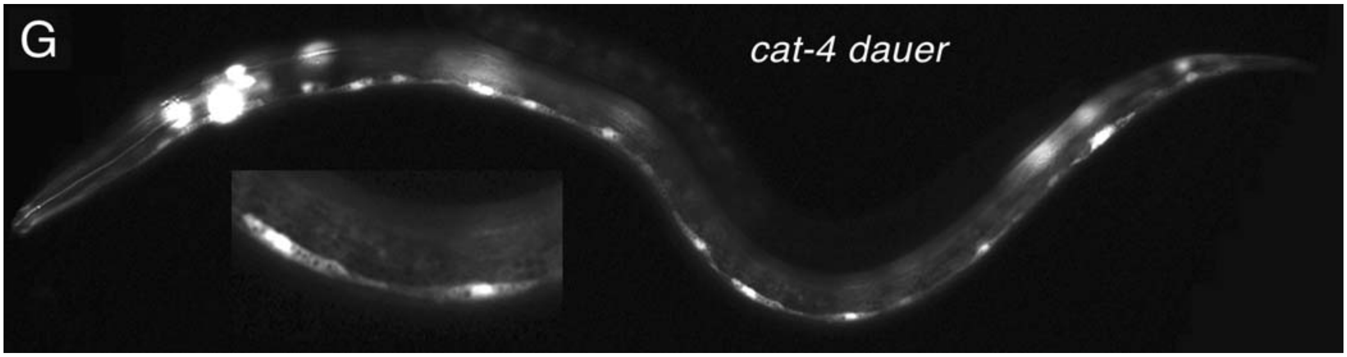

Figure S12 (G) *cat-4*::GFP worm showing expression in lateral epidermal seam cells during dauer stage. Anterior to the left. Inset: closeup of two seam cells in midbody region.
