# Supplementary material for: Cuticle Integrity and Biogenic Amine Synthesis in Caenorhabditis elegans Require the Cofactor Tetrahydrobiopterin (BH4)
Source: Genetics. 2015 Mar 24;200(1):237–53. doi: 10.1534/genetics.114.174110 (PMC4423366; doi:10.1534/genetics.114.174110)
Supplement: Supporting Information [file supp_114.174110_FigureS13.pdf]

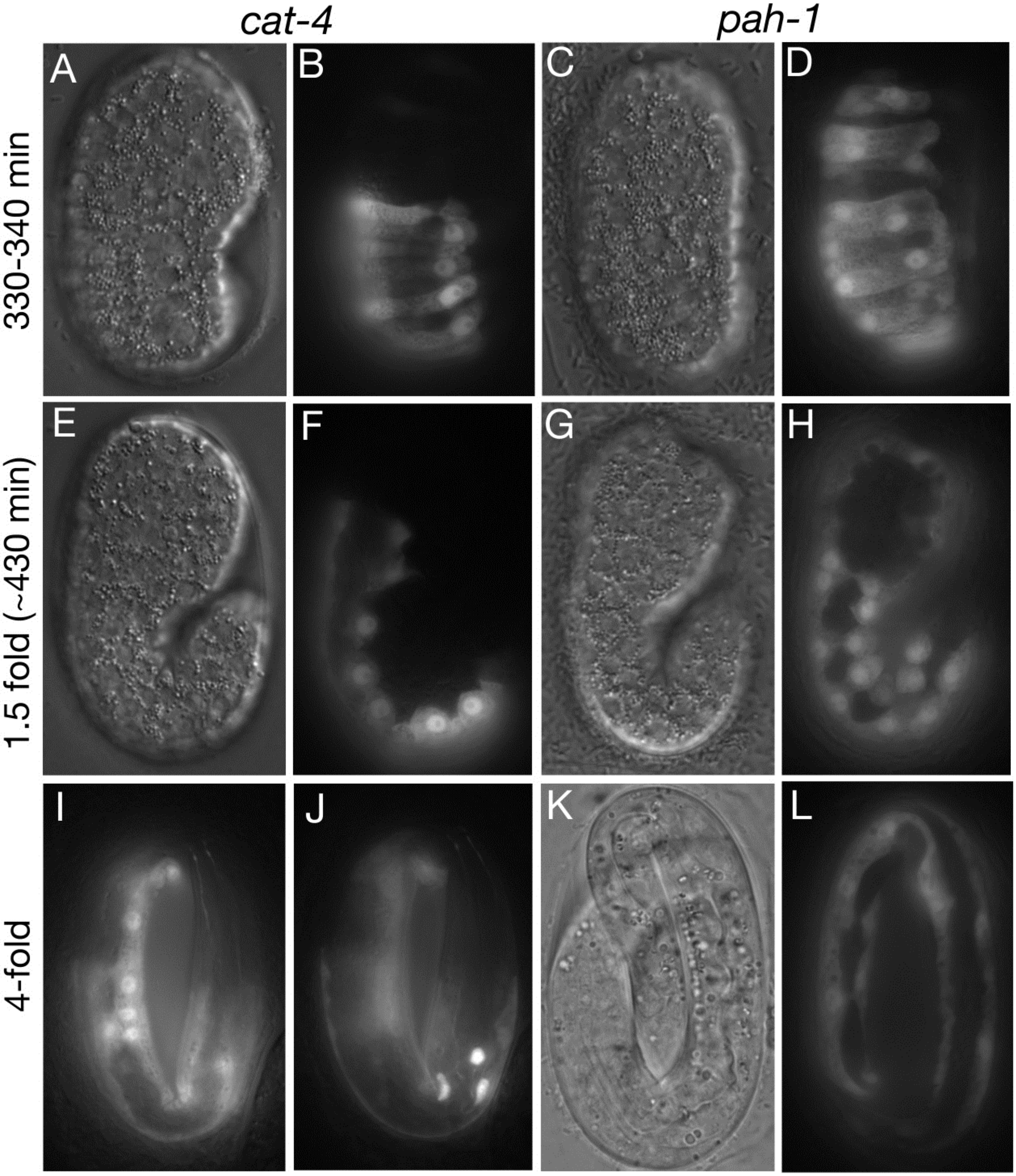

Figure S13 Expression of *cat-4*, *pah-1* and *ptps-1* reporter constructs in embryos. Left hand columns (A, B, E, F, I, J) - *cat-4*::GFP embryos; Right hand columns (C, D, G, H, K, L) *pah-1*::GFP embryos. Pairs of photos (e.g., A+B) have DIC view of embryos (A) matched with GFP fluorescence (B) in same superficial focal plane. Top row (A-D): 330-340 min, post nuclear counter-migration. Embryos have just begun elongation, dorsal view, anterior up. (B) *cat-4*::GFP embryo shows expression in posterior dorsal epidermal cells. (D) *pah-1*::GFP is expressed in both anterior and posterior dorsal epidermal cells. Middle row (E-H). 1.5-fold stage, lateral view. (F) *cat-4*::GFP expression is seen in posterior dorsal epidermal cells. (H) *pah-1*::GFP is expressed in most dorsal and ventral epidermal cells, but not in lateral seam cells. Bottom row (I-L). Late (4-fold) embryos. (I) Superficial focal plane of anterior of worm showing *cat-4*::GFP epidermal staining (except in seam cells) similar to that seen in larvae. CEP neurons dendritic endings are seen in tip of nose. (J) Deeper focal plane of same worm showing NSM and CEP neurons expressing GFP. (K) DIC view of *pah-1*::GFP late embryo (4-fold). (L) Different, superficial focal plane of same worm.

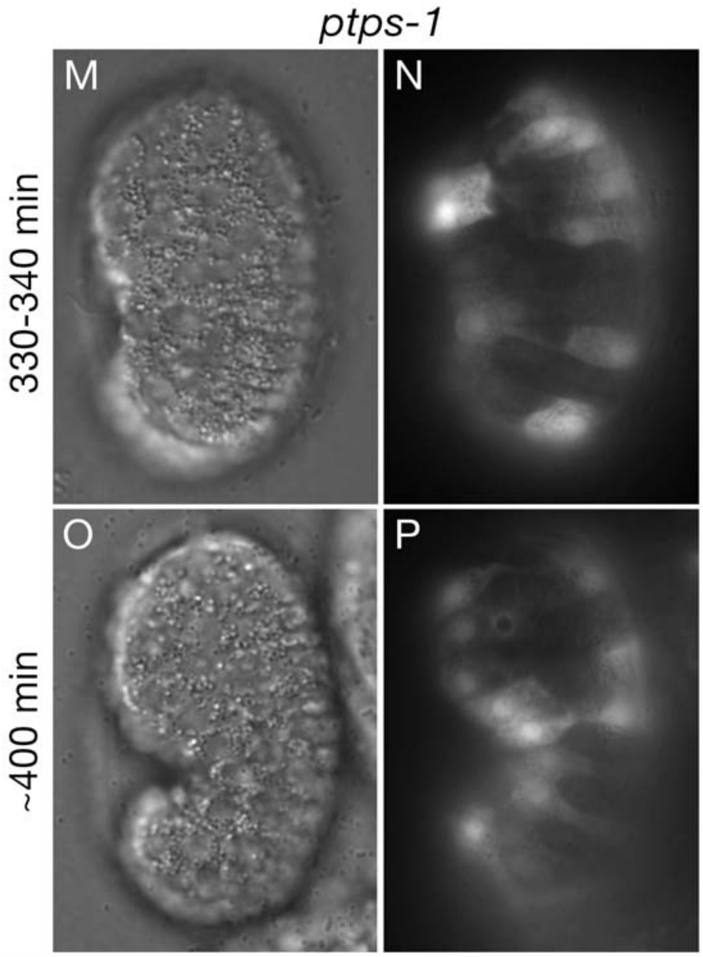

Figure S13 Expression of *cat-4*, *pah-1* and *ptps-1* reporter constructs in embryos. (M-P) *ptps-1*::GFP embryos (strain OH11619). Pairs of matching photos showing DIC view of embryo with GFP fluorescence at the indicated times. Expression seen in both anterior and posterior dorsal epidermal cells
