# Supplementary material for: Cuticle Integrity and Biogenic Amine Synthesis in Caenorhabditis elegans Require the Cofactor Tetrahydrobiopterin (BH4)
Source: Genetics. 2015 Mar 24;200(1):237–53. doi: 10.1534/genetics.114.174110 (PMC4423366; doi:10.1534/genetics.114.174110)
Supplement: Supporting Information [file supp_114.174110_FileS1.pdf]

File S1

Supporting Materials and Methods

Worms strains used in this study:

N2 wild type
CB369 *unc-51(e369)*
HA1335 *cat-4(ok342)V*
VC20144 *cat-4(gk245686)V*
CLB49 [line 4] *glsEx3 [rol-6(dom) cat-4(+)]; cat-4(tm773)V*
CB7130 *him-8(e1489)IV; cat-4(e3030)V*
LC84 *ptps-1(tm1984)I; him-5(e1490)V*
LC87 *qdpr-1(tm2337)III*
LC133 *pcbd-1(tm5924)I*
LC130 *qdpr-1(tm2373)III; cat-4(e3015)V*
LC83 *pah-1(tm520); bas-1(ad446)*
CB7127 *agmo-1(e3019)III*
CB7129 *agmo-1(e3047)III*
CB1490 *him-5(e1490)V*
CB1141 *cat-4(e1141)V*
LC81 *cat-4(tm773)V*
CLB49 [line 1] *glsEx1 [rol-6(dom) cat-4(+)]; cat-4(tm773)V*
CB7107 *him-8(e1489)IV; cat-4(e3015)V*
LC80 *ptps-1(tm1984)I*
CB7094 *ptps-1(e3042)I*
LC90 *qdpr-1(tm2373)III*
LC129 *qdpr-1(tm2337)III; cat-4(e3015)V*
LC131 *pcbd-1(tm5924)I; cat-4(e3015)V*
CB7014 *agmo-1(e3016)III*
CB7128 *agmo-1(e3029)III*
VC1198 *Y39G8B.1(ok1682)II*

See also transgenic reporter fusion strains listed in Tables S1, S2.
