# Supplementary material for: Cuticle Integrity and Biogenic Amine Synthesis in Caenorhabditis elegans Require the Cofactor Tetrahydrobiopterin (BH4)
Source: Genetics. 2015 Mar 24;200(1):237–53. doi: 10.1534/genetics.114.174110 (PMC4423366; doi:10.1534/genetics.114.174110)
Supplement: Supporting Information [file supp_114.174110_TableS1.pdf]

Table S1 Biopterin-related GFP reporter gene transgenics (this work) – primers and characteristics

Gene	Primers†	Sequence	Fusion type	Size (bp)**	Transgenics ‡
cat-4	AC1763 (A)<br>AC1764 (A*)<br>AC1765 (B)	AAAGGTTGCATGTTGCAGATG<br>TGCATGTTGCAGATGGAAATTG<br>CATTTTGATATTATGATGTTGATAGA	Transcriptional, through ATG	2654	CZ9718, -19 *(juEx2054, -5)*
ptps-1	AC3051 (A)<br>AC3050 (B)	ATAGTCCGGTCTTGTACCAC<br>ACATCCTTTATAAGTGAAAATATT	Translational, in final coding exon	2751 (~2000)	CZ18320, -21, -22 *(juEx5474, -5, -6)*
	AC3049 (A)§	TCGAATTTTCGCGGACAAGG	Translational, in final coding exon	1160 (~450)	CZ18092, -93 *(juEx5477, -8)*
pcbd-1	AC3217 (A)<br>AC3218 (B)	ATCAGAGTAGGAGTCAGGGAG<br>TGAATGAGAAGATGCGTTGAGAAG	Transcriptional, upstream of ATG	1505	CZ19212 *(juEx5785)*
qdpr-1	AC3219 (A)<br>AC3220 (B)	CGGTATCCTTGTCGCCAAAC<br>TGTTCGAAACTGCAAGGAAAAAGT	Transcriptional, upstream of ATG	1048	CZ19213, -14 *(juEx5786, -7)*
qdpr-1	qdpr1AL1 (A)<br>qdpr1AL2 (A*)<br>qdpr1BR (B)	TGGCGGAATCGATTTATTTG<br>GCCATATTGCGTTCAATGAG<br>TCGATGTTCCATTTTCAGTGG	Translational, in final coding exon	4887 (~4000)	CZ19215 *(juEx5788)*
gfrp-1	AC3221 (A)<br>AC3222 (B)	AATACGGTGCCAGGTGTCAG<br>TTCTTGATTTTTTTGTTGCGGAAT	Transcriptional, upstream of ATG	1088	CZ19216 *(juEx5789)*
agmo-1	AC3030 (A)<br>AC3031 (B)	ACTTGGCAAACAGTTGGAAGC<br>CCTCTTTTCATTTGGTTAAAATTT	Transcriptional, upstream of ATG	2281	CZ17563, -64, -65 *(juEx5238, -39, -40)*
pah-1	AC1769 (A)<br>AC1770 (A*)<br>AC1771 (B)	ATGGAAGGTCAGATTGGATATC<br>TCAGATTGGATATCTTCCACG<br>CATTTTCAGAGAACAAGATTTGGT	Transcriptional, through ATG of an isoform	2005	CZ9720, -21 *juEx2056, -7*

† - Primer name ('style' of primer, ala Hobert, 2002); not shown above, all 'B' primers begin with: AGTCGACCTGCAGGCATGCAAGCT (sequence overlapping GFP coding to create fusion)

§ - Primer paired with the same B (AC3050)

** - Length of sequence upstream to GFP (length upstream of predicted translation start if translational fusion)

‡ - Strain number (beginning with CZ) and allele designation of extrachromosomal transgenes (*juEx*)
