# Supplementary material for: Cuticle Integrity and Biogenic Amine Synthesis in Caenorhabditis elegans Require the Cofactor Tetrahydrobiopterin (BH4)
Source: Genetics. 2015 Mar 24;200(1):237–53. doi: 10.1534/genetics.114.174110 (PMC4423366; doi:10.1534/genetics.114.174110)
Supplement: Supporting Information [file supp_114.174110_TableS2.pdf]

Table S2 Biopterin-related GFP reporter gene transgenics from other sources

Gene	Fusion type	Size (bp)**	Transgenic	Reference
cat-4	translational, exon 2	~2800 (~2000)	JY739†	Sze et al. 2002†
cat-4	transcriptional	~630	OH8482	Flames and Hobert 2009
ptps-1	transcriptional	~2600	OH11619	Zhang et al. 2014
pcbd-1	transcriptional	~800	OH12385	Zhang et al. 2014
qdpr-1	transcriptional	~700	OH12386	Zhang et al. 2014
gfrp-1	translational, exon 2	~2500 (~1400)	OH12387	Zhang et al. 2014

** - length of sequence upstream to GFP (length upstream of predicted translation start if translational fusion).
† - this strain shows expression in biogenic amine neurons and the *epidermis, not in muscle cells* as reported.
